# Plant stress physiology under environmental emerging contaminant exposure: from molecular responses to phytoremediation applications

**DOI:** 10.3389/fpls.2026.1877141

**Published:** 2026-07-27

**Authors:** Marcelo Pedrosa Gomes, Leila Teresinha Maranho, Letícia Estela Cavichiolo Espindola, Flávia Yoshie Yamamoto

**Affiliations:** Laboratório de Fisiologia de Plantas sob Estresse, Departamento de Botânica, Setor de Ciências Biológicas, Universidade Federal do Paraná, Curitiba, Paraná, Brazil

**Keywords:** holobiont-mediated tolerance, internal exposure dynamics, phytohormones, phytoremediation, reactive oxygen species

## Abstract

The increasing occurrence of emerging environmental contaminants (EECs) in terrestrial and aquatic ecosystems is reshaping plant physiological performance, with implications extending from cellular metabolism to ecosystem function and phytoremediation performance. Pharmaceuticals, pesticides, PFAS, nanomaterials, and plastic-derived particles differ substantially in their physicochemical properties, uptake behavior, intracellular mobility, and biological targets, resulting in highly variable physiological outcomes. Here, we synthesize recent advances in plant stress physiology under EEC exposure through an integrated mechanistic framework linking contaminant uptake and translocation, detoxification pathways, oxidative stress dynamics, phytohormonal signaling, microbiome interactions, and translational phytoremediation strategies. Evidence compiled across contaminant classes demonstrates that plant toxicity is primarily governed by internal exposure dynamics rather than external concentrations alone. Across studies, plant responses frequently exhibit nonlinear physiological patterns, including antioxidant enzyme induction ranging from approximately 1.5- to 4.6-fold, glutathione depletion of 20–50%, nitrate reductase inhibition of 20–60%, and contaminant-specific hormonal responses varying from moderate abscisic acid increases (2–3-fold) to >10-fold jasmonate accumulation under severe stress conditions. Physicochemical properties, vascular transport constraints, and rhizospheric interactions collectively determine the compartmentalization of contaminants, ROS generation, metabolic disruption, and stress signaling outcomes. Oxidative stress emerges as a recurrent integrative regulatory interface linking xenobiotic perception with hormonal reprogramming, detoxification processes, and physiological acclimation, although its magnitude and mechanistic contribution remain dependent on the contaminant and tissue. Importantly, the literature reveals strong nonlinearities in plant responses to contaminant mixtures and co-occurring climate-related stressors, highlighting the limitations of reductionist single-stressor approaches. Recent findings have further demonstrated that plant-associated microbiota substantially influence contaminant fate, redox balance, and phytoremediation efficiency, supporting the concept of holobiont-mediated contaminant tolerance. Finally, advances in omics technologies, microbiome engineering, ecological treatment systems, and predictive modeling are progressively transforming phytoremediation into a physiology-informed and engineerable nature-based solution. Collectively, this review establishes a systems-level framework connecting contaminant transport, redox regulation, hormonal signaling, and microbiome functionality, providing mechanistic insights essential for environmental monitoring, risk assessment, and sustainable remediation under global-change scenarios.

## Graphical Abstract

**Figure:**
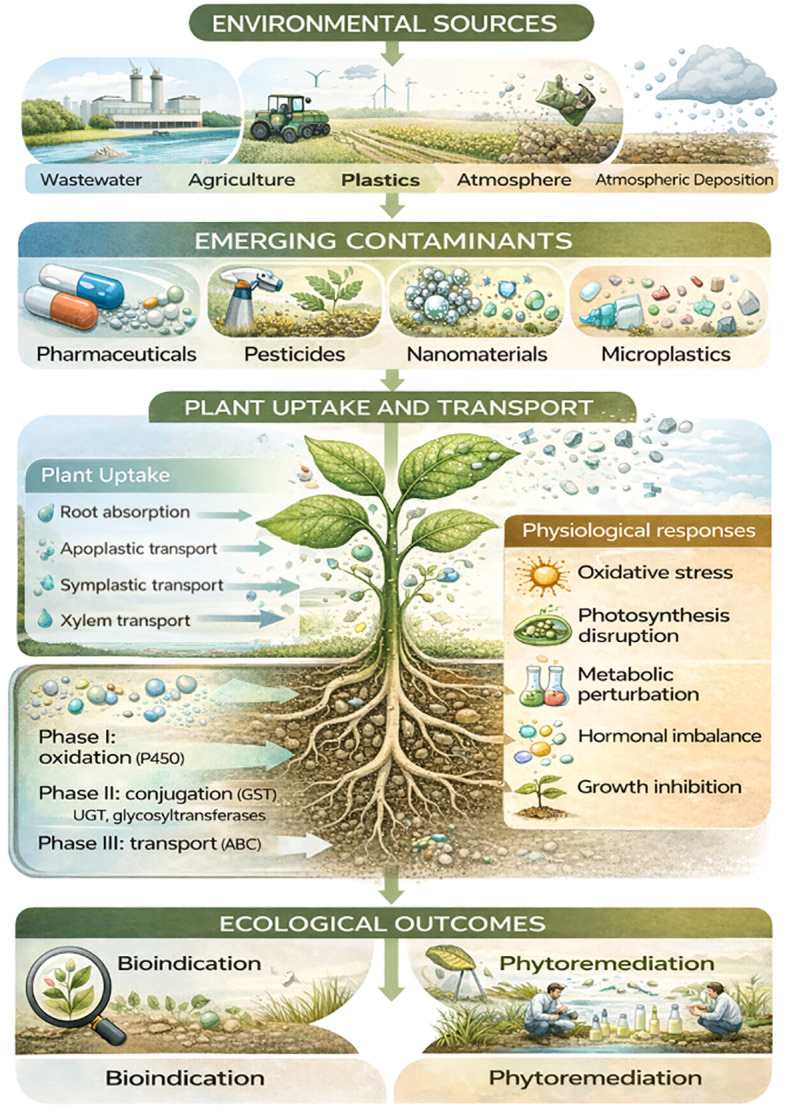

## 1 Introduction

The exponential expansion of anthropogenic chemical production has introduced an unprecedented diversity of synthetic compounds into natural ecosystems, fundamentally reshaping the chemical landscape during the Anthropocene. Among these, emerging environmental contaminants (EECs), including pharmaceuticals, personal care products, pesticides, plastic-derived particles, engineered nanomaterials, and industrial chemicals, have gained increasing attention because many exhibit environmental persistence, biological activity at environmentally relevant concentrations, and continuous release into terrestrial and aquatic ecosystems. Current global inventories indicate that more than 350,000 chemicals and mixtures are registered for production and use, many of which ultimately enter soils, freshwater systems, and the atmosphere through wastewater discharge, agricultural runoff, biosolid application, and atmospheric deposition (UNEP, 2019). Although only a subset of these substances is currently classified as EECs, the term generally refers to compounds that are increasingly detected in the environment, remain insufficiently regulated or monitored, and raise growing ecological or toxicological concern, often in the absence of established environmental thresholds (Boahen et al., 2025).

Mounting evidence demonstrates that EECs are ubiquitous across global freshwater and terrestrial systems. Large-scale monitoring studies have revealed the widespread detection of pharmaceutical residues in rivers worldwide, with a substantial proportion of sites exceeding the predicted safe thresholds for aquatic organisms (Wilkinson et al., 2022). Similarly, antibiotics, endocrine-disrupting compounds, and plastic-derived pollutants have been reported in irrigation waters and agricultural soils, where they can accumulate and interact with plant systems over extended periods (Gomes et al., 2022a; Marques et al., 2024; Trisotto et al., 2025). These contaminants are typically present at low concentrations (ng–µg L^-^¹ or µg kg^-^¹); however, their chronic exposure, chemical diversity, and potential for mixture interactions create complex and poorly understood biological effects. Despite the rapid expansion of monitoring studies, important knowledge gaps remain regarding the environmental behavior and biological relevance of many contaminant classes, particularly under realistic multi-contaminant exposure scenarios and in terrestrial ecosystems, where available information remains comparatively limited.

Plants are a major biological interface connecting the soil, water, and atmospheric compartments in contaminated environments. As sessile primary producers, they function as biological interfaces connecting the soil, water, and atmospheric compartments, continuously interacting with environmental contaminants throughout their life cycle. Plants absorb, transform, and redistribute xenobiotics through root uptake, foliar absorption, and vascular transport, thereby influencing contaminant mobility, ecological cycling, and trophic transfer (Wei et al., 2023a). Importantly, contaminant toxicity in plants is increasingly recognized as being governed not only by external concentrations but also by internal exposure dynamics, including uptake efficiency, tissue compartmentalization, intracellular mobility, and metabolic persistence (Wei et al., 2023c; Mallin et al., 2025). Consequently, contaminants present at similar environmental concentrations may generate substantially different physiological outcomes, depending on their internal fate within plant tissues. At the same time, plants possess a highly dynamic physiological system capable of responding to chemical stress through coordinated processes involving detoxification pathways, redox regulation, metabolic adjustment, and signaling networks.

Despite rapid advances in the field, our current understanding of plant responses to EECs remains fragmented and often constrained by reductionist experimental approaches. Most studies focus on single contaminants and isolated physiological endpoints, such as growth inhibition, oxidative stress markers, or enzymatic responses, without capturing the integration of these processes across scales. A systematic review found that 93% of 579 studies examined single stressors, with only 7% assessing the effects of multiple stressors (O’Brien et al., 2019). This has led to an incomplete understanding of how plants respond to contaminant exposure as dynamic, system-level phenomena governed by interactions between redox homeostasis, metabolism, signaling networks, and environmental contexts (Li et al., 2025). Moreover, real-world exposure scenarios involve complex mixtures of contaminants and co-occurring stressors, including climate-related factors such as drought, elevated temperatures, and ultraviolet radiation, which can modulate contaminant uptake, bioavailability, and toxicity (Gomes, 2024a; Castilho et al., 2025). Furthermore, direct comparisons among published studies remain challenging because experimental designs vary substantially with respect to plant species, developmental stage, exposure duration, contaminant concentrations, culture systems, and physiological endpoints. Consequently, some apparent discrepancies in the literature may reflect methodological heterogeneity rather than genuine biological inconsistencies, highlighting the need for more standardized experimental approaches and integrative physiological analyses.

A central integrative component of plant responses to EECs is oxidative stress, which functions as a key physiological regulatory interface. Reactive oxygen species (ROS), including superoxide radicals (O_2_•^-^), hydrogen peroxide (H_2_O_2_), and hydroxyl radicals (•OH), are not merely byproducts of cellular damage but also serve as crucial signaling molecules that link contaminant perception to downstream physiological processes (Averill-Bates, 2024). ROS dynamics regulate antioxidant defense systems, modulate metabolic fluxes, and interact with hormonal signaling networks, thereby shaping plant acclimation and injury response (Averill-Bates, 2024). However, the dual role of ROS as both signaling agents and mediators of oxidative damage complicates their interpretation, underscoring the need for integrative frameworks that extend beyond endpoint-based analyses of ROS.

In addition to redox regulation, plant responses to contaminants involve extensive metabolic and signaling reprogramming processes. Alterations in carbon and nitrogen metabolism, shifts in secondary metabolite production, and activation of detoxification pathways collectively contribute to plant tolerance (Khan et al., 2025). Concurrently, phytohormones such as abscisic acid, salicylic acid, jasmonates, auxins, and ethylene orchestrate stress responses through complex crosstalk with ROS-dependent signaling pathways, coordinating transcriptional and physiological adjustments (Jardim-Messeder et al., 2025). These interactions demonstrate that contaminant stress must be understood as a multi-layered process that integrates biochemical, cellular, and systemic responses. Another critical dimension of plant responses to EECs involves interaction with the plant-associated microbiome. Rhizosphere and endophytic microorganisms can influence contaminant bioavailability, participate in xenobiotic degradation, and modulate plant stress responses through metabolic and signaling interactions (Hu et al., 2023). This plant–microbiome interface adds an additional layer of complexity, linking plant physiology to ecosystem-level processes and reinforcing the need for a holistic approach.

In parallel with their ecological implications, plant responses to emerging contaminants are significantly relevant for environmental monitoring and remediation. Plants integrate environmental signals over time, making them valuable bioindicators of environmental contamination. Furthermore, plant-based remediation strategies, including phytoextraction, phytostabilization, and rhizodegradation, are increasingly recognized as sustainable approaches for mitigating environmental pollution (Gomes, 2024a). However, the effectiveness of these strategies depends on a mechanistic understanding of plant stress physiology under realistic exposure conditions. In this review, a unified physiological framework is proposed in which oxidative stress is conceptualized as a central regulatory hub linking contaminant exposure to metabolic reprogramming, hormonal signaling, and plant adaptive responses. By integrating evidence across contaminant classes, biological scales, and environmental contexts, this study advances a systems-level perspective on plant stress physiology under exposure to emerging contaminants. In addition to synthesizing current knowledge, the review critically discusses areas of consensus, methodological limitations, and unresolved questions that continue to constrain mechanistic interpretation and predictive understanding of plant responses to emerging contaminants.

The following sections examine the mechanisms governing contaminant uptake and translocation, cellular detoxification pathways, oxidative stress dynamics, antioxidant responses, metabolic adjustments, and signaling networks, culminating in a critical evaluation of plant-based strategies for environmental monitoring and phytoremediation within the One Health framework. Literature screening was conducted using a structured but non-systematic approach combining database searches and citation tracking. Primary searches were performed using Web of Science, Scopus, PubMed, and Google Scholar, complemented by the Elicit platform for AI-assisted literature discovery and thematic clustering analysis. Searches prioritized studies published between 2019 and 2026, although earlier landmark studies were retained when they were mechanistically relevant. The search terms included combinations of *emerging contaminants*, *plant ecotoxicology*, *oxidative stress*, *phytohormones*, *phytoremediation*, *nanoplastics*, *PFAS*, *microbiome*, *constructed wetlands*, *ROS signaling* and *plant physiology*. Priority was given to experimental studies reporting physiological, biochemical, hormonal, or contaminant fate endpoints in plants exposed to emerging contaminants. Reviews, editorials, and studies lacking mechanistic or quantitative physiological information were preferentially excluded from the quantitative synthesis tables, although some were retained to support the conceptual discussion when directly relevant.

## 2 Emerging environmental contaminants and plant responses

The increasing occurrence of EECs in terrestrial and aquatic ecosystems has introduced a complex spectrum of chemical stressors that interact with plant physiological systems in fundamentally distinct ways. These contaminants comprise chemically diverse groups, including pharmaceuticals, pesticides, plastic-derived particles, engineered nanomaterials, industrial compounds, and endocrine-disrupting chemicals, each characterized by specific physicochemical properties that govern their environmental behavior and biological interactions (Aziz et al., 2026). Rather than inducing uniform stress responses, EECs affect plants through multiple partially overlapping mechanisms that depend on contaminant identity, concentration, exposure duration, and environmental context.

The interaction between contaminants and plant systems begins with the processes governing environmental mobility and bioavailability. Molecular polarity, ionization state, hydrophobicity (log K_ow_), and molecular size influence the partitioning of contaminants between soil, water, and plant tissues, thereby determining their uptake efficiency and internal distribution (Li, 2022; Wei et al., 2023a). Root uptake is the dominant exposure pathway in most terrestrial and aquatic systems, mediated by passive diffusion, carrier transport, and apoplastic flow (Li, 2022). However, foliar uptake through cuticular diffusion or stomatal entry may also contribute significantly to atmospheric exposure scenarios (Avellan et al., 2021; Yu et al., 2024). Once internalized, contaminants can be translocated via the xylem and phloem to metabolically active tissues, where they interact with cellular targets.

Plant responses to different contaminant classes can be broadly interpreted through three interconnected physiological domains that provide a mechanistic framework for understanding the stress responses. The first domain comprises redox-driven processes, in which contaminants induce ROS generation by disrupting electron transport chains, surface redox reactions, or metabolic interference. The second domain involves metabolism-driven responses characterized by perturbations in carbon and nitrogen assimilation, energy balance, and secondary metabolism. The third domain encompasses signaling-driven responses mediated by hormonal networks and stress-signaling pathways that coordinate adaptive or maladaptive responses. These domains are not independent but interact dynamically to form an integrated physiological network that determines plant performance under contaminant exposure.

Different contaminant classes preferentially target specific components of the networks. For example, herbicides and pesticides often exert primary effects on photosynthetic machinery or key metabolic pathways, leading to rapid photochemical disruption and downstream oxidative stress (Gomes et al., 2019a; Yildiztugay et al., 2022). In contrast, engineered nanomaterials frequently trigger stress responses through particle–membrane interactions, catalytic surface activity, and nanoscale physicochemical reactivity, affecting cellular redox balance, gene expression, and protein functionality (Wang et al., 2024; Xu et al., 2024). Importantly, oxidative stress induced by engineered metal nanomaterials differs mechanistically from that caused by their dissolved ionic counterparts. Dissolved metals generally promote ROS formation indirectly through intracellular ion toxicity, metabolic disruption, and interference with redox-active proteins. By contrast, nanomaterials may generate ROS directly at the particle interface via surface-mediated redox reactions. In metal-based nanoparticles such as ZnO, CuO, or AgNPs, high surface area-to-volume ratios, catalytic surface properties, and localized particle–membrane interactions can accelerate oxidative damage, often resulting in faster or spatially distinct physiological responses relative to dissolved metal forms (Wang et al., 2023). Furthermore, many metallic nanoparticles undergo partial dissolution following plant uptake or at the root interface, generating a dual toxicity mechanism that combines nanoparticle-specific physical interactions with ionic toxicity from released metal species, as reported for cobalt nanoparticles in *Lemna minor* (Mallin et al., 2025). Consequently, nanoparticle exposure may simultaneously disrupt redox homeostasis, membrane integrity, nutrient uptake, and transcriptional regulation, reinforcing their capacity to perturb multiple interconnected physiological networks. Pharmaceuticals, in turn, tend to interfere more subtly with metabolic and signaling pathways, particularly under chronic low-dose exposure, where persistent physiological disturbances accumulate over time (Gomes et al., 2020, Gomes et al., 2022c). Plastic-derived contaminants, including micro- and nanoplastics, add further complexity by acting not only as physical stressors but also as vectors for co-contaminants, thereby altering contaminant bioavailability and potentially amplifying combined toxicological effects (Du et al., 2025).

These mechanistic differences are reflected in the physiological responses, as summarized in **Table 1**. While oxidative stress, photosynthetic impairment, and metabolic disruption are commonly reported across contaminant classes, the underlying pathways leading to these outcomes differ substantially among contaminants. For instance, nanomaterial-induced oxidative stress often originates from surface redox reactions and membrane interactions, whereas herbicide-induced oxidative stress is typically a secondary consequence of inhibited photosynthetic electron transport (Wang et al., 2024; Xu et al., 2024). Similarly, pharmaceuticals may disrupt cellular metabolism without directly targeting primary energy pathways, leading to delayed but functionally significant physiological effects (Gomes et al., 2020, Gomes et al., 2022c).

**Table 1:** Major classes of emerging environmental contaminants detected in terrestrial and aquatic ecosystems, their principal physiological effects on plants, and ecological implications.

Contaminant class	Representative compounds	Major environmental sources	Primary plant exposure pathways	Key physiological effects in plants	Potential ecological implications	Representative references
Pharmaceuticals and personal care products (PPCPs)	Antibiotics (ciprofloxacin, sulfamethoxazole), metformin, antidepressants	Wastewater effluents, sewage sludge, hospital discharge	Root uptake; occasional foliar deposition	Disruption of metabolism, oxidative stress, interference in signaling pathways	Bioaccumulation, trophic transfer, altered plant growth	(Leitão et al., 2021a, Leitão et al., 2021b; Kitamura et al., 2023)
Pesticides and herbicides	Glyphosate, atrazine, dicamba, chlorpyrifos	Agricultural runoff, spray drift	Root uptake; foliar absorption	Photosynthetic inhibition, ROS overproduction, metabolic disruption	Reduced productivity, ecological imbalance	(Soares et al., 2021; Gomes et al., 2022b; Sobiecka et al., 2022)
Industrial chemicals and phenolic contaminants	BPA, PAHs, p-nitrophenol	Industrial discharge, plastic degradation	Root uptake, sorption	Membrane damage, oxidative imbalance, root growth inhibition	Bioaccumulation and ecosystem toxicity	(Malea et al., 2022; Abdelmoneim et al., 2024; Chakraborty et al., 2026)
Plastic-derived contaminants and microplastics	Polyethylene, polystyrene, nanoplastics	Plastic degradation, sludge application	Root interface adsorption; indirect uptake	Root architecture alteration, oxidative stress, carrier effect for co-contaminants	Soil structure alteration, contaminant vectorization	(Arikan et al., 2022; Yildiztugay et al., 2022; Xu et al., 2024; Xie et al., 2026)
Engineered nanomaterials	ZnO, TiO_2_, Ag nanoparticles, CNTs	Industrial emissions, nano-enabled products	Root uptake, foliar entry	Surface-mediated ROS generation, membrane damage, gene expression changes	Phytotoxicity, uncertain long-term ecological effects	(Molnár et al., 2020b; Yusefi-Tanha et al., 2020; Manna et al., 2021; Voloshina et al., 2022)
Endocrine-disrupting compounds	Estradiol, ethinylestradiol, BPA	Wastewater, livestock waste	Root uptake	Hormonal disruption, altered development and signaling	Reproductive and developmental effects	(Malea et al., 2022; Ozfidan-Konakci et al., 2023; Abdelmoneim et al., 2024)

Responses summarize representative patterns reported under environmentally relevant or experimentally realistic exposure scenarios.

Although the physiological patterns summarized in **Table 1** are recurrent across contaminant classes, they should not be interpreted as universal responses. Direct comparisons among studies remain challenging because experimental designs differ substantially in plant species, developmental stage, exposure duration, contaminant concentrations, growth systems (e.g., hydroponic versus soil), and measured physiological endpoints. Consequently, some apparent differences among contaminant classes may reflect methodological heterogeneity rather than intrinsic mechanistic differences. Future studies using standardized experimental protocols and directly comparing multiple contaminant classes under identical experimental conditions will be essential to distinguish contaminant-specific responses from experimental variability.

Importantly, the magnitude and nature of plant responses are not solely determined by contaminant properties but are strongly influenced by biological and environmental factors. Species-specific traits, including root architecture, membrane composition, metabolic capacity, and detoxification potential, influence the uptake and tolerance of contaminants (Maranho and Gomes, 2024). The developmental stage also plays a critical role, as younger tissues often exhibit higher metabolic activity and greater sensitivity to stress (Pande et al., 2025). Environmental conditions, including temperature, light intensity, nutrient availability, and water status, modulate contaminant behavior and plant responses, often amplifying or mitigating stress effects (Gomes, 2024b; Castilho et al., 2025).

To capture this complexity, a wide range of physiological, biochemical, and molecular biomarkers has been developed to assess plant responses to EECs. These biomarkers, summarized in **Supplementary Table 1**, span multiple levels of biological organization, from molecular and cellular processes to whole plant performance. Growth parameters and photosynthetic metrics provide integrative indicators of plant performance, whereas oxidative stress markers and antioxidant enzyme activity reflect redox balance. Detoxification enzymes, nitrogen metabolism indicators, and phytohormone profiles offer insights into metabolic and regulatory responses, whereas omics-based approaches enable system-level analyses of plant responses. Importantly, these biomarkers do not operate independently but represent interconnected components of the physiological networks described. No individual biomarker is sufficient to characterize plant responses to EEC exposure. Many physiological endpoints, including oxidative stress markers, antioxidant enzymes, photosynthetic performance, and hormone profiles, respond dynamically according to contaminant identity, exposure duration, tissue type, and plant species. Therefore, integrated interpretation of multiple biomarkers provides a more robust assessment of plant stress than isolated measurements, allowing discrimination between transient acclimation responses and progressive physiological impairment.

Despite considerable progress in identifying contaminant-induced physiological responses, important knowledge gaps remain. Most currently available studies are based on single-contaminant laboratory experiments, whereas plants in natural ecosystems are simultaneously exposed to complex contaminant mixtures under fluctuating environmental conditions. Furthermore, differences in experimental methodologies often limit direct comparison among studies and hinder the establishment of generalized physiological thresholds. These limitations emphasize the need for integrative experimental approaches capable of linking multiple stressors, internal contaminant dynamics, and coordinated physiological responses under environmentally realistic conditions.

Taken together, these observations demonstrate that plant responses to emerging contaminants must be understood as integrated, multi-scale processes shaped by the interactions between contaminant properties, plant physiology, and environmental context. This perspective highlights the need for mechanistic frameworks that move beyond descriptive approaches and incorporate the interconnected nature of redox regulation, metabolism, and signaling. The following sections build on this foundation by examining the processes governing contaminant uptake and translocation, detoxification pathways, oxidative stress dynamics, antioxidant responses, and the integration of signaling networks in plant stress physiology.

## 3 Uptake and translocation of micropollutants in plants

The uptake and internal redistribution of micropollutants in plants constitute a hierarchically regulated process, in which physicochemical constraints, anatomical barriers, and vascular transport dynamics converge to define internal exposure patterns. Rather than reflecting external concentrations, contaminant accumulation within plant tissues emerges from a sequence of physiological and anatomical barriers, including root interface interactions, membrane transport, and long-distance translocation, whose combined effects determine the spatial distribution and, ultimately, the biological impact (**Supplementary Table 2**).

### 3.1 Physicochemical constraints and non-linear uptake behavior

The entry of contaminants into plant tissues is fundamentally governed by partitioning behavior, ionization equilibrium, and molecular size. However, the empirical evidence synthesized in **Supplementary Table 2** reveals that the uptake efficiency follows a nonlinear pattern, with contaminant transfer often being optimized within relatively narrow physicochemical ranges. Compounds such as ciprofloxacin (log K_ow_ 0.28) exhibit strong speciation-dependent uptake, where increasing pH reduces absorption due to enhanced ionization, demonstrating that membrane permeability is subordinated to electrochemical constraints rather than hydrophobicity (Hu et al., 2021). This nonlinearity is further reinforced by class-dependent behaviors. For example, in PFAS, accumulation does not follow classical partitioning predictions but instead exhibits a chain-length-dependent sigmoidal response, where root concentration factors increase markedly with molecular size, reaching values up to ~100 in emerging compounds such as HFPO-TA (Dal Ferro et al., 2021). This pronounced enrichment likely reflects the combined effects of strong ionic interactions with root tissues, limited membrane permeability, and restricted xylem loading.

Unlike shorter-chain or more mobile PFAS homologues, HFPO-TA exhibits high root retention owing to its physicochemical characteristics that favor sorption to cell wall polysaccharides and membrane-associated interfaces, while simultaneously limiting long-distance vascular transport. In addition, steric constraints and strong anionic behavior may reduce symplastic redistribution, effectively concentrating these compounds within root compartments (Dal Ferro et al., 2021). Although the relative contributions of these mechanisms remain incompletely resolved, current evidence supports a transport-limited accumulation process rather than simple passive partitioning. Collectively, these findings indicate that sorption affinity and transport constraints can override diffusion-based uptake models, creating threshold-like transitions between mobile and retained contaminant fractions.

Although these physicochemical relationships are consistently observed across multiple contaminant classes, their quantitative expression varies considerably among plant species and experimental conditions. Root architecture, membrane composition, rhizosphere chemistry, and growth systems can substantially modify contaminant partitioning and transport, making direct comparisons among studies difficult. Consequently, current uptake models should be interpreted as mechanistic frameworks rather than universally applicable predictive models, emphasizing the need for comparative studies conducted under standardized experimental conditions.

### 3.2 Root interface: adsorption-controlled entry and radial transport limitation

The root interface is the primary gateway for most contaminants and acts as a major kinetic constraint that controls internalization. The data in **Supplementary Table 2** demonstrate that uptake is frequently governed by adsorption-controlled processes rather than by rapid transmembrane penetration. Ciprofloxacin exemplifies this mechanism, with uptake characterized by pseudo-first-order adsorption onto root tissues during the early exposure phases (≤72 h), followed by a gradual transfer to the xylem (Hu et al., 2021). This adsorption-dominated regime imposes a temporal lag between exposure and systemic distribution, effectively decoupling the external concentration from the internal dose. Similar constraints have been observed for PFAS, where strong interactions with root tissues lead to pronounced accumulation gradients (Dal Ferro et al., 2021). Root concentration factors ranging from 35.7 to 99.9 for certain compounds indicate that the root system functions not merely as an entry point but as a selective retention compartment.

The transition across the endodermis introduces an additional layer of selectivity by interrupting the unrestricted apoplastic movement and forcing contaminants to cross the cellular membranes before entering the vascular tissues (Mei et al., 2021; Shukla and Barberon, 2021). As a result, transport becomes strongly dependent on the membrane permeability, ionization state, and interactions with membrane proteins. Polar and ionizable contaminants may partially rely on aquaporins, nodulin26-like intrinsic proteins (NIPs), ion channels, or transporter-mediated pathways to cross biological membranes, whereas hydrophobic compounds diffuse more frequently through lipid bilayers (Sabir et al., 2020). Although the transporter specificity for many EECs remains insufficiently resolved, increasing evidence suggests that membrane proteins contribute to the selective uptake and redistribution of contaminants beyond purely physicochemical partitioning mechanisms (Gomes et al., 2015; Tansel, 2026).

The effective pore size of the plant cell wall (typically 5–20 nm) restricts the passage of larger nanoparticles and excludes most microplastics. Experimental evidence further indicates a strong particle size dependency for intracellular entry. Nanomaterials approaching or below the empirical cell wall pore size range (~20 nm) exhibit substantially greater internalization efficiency through nanoscale pores, membrane invagination, or endocytosis-like processes. In contrast, larger particles (~100 nm) are more frequently retained in the apoplastic spaces or adsorbed onto the root surfaces (Sun et al., 2021; Wang et al., 2023). For instance, nanoparticles below 20 nm exhibit greater foliar delivery to guard cells, extracellular spaces, and chloroplasts compared with larger particles (Hu et al., 2020). This difference in entry efficiency may also explain the divergent physiological responses among particle sizes, as smaller nanoparticles are more likely to interact directly with intracellular organelles and redox-sensitive pathways, whereas larger particles often exert stronger extracellular or mechanical effects. However, these patterns remain highly dependent on the particle charge, aggregation behavior, coating chemistry, and plant species. Consequently, particle size strongly influences whether nanomaterials undergo systemic redistribution or remain largely immobilized at the root interface, creating steep concentration gradients between the external and internal tissues (Wang et al., 2023). This results in a clear bifurcation between compounds capable of systemic redistribution and those that are effectively immobilized at the root level.

### 3.3 Translocation dynamics and organ-specific accumulation patterns

Once internalized, the fate of the contaminant becomes strongly dependent on the efficiency of vascular translocation, which determines whether the compounds remain confined to the root tissues or are redistributed to the metabolically active aerial organs. This process is commonly expressed as the shoot-to-root translocation factor (TF). The data compiled in **Supplementary Table 2** reveal pronounced variability in TF across contaminant classes, reflecting the underlying differences in molecular size, polarity, and vascular accessibility.

Perfluoroalkyl substances provide a paradigmatic example of size-dependent partitioning. Short-chain PFAS (≤C7) are preferentially translocated to aerial tissues, whereas long-chain homologues (≥C8) exhibit strong root retention, resulting in an inverse relationship between TF and molecular volume (Dal Ferro et al., 2021). Similarly, PFOA preferentially accumulated in shoots (up to 77.4% of the total mass), whereas PFOS remained largely confined to roots (up to 95.8%) (Wang et al., 2020). For systemic pesticides, such as imidacloprid, efficient xylem-mediated transport enables rapid redistribution to shoots during early developmental stages, regardless of whether uptake occurs via seed or root pathways (Tang et al., 2020). Pharmaceuticals with moderate polarity often exhibit TF values between ~0.5 and 3, reflecting efficient transfer to the xylem and subsequent shoot accumulation (Carter et al., 2014; Bigott et al., 2021). In contrast, strongly sorbing antibiotics, such as tetracyclines, display TF values <0.5, with pronounced retention in root tissues due to cell wall binding and limited symplastic entry (El Gemayel and Bashour, 2020).

Collectively, these patterns indicate that translocation efficiency is not solely governed by uptake pathways but also by the interaction between contaminant physicochemical properties and vascular loading constraints. Consequently, compounds with similar external exposure levels may exhibit markedly different organ-specific accumulation profiles, reinforcing the notion that internal exposure dynamics are determined more by transport continuity and tissue accessibility than by environmental concentrations alone.

### 3.4 Foliar uptake and directional asymmetry in vascular transport

Although root uptake dominates in most systems, foliar entry introduces an additional dimension of complexity, particularly for particulate contaminants and surface-deposited compounds. Nanoplastics can enter leaves through stomatal pathways and subsequently translocate to the roots via vascular bundles (Sun et al., 2021). However, this transport is strongly constrained by particle aggregation and surface charge, with positively charged particles exhibiting enhanced retention in leaf tissues and reduced rootward movement (Sun et al., 2021).

Directional asymmetry was also evident in the dissolved compounds. For instance, triflumezopyrim exhibits efficient upward transport following root uptake but shows no detectable downward redistribution after foliar exposure (Fan et al., 2020). This asymmetry likely reflects the limited phloem loading efficiency. While upward movement through the xylem is facilitated by transpiration-driven flow following root uptake, downward redistribution depends on effective entry into companion cells and phloem tissues, a process that is strongly influenced by molecular polarity, ionization behavior, and membrane permeability (Fan et al., 2020). Therefore, compounds exhibiting restricted phloem mobility may accumulate in exposed leaves without substantial redistribution to roots or developing tissues, creating pronounced directional asymmetry in vascular transport. Collectively, these observations indicate that vascular mobility is not universally accessible but emerges from selective interactions between contaminant properties and transport systems, ultimately determining the extent of systemic redistribution.

### 3.5 Size-dependent transport constraints in particulate contaminants

Particulate contaminants introduce transport mechanisms that are fundamentally distinct from those governing dissolved molecules. Sub-micrometer plastics can enter root tissues via crack-entry pathways into the stele, followed by transpiration-driven upward movement (Li et al., 2020a; Sun et al., 2021). However, this process is highly size-dependent, with the uptake efficiency increasing sharply as the particle size decreases. Smaller particles are more likely to penetrate intracellular compartments and interact directly with organelles and redox-sensitive pathways, whereas larger particles tend to remain associated with extracellular spaces or root surfaces, exerting stronger physical and mechanical effects (Hu et al., 2024; Zhang et al., 2024). Consequently, particle size influences not only transport efficiency but also the dominant mechanism underlying toxicity, creating a gradient from predominantly intracellular biochemical disruption to predominantly extracellular physical interference.

This divergence is reflected in the physiological outcomes. Experimental evidence indicates that smaller particles (<100 nm) predominantly induce toxicity through oxidative stress pathways, whereas larger microplastics exert their effects by disrupting hormonal homeostasis, particularly auxin signaling (Sun et al., 2021). Correspondingly, reductions in root length of up to 55.1% and shoot biomass losses exceeding 40% have been associated with microplastic-induced depletion of indole-3-acetic acid (IAA) (Li et al., 2020a). Although the precise mechanism remains unclear, emerging evidence suggests that microplastic-induced auxin depletion is unlikely to result solely from the physical obstruction of transport pathways. Instead, particulate stress may interfere with auxin homeostasis by disrupting polar auxin transport systems, particularly PIN-mediated auxin redistribution, membrane destabilization, and ROS-mediated signaling interference (Ma et al., 2025). Oxidative stress may further impair auxin gradients by altering transporter localization and membrane function, thereby contributing to reduced root elongation and biomass accumulation. Thus, microplastic-induced growth inhibition likely reflects a combined effect of physical interference and signaling disruption, rather than simple vascular blockage alone.

### 3.6 Integrative framework: internal distribution as determinant of biological effect

The integration of physicochemical constraints, anatomical barriers, and vascular transport dynamics leads to a central mechanistic implication: internal distribution, rather than external exposure, defines the impact of contaminants on plant health. The evidence synthesized in **Supplementary Table 2** indicates that the biological relevance of contaminants is determined by their ability to overcome sequential transport barriers and reach physiologically active tissues in plants. Compounds with high translocation efficiency and vascular mobility are more likely to affect photosynthetic tissues, disrupt hormonal balance, and induce systemic stress responses. In contrast, strongly retained compounds may accumulate in the roots at high concentrations without producing equivalent systemic effects.

Collectively, the uptake and translocation of emerging contaminants are governed by a coupled system of physicochemical filtration, anatomical control, and vascular redistribution. This system generates nonlinear, compound-specific exposure patterns that propagate into hormonal and oxidative signaling networks, ultimately shaping plant responses to environmental contaminants. By linking quantitative descriptors of uptake (kinetics and BCF) and translocation (TF) with physiological outcomes, this mechanistic perspective establishes a conceptual bridge between environmental exposure and biological effects. This relationship provides a foundation for interpreting downstream processes such as ROS signaling and phytohormonal reprogramming.

### 3.7 From internal exposure to physiological burden

While uptake, translocation, and tissue accumulation determine the spatial distribution of contaminants within plants, these processes alone do not necessarily predict the toxicological outcomes. Similar external concentrations may induce markedly different physiological responses depending on contaminant bioavailability, tissue-specific accumulation, intracellular compartmentalization, transformation processes, and the capacity of plants to buffer stress through detoxification and repair. Consequently, physiological impairment may be better understood as a function of biologically effective internal exposure rather than external concentration alone. Increasing evidence indicates that plant responses to emerging environmental contaminants are more strongly associated with internal exposure, which is effectively achieved after uptake, sequestration, transport, and metabolic transformation, than with nominal environmental concentrations (Wei et al., 2023c; Mallin et al., 2025).

This distinction is particularly relevant because externally defined toxicological thresholds, such as the lowest observed effect concentration (LOEC), describe applied environmental concentrations but do not necessarily capture the biologically relevant fraction that effectively reaches sensitive tissues and subcellular compartments of the organism. Thus, equivalent environmental concentrations may generate substantially different biological responses among species, organs, developmental stages, and environmental conditions owing to variations in contaminant uptake efficiency, vascular transport, sequestration, biotransformation, and metabolic fate.

Contaminant compartmentalization further modulates the physiological outcomes. Root-retained compounds may reach elevated tissue concentrations while exerting relatively localized effects due to limited vascular redistribution. In contrast, highly mobile contaminants can trigger systemic physiological disruption even at comparatively lower tissue concentrations if they effectively reach metabolically active tissues, such as chloroplast-rich leaves, meristems, or reproductive structures (Hauser-Davis, 2026). Similarly, subcellular localization strongly influences toxicity, as contaminants interacting with chloroplasts, mitochondria, plasma membranes, or redox-sensitive metabolic pathways are more likely to disrupt electron transport, hormonal signaling, and cellular homeostasis (Dreier et al., 2019; Duarte-Hospital et al., 2021).

Accordingly, the transition from internal exposure to physiological impairment may be conceptualized as a metabolic burden, in which toxicity emerges when contaminant accumulation exceeds the buffering capacity of antioxidant defenses, detoxification pathways, sequestration systems, and cellular repair processes. At lower effective internal exposure levels, plants may partially compensate for these effects through enzymatic detoxification, antioxidant activation, metabolic reprogramming, and hormonal adjustment. However, increasing the internal burden progressively elevates the energetic demands associated with xenobiotic transformation and stress signaling, potentially resulting in glutathione depletion, oxidative imbalance, metabolic disruption, and growth impairment. Collectively, the available evidence supports internal exposure dynamics as a more informative predictor of plant responses than external contaminant concentrations alone. Nevertheless, this conceptual framework is currently supported predominantly by controlled laboratory studies involving single contaminants. Whether similar transport dynamics prevail under environmentally realistic scenarios involving contaminant mixtures, heterogeneous soils, plant-associated microbiota, and fluctuating climatic conditions remains insufficiently explored. Addressing these limitations will be essential for improving the predictive capacity of plant ecotoxicology and for translating laboratory observations into environmental risk assessment and phytoremediation applications.

## 4 Cellular detoxification mechanisms under emerging contaminant exposure

Once contaminants overcome the uptake barriers and establish biologically effective internal exposure, detoxification becomes a critical determinant of whether physiological acclimation or toxicity prevails. In plants exposed to EECs, cellular detoxification is increasingly recognized not as an inherently efficient defense system but as a constrained, energetically demanding, and context-dependent metabolic process. Although traditionally described as a three-phase pathway—functionalization (Phase I), conjugation (Phase II), and sequestration (Phase III)—recent evidence demonstrates that these processes operate under strong biochemical and physiological limitations, particularly during chronic and mixture exposure scenarios (Li et al., 2023; Gomes et al., 2022c). Importantly, detoxification efficiency is determined not only by metabolic capacity but also by contaminant accessibility to enzymatic systems, intracellular compartmentalization, and competition for reducing equivalents required for both detoxification processes and oxidative stress mitigation.

Empirical studies further indicate that detoxification efficiency is highly variable and frequently incomplete. In plant-based treatment systems, the removal efficiencies for pharmaceuticals typically range from 20% to 80%, depending on the compound properties and exposure conditions (Li et al., 2023; Vymazal, 2019). However, persistent compounds, such as carbamazepine, consistently exhibit removal rates below 40%, even under optimized conditions, reflecting limited metabolic accessibility and the formation of stable transformation products (Zhang et al., 2023). Under these conditions, detoxification may progressively shift from an adaptive process to a metabolic burden, particularly when contaminant accumulation exceeds the buffering capacity of conjugation pathways, antioxidant systems, and sequestration mechanisms of the cell. These observations indicate that detoxification frequently results in transformation and accumulation rather than complete mineralization, challenging its interpretation as a fully protective process against EEC toxicity.

Importantly, detoxification efficiency should not be interpreted as an intrinsic indicator of plant tolerance. Similar contaminants may undergo markedly different metabolic fates depending on plant species, tissue type, developmental stage, and environmental conditions. Furthermore, enhanced detoxification enzyme activity does not necessarily translate into reduced toxicity, as transformation products may retain biological activity or generate new physiological constraints. These observations highlight that detoxification represents a dynamic balance between metabolic protection and metabolic cost rather than a universally beneficial process.

### 4.1 Phase I–III detoxification as an integrated network

**Figure 1:**
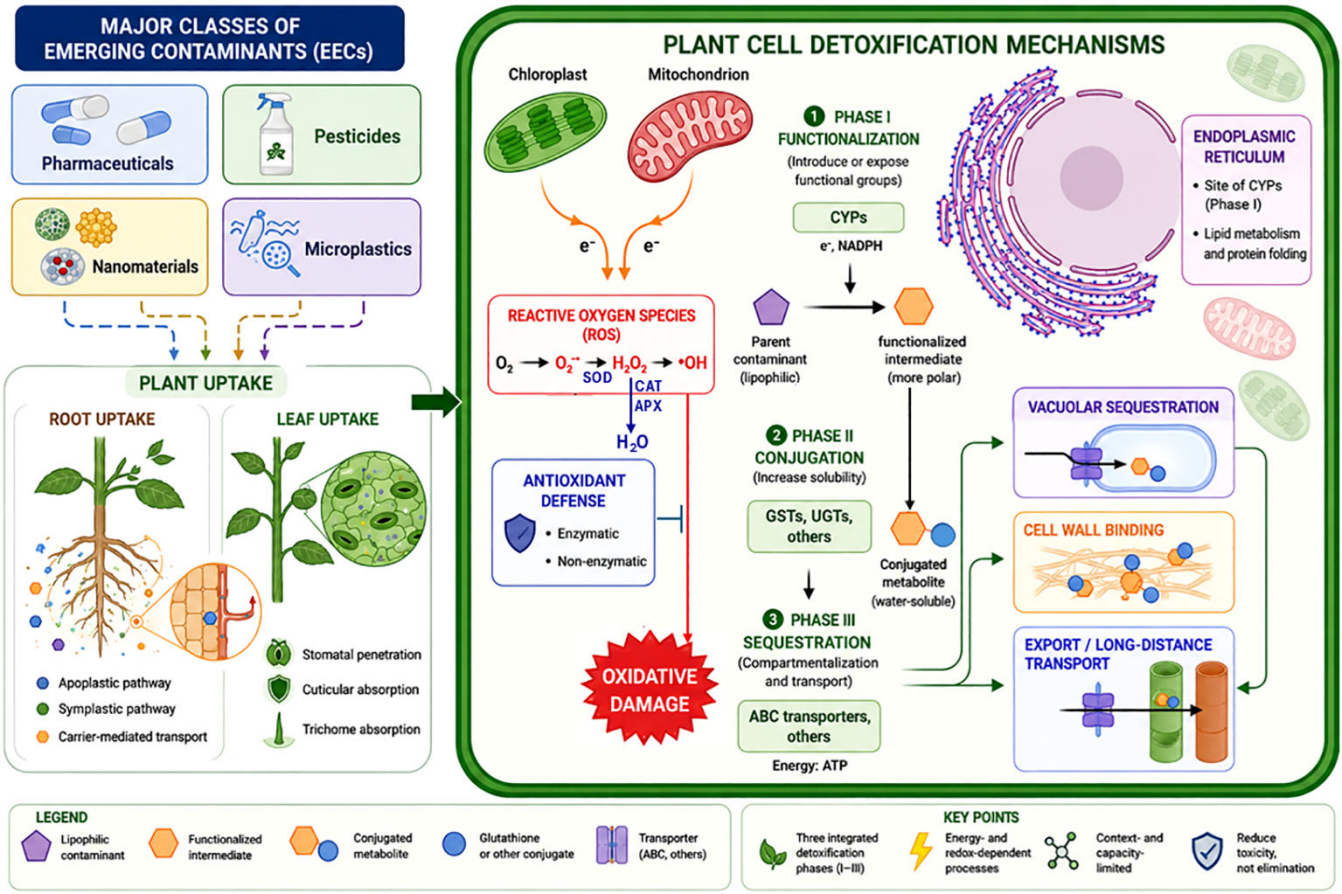
Integrated mechanistic framework of oxidative stress, detoxification, and toxification in plants exposed to emerging environmental contaminants (EECs). Pharmaceuticals, pesticides, herbicides, nanomaterials, and micro-/nanoplastics enter plants through root or foliar pathways and undergo tissue redistribution. Contaminants stimulate reactive oxygen species (ROS) production through multiple intracellular sources, including chloroplast electron transport, mitochondrial respiration, peroxisomal metabolism, and plasma membrane respiratory burst oxidase homologs (RBOHs). ROS accumulation triggers antioxidant defenses, including superoxide dismutase (SOD), catalase (CAT), ascorbate peroxidase (APX), peroxidase (POD), and the ascorbate–glutathione (AsA–GSH) cycle, and activates calcium-dependent and ROS-mediated signaling pathways involved in stress acclimation. Detoxification occurs through coordinated Phase I functionalization (e.g., CYP450-mediated metabolism), Phase II conjugation (e.g., glutathione and sugar conjugates), and Phase III sequestration (e.g., vacuolar compartmentalization, ABC transporters). Under certain exposure scenarios, Phase I metabolism may result in toxification through the generation of reactive or persistent intermediates (e.g., carbamazepine-10,11-epoxide) exhibiting equal or greater biological activity than the parent compound. When ROS production and metabolic burden exceed antioxidant and detoxification capacities, oxidative damage to lipids, proteins, DNA, and membranes occurs, ultimately impairing photosynthesis, growth, reproduction, and stress tolerance.

Phase I detoxification is primarily mediated by cytochrome P450 monooxygenases (CYPs), which catalyze oxidation reactions that increase compound polarity but frequently generate reactive intermediates (RIs) (**Figure 1**). For example, carbamazepine is transformed into carbamazepine-10,11-epoxide, a biologically active and potentially persistent metabolite (Zhang et al., 2021). Similarly, in aquatic plants, enrofloxacin can be metabolized into ciprofloxacin, demonstrating that Phase I reactions may produce equally persistent or more mobile compounds (Gomes et al., 2019b). Phase I reactions are intrinsically linked to cellular redox processes, as CYP-mediated electron transfer generates ROS, embedding detoxification within the broader oxidative stress network described in Section 5. Importantly, xenobiotic transformation does not necessarily reduce the toxicity. In some cases, Phase I metabolism may result in “toxification,” whereby transformation products exhibit equal or greater biological activity than the parent compound. However, this process is not universal and depends strongly on the contaminant chemistry, enzymatic specificity, tissue compartmentalization, and downstream conjugation efficiency. Carbamazepine-10,11-epoxide is a representative example of biologically relevant activation, whereas many contaminants undergo conjugation or sequestration, which ultimately reduces toxicity.

Phase II detoxification involves conjugation reactions mediated mainly by glutathione S-transferases (GSTs) and glycosyltransferases (**Figure 1**). GST activity has been reported to increase between 1.5- and 3-fold under contaminant exposure, indicating the activation of detoxification pathways (Hasanuzzaman et al., 2020; Dimaano and Iwakami, 2021). The quantitative ranges reported throughout this section were derived from representative experimental studies included in the literature survey (**Supplementary Tables 1**-**4**) and are intended to illustrate the variability of plant physiological responses rather than define universal thresholds. Their magnitude depends on contaminant chemistry, plant species, tissue type, exposure duration, and experimental conditions. However, this response is limited by the availability of glutathione. Under stress conditions, glutathione levels can decrease by 20–50%, reflecting the competition between detoxification and antioxidant defense processes (Hasanuzzaman et al., 2020). This depletion represents a central metabolic bottleneck that limits detoxification capacity and increases susceptibility to oxidative imbalance. Importantly, no universal threshold exists to define the transition from adaptive detoxification to oxidative imbalance. However, this transition appears to be strongly context-dependent, varying according to contaminant identity, exposure duration, contaminant mixtures, nutrient availability, and species-specific antioxidant capacity. In general, moderate glutathione depletion may be compensated for through antioxidant reprogramming and enzymatic upregulation, whereas prolonged or severe depletion increasingly constrains both detoxification efficiency and ROS scavenging, favoring oxidative imbalance and physiological impairment (López-Climent et al., 2013; Hasanuzzaman et al., 2020).

Although the three-phase detoxification model provides a useful conceptual framework, increasing evidence indicates that these phases frequently overlap and interact with redox regulation, primary metabolism, and stress signaling. Consequently, detoxification should be viewed as part of an integrated physiological network rather than as an isolated metabolic pathway. The relative contribution of each detoxification phase also varies considerably among contaminant classes, and remains insufficiently characterized for many recently recognized emerging contaminants.

Phase III detoxification involves sequestration into vacuoles, binding to cell walls, and transport via ATP-binding cassette (ABC) transporters (**Figure 1**). Although these processes reduce cytosolic toxicity, they do not eliminate contaminants from plant systems, leading to internal accumulation (Li et al., 2023). Importantly, these phases operate as an integrated metabolic network influenced by redox status, energy availability, and environmental conditions, rather than as strictly sequential processes. Despite their protective roles, sequestration mechanisms should not be interpreted as unlimited buffering systems. Vacuolar compartmentalization and ABC-mediated transport require continuous energy input and may be constrained under prolonged or high-intensity exposure. Evidence increasingly suggests that transporter saturation can occur when the contaminant influx exceeds the sequestration capacity, thereby increasing the proportion of bioactive compounds retained in metabolically sensitive compartments (Asare et al., 2022; Vitelli et al., 2024). These constraints may partially explain why detoxification efficiency frequently declines during chronic exposure, despite the initial activation of defense pathways.

Detoxification limitations become particularly evident under contaminant-mixture scenarios, where co-exposure may reduce metabolic efficiency by approximately 20–30% compared to single-compound exposure (Li et al., 2023). This reduction likely results from multiple interacting mechanisms, including substrate competition among CYP450 isoforms, competition for glutathione pools required for Phase II conjugation, and increased energetic demands associated with the simultaneous activation of detoxification and antioxidant pathways (Yang et al., 2026). Consequently, co-exposure scenarios may generate a disproportionately greater physiological burden than predicted from single-contaminant responses alone, further constraining detoxification performance under chronic exposure conditions. The mechanistic constraints and transformation outcomes are summarized in **Table 2**.

**Table 2:** Mechanistic constraints, species-dependent variability, and transformation outcomes associated with plant detoxification pathways under exposure to emerging contaminants.

Detoxification phase	Enzymatic system	Contaminant class	Quantitative response	Species-specific variability	Limitation/bottleneck	Transformation outcome	Key mechanistic insight	Reference
Phase I/transformation	Cytochrome P450-linked xenobiotic metabolism	Pharmaceuticals: carbamazepine + ibuprofen	CBZ uptake associated with stress symptoms and hormone-modulated defense responses	*Basella alba* shows tissue-level physiological responses after pharmaceutical uptake	Transformation may not eliminate biological activity	Persistent or bioactive metabolites; toxification risk	Phase I metabolism may increase, rather than reduce, toxicity when reactive intermediates are generated.	(Zhang et al., 2021)
Phase I/transformation	Antibiotic metabolism and stress-response networks	Fluoroquinolones: enrofloxacin	Multiomics evidence of hormetic and detrimental effects depending on exposure intensity	Rice responses depend on metabolic pathway reprogramming	Transformation and stress response may be dose-dependent	Hormesis at lower exposure; metabolic disruption at higher exposure	Metabolic outcome depends on contaminant dose and physiological context rather than detoxification alone.	(Chen et al., 2025b)
Phase I–II/antibiotic stress response	H_2_O_2_-scavenging enzymes; CAT/APX-centered detoxification	Antibiotics: amoxicillin, erythromycin, ciprofloxacin	Antibiotic exposure activates oxidative-stress and antioxidant pathways in *Lemna minor*	Aquatic macrophytes rely strongly on H_2_O_2_-scavenging systems	Detoxification depends on antioxidant buffering capacity	Redox-mediated tolerance or oxidative injury	Detoxification efficiency is tightly coupled to cellular redox homeostasis.	(Gomes et al., 2022c)
Phase II/conjugation and redox buffering	GST, GR, GSH/GSSG, AsA–GSH cycle	Pharmaceuticals: acetaminophen, carbamazepine, metformin	Pharmaceutical exposure alters proteomic and oxidative-stress responses in lettuce	Root and leaf responses differ in antioxidant and stress-defense profiles	Competition between detoxification and antioxidant GSH demand	Redox imbalance when GSH buffering is insufficient	Glutathione availability represents a central metabolic bottleneck linking detoxification and oxidative stress.	(Leitão et al., 2021a)
Phase II/stress mitigation	Hormonal and detoxification regulation	Oxytetracycline	24-epibrassinolide regulates oxytetracycline phytotoxicity and detoxification	Response depends on hormone-mediated defense activation	Detoxification efficiency depends on hormonal status	Reduced phytotoxicity through regulated detoxification	Hormonal signaling modulates detoxification capacity and contaminant tolerance.	(Chen et al., 2025c)
Integrated detoxification/antioxidant co-activation	SOD, CAT, APX, GST and redox signaling	Nanoplastics: PS nanoplastics	*Lemna minor* accumulates nanoplastics and activates hormonal and antioxidant tolerance mechanisms	Hyperaccumulator-like behavior reported in *L. minor*	Antioxidant activation may be insufficient at higher internal burden	Accumulation with tolerance or oxidative stress	Antioxidant activation delays but does not necessarily prevent physiological impairment under increasing contaminant burden.	(Arikan et al., 2022)
Integrated detoxification under combined stress	ROS scavenging, hormone modulation, AsA–GSH cycle	PS + PMMA nanoplastics under arsenic stress	Combined nanoplastics alter hormone content, chlorophyll fluorescence and ROS-scavenging capacity	*Lemna minor* responses depend on individual vs combined nanoplastic exposure	Co-exposure amplifies redox and hormonal burden	Altered photochemistry and ROS detoxification	Co-exposure amplifies physiological complexity through simultaneous activation of detoxification and stress signaling pathways.	(Ozfidan-Konakci et al., 2023)
Integrated nanomaterial response	Protein phosphorylation; antioxidant and hormonal networks	ZnO nanoparticles + nanoplastics	Co-exposure modulates protein phosphorylation and physiological responses in barley	Barley shows pathway-level regulation under combined nanoparticle stress	Nanomaterial mixtures impose signaling and energetic constraints	Reprogrammed stress physiology and detoxification coupling	Nanomaterial mixtures trigger coordinated signaling responses rather than independent effects.	(Guo et al., 2023)
Nanomaterial-specific oxidative detoxification	Nitro-oxidative signaling and antioxidant defense	ZnO nanoparticles	ZnO nanoparticles induce nitro-oxidative signaling in *Brassica* species	*B. napus* and *B. juncea* differ in tolerance-related root responses	Particle-specific ROS generation and metal-ion release may overlap	Oxidative signaling, stress adaptation, or toxicity	Nanoparticle toxicity arises from the interaction between particle effects and metal-ion release.	(Molnár et al., 2020a)
Physical/particulate stress	No enzymatic degradation; ROS and auxin disruption	Polystyrene nanoplastics	PSNPs disrupt redox homeostasis and antioxidant defenses in black mustard	Root and leaf responses differ under nanoplastic exposure	No true enzymatic degradation pathway; particulate persistence	Oxidative stress and impaired antioxidant defense	Nanoplastics are managed predominantly by sequestration and stress tolerance rather than true detoxification.	(Arikan et al., 2022)
Physical/particulate + pesticide interaction	ROS, enzymatic stress responses, contaminant vectoring	Microplastics + glyphosate	Combined exposure affects plant performance and stress physiology	*Salvinia cucullata* responses depend on combined particulate–herbicide exposure	Co-contaminant vectoring and oxidative burden	Altered toxicity relative to single exposure	Microplastics can modify pesticide bioavailability and physiological responses by acting as contaminant carriers.	(Yu et al., 2021)
Mixture/pharmaceutical wastewater	Multi-contaminant oxidative stress response	Pharmaceutical wastewater mixture	Wastewater exposure induces oxidative-stress responses in *Spirodela polyrhiza*	Aquatic macrophyte response reflects mixture composition and exposure intensity	Mixture complexity limits attribution to single detoxification pathway	Partial tolerance or redox injury under complex exposure	Mixture composition is often a stronger determinant of detoxification than the concentration of individual contaminants.	(Parveen et al., 2023)
Mixture/nanoplastic + pesticide	MAPK, detoxification genes, oxidative metabolism	PS nanoplastics + quinclorac	Combined exposure affects enzymology, physiology and transcriptome in rice	Rice responses involve roots and shoots with pathway-level reprogramming	CYP/GST competition and oxidative overload are plausible bottlenecks	Non-additive physiological and transcriptomic effects	Competition for detoxification pathways contributes to synergistic or antagonistic mixture effects.	(Lu et al., 2023)
Mixture/nanoplastic + organic pollutant	Metabolomic and detoxification networks	PS nanoplastics + dibutyl phthalate	Co-exposure alters metabolomics and phytotoxicity mechanisms in dandelion	Root cortex accumulation and metabolic responses vary with co-exposure	Combined chemical and particulate stress alters detoxification demand	Metabolic reprogramming and toxicity amplification	Co-occurring contaminants substantially increase metabolic costs associated with detoxification.	(Li et al., 2023)
Mixture/nanoplastic + PFAS	ROS-mediated stress interaction	PS microplastics + PFOA	Combined exposure affects oxidative-stress responses in *Chlorella sorokiniana*	Algal responses reflect interaction between particulate and PFAS stressors	ROS amplification under co-exposure	Enhanced oxidative burden relative to single stressors	Mixture toxicity frequently results from integrated disturbances in uptake, redox homeostasis, and detoxification rather than additive effects alone.	(Zhao et al., 2023)

Quantitative responses summarize the representative ranges reported under controlled experimental exposure scenarios. Detoxification phases frequently overlap *in vivo* and are strongly influenced by contaminant chemistry, exposure duration, species identity, and the presence of co-occurring stressors. Mixture effects represent deviations from single compound responses under co-exposure conditions.

Despite significant advances in understanding plant detoxification pathways, several important knowledge gaps remain. Most mechanistic evidence derives from controlled experiments involving individual contaminants, whereas detoxification under environmentally realistic exposure scenarios likely involves simultaneous competition among multiple xenobiotics for transporters, conjugation enzymes, reducing equivalents, and sequestration mechanisms. Future research integrating metabolomics, isotope tracing, and systems biology approaches will be essential to quantify detoxification fluxes, identify transformation products, and determine the physiological costs associated with long-term contaminant exposure.

### 4.2 Detoxification efficiency across contaminant classes

Detoxification efficiency varies widely across contaminant classes. Pharmaceuticals with moderate hydrophobicity (log K_ow_ 2–4) are typically removed with efficiencies between 40% and 80%, whereas persistent compounds, such as carbamazepine, can exhibit removal efficiencies below 40%, depending on the plant species (Li et al., 2023; Zhang et al., 2023). Importantly, detoxification efficiency may differ substantially among plant species exposed to the same contaminant, reinforcing that uptake capacity, tissue redistribution, antioxidant buffering, and enzymatic activity collectively contribute to metabolic performance.

In contrast, herbicides and pesticides are generally detoxified more efficiently, with removal rates exceeding 70–90% in some crop species, reflecting the evolutionary adaptation of plant detoxification systems and the strong induction of CYP and GST enzymes (Dimaano and Iwakami, 2021). However, even highly efficient detoxification may not necessarily indicate complete protection, as metabolic transformation can occasionally produce reactive intermediates or persistent metabolites with biological activity equal to or greater than that of the parent compound.

The use of nanomaterials introduces additional complexity. Exposure to nanoparticles, such as ZnO and AgNPs, has been associated with increased detoxification and antioxidant enzyme activities of up to 2–4-fold, indicating the simultaneous activation of detoxification and stress response pathways (Li et al., 2024b). Nevertheless, this co-activation imposes substantial metabolic costs, as antioxidant defense and detoxification pathways compete for reducing equivalents and energy resources. Under prolonged exposure, this trade-off may limit the overall detoxification performance and increase the physiological burden.

Micro- and nanoplastics differ fundamentally from other contaminants because they are not degradable by enzymes. Their interaction with plants is dominated by physical sequestration processes, with no evidence of metabolic degradation in plants. Additionally, these particles can act as vectors for co-contaminants, thereby altering their bioavailability (Wang et al., 2023). Consequently, plant responses to particulate contaminants frequently reflect the combined burden of physical stress, oxidative imbalance, and contaminant vectoring, rather than detoxification in the classical metabolic sense. Although these broad patterns are consistently reported, considerable overlap exists among contaminant classes owing to differences in contaminant chemistry, plant metabolism, and environmental conditions. Consequently, detoxification efficiency should be interpreted as a continuum rather than as a contaminant class-specific characteristic.

### 4.3 Detoxification under mixture exposure: functional constraints and competition

Under realistic environmental conditions, plants are exposed to mixtures of contaminants that significantly alter detoxification dynamics. Co-exposure can modify removal efficiencies by approximately ±20–30% compared to single-compound exposure, depending on the contaminant interactions and plant species (Li et al., 2023). Mixture exposure may result in antagonistic, additive, or synergistic interactions, creating detoxification responses that are frequently difficult to predict from single contaminant studies alone.

These interactions arise through multiple physiological mechanisms that vary according to contaminant identity. For example, nanoplastics may increase the bioavailability and tissue accumulation of co-occurring pesticides or hydrophobic organic contaminants by acting as carrier particles, whereas pharmaceuticals sharing common CYP-mediated metabolic pathways may compete for the same detoxification machinery, thereby reducing metabolic efficiency. Likewise, PFAS may influence membrane-associated transport processes and thereby modify the uptake and intracellular distribution of co-occurring contaminants, while combined exposure to nanoplastics and pesticides has been associated with non-additive physiological and transcriptomic responses (Lu et al., 2023). Collectively, these examples illustrate that mixture toxicity frequently emerges from interactions affecting contaminant uptake, intracellular transport, detoxification capacity, and redox homeostasis, rather than from simple concentration additivity.

A central mechanism underlying these effects is competition for enzymatic systems, such as CYPs and GSTs, which exhibit broad substrate specificity. Although this allows plants to metabolize diverse contaminants, it also reduces detoxification efficiency when multiple substrates are simultaneously present (Dimaano and Iwakami, 2021). Glutathione availability represents an additional bottleneck, as detoxification and antioxidant defenses compete for the same reducing pool. Under stress conditions, GSH depletion of approximately 20–50% has been reported, potentially constraining conjugation capacity and favoring the accumulation of partially transformed compounds (Hasanuzzaman et al., 2020). Therefore, detoxification constraints under mixture exposure are unlikely to emerge from a single biochemical limitation. Instead, reduced efficiency appears to reflect an integrated interaction among enzymatic competition, redox imbalance, transporter saturation, and the energetic costs associated with antioxidant activation, membrane transport, and cellular repair. Under chronic exposure, these combined demands may progressively shift detoxification from an adaptive defense mechanism to a substantial metabolic burden, thereby amplifying physiological impairment beyond expectations derived from individual contaminants alone.

### 4.4 Integration with redox regulation and physiological trade-offs

Detoxification is closely associated with redox regulation. Phase I reactions mediated by CYP enzymes involve electron transfer processes that generate ROS, directly linking detoxification and oxidative stress (Stading et al., 2020). Consequently, contaminant metabolism may paradoxically intensify the oxidative burden, particularly when ROS production exceeds the antioxidant buffering capacity.

Glutathione occupies a central position in this interaction because it functions simultaneously as a substrate for Phase II detoxification and as a major antioxidant involved in ROS scavenging and maintenance of cellular redox homeostasis. Increased allocation of GSH toward conjugation reactions may reduce its availability for antioxidant defense, creating a functional trade-off between detoxification and oxidative protection (Hasanuzzaman et al., 2020). Although no universal threshold currently exists to define when this balance collapses, reductions of approximately 20–50% in glutathione pools have been associated with impaired antioxidant performance and increased oxidative vulnerability under stress conditions. Under prolonged or high-intensity exposure, this competition for reducing equivalents may progressively favor oxidative imbalance, lipid peroxidation, and metabolic dysfunction.

These interactions indicate that detoxification should not be interpreted as a cost-free, protective process. Instead, increasing detoxification activity frequently imposes substantial physiological trade-offs, as reflected by the simultaneous induction of detoxification enzymes and oxidative stress biomarkers. The energetic demand associated with xenobiotic metabolism, antioxidant activation, membrane transport, and cellular repair may progressively redirect resources away from primary metabolism and growth, contributing to reductions in photosynthetic performance, biomass accumulation, and reproductive investment under continuous exposure to EEC. In this context, detoxification efficiency may ultimately become constrained not only by enzymatic capacity but also by the availability of metabolic energy and the reducing power.

Environmental conditions further modulate these interactions between species. Nutrient availability, temperature, light intensity, and co-occurring abiotic stressors can strongly influence both detoxification performance and redox balance, frequently altering contaminant toxicity (Gomes, 2024b). For example, resource limitations may intensify physiological trade-offs by reducing the energetic capacity required to simultaneously sustain both antioxidant and detoxification systems.

Despite its central role in plant responses to EECs, detoxification remains quantitatively undercharacterized. Most studies still rely on indirect indicators, including antioxidant enzymes, metabolite accumulation, and physiological biomarkers, whereas direct measurements of detoxification fluxes, metabolic costs, and transformation kinetics remain comparatively rare. This limitation constrains the predictive understanding of contaminant fate and reinforces the need for integrative approaches that combine metabolomics, isotope tracing, enzymatic kinetics, and systems-level physiology.

## 5 Oxidative stress and antioxidant responses

Oxidative stress has emerged as a central and unifying feature of plant responses to EECs; however, its mechanistic interpretation remains frequently oversimplified. A synthesis of experimental studies published since 2019 (**Table 3** and S3) revealed that redox perturbation is not merely a recurrent symptom of toxicity but rather a multi-layered physiological interface integrating contaminant perception, metabolic disruption, detoxification, and stress signaling. Although oxidative stress is one of the most consistently reported responses to EEC exposure, its biological significance remains highly context-dependent. Similar ROS profiles may reflect adaptive signaling associated with stress acclimation or progressive oxidative damage, depending on contaminant identity, exposure intensity, tissue specificity, developmental stage, and antioxidant capacity. Consequently, ROS should be interpreted as a dynamic regulatory process rather than as a universal indicator of toxicity.

**Table 3:** Approximate representation of contaminant classes and dominant oxidative mechanisms reported in experimental studies evaluating oxidative stress responses of plants exposed to emerging environmental contaminants (2019–present).

Contaminant class	Approximate representation in retrieved studies	Dominant ROS-generating mechanisms	Main oxidative biomarkers reported	Typical physiological outcomes	Key mechanistic insight
Nanomaterials/nanoplastics	~174	Surface-mediated redox reactions, disruption of chloroplastic and mitochondrial electron transport chains, membrane oxidative reactions, RBOH/NADPH oxidase activation, nanoparticle dissolution and ion release	SOD, CAT, APX, POD, ROS, H_2_O_2_, MDA/TBARS, antioxidant metabolites	Strong oxidative stress, membrane damage, photochemical impairment, metabolic perturbation	Strong evidence consistently supports direct ROS generation and oxidative disruption, although most studies rely on acute laboratory exposures.
Metals/metalloids*	~49	Redox cycling, mitochondrial and chloroplast dysfunction, Fenton-like reactions, disruption of redox-active proteins	ROS, H_2_O_2_, MDA, SOD, CAT, POD, APX	Severe oxidative damage, metabolic dysfunction, impaired growth	Well-established mechanistic framework used as a reference for interpreting oxidative responses to EECs.
Microplastics/macroplastic-derived particles	~36	Membrane perturbation, chloroplast structural damage, mechanical stress, auxin disruption and ROS signaling, carrier effects for co-contaminants	ROS, MDA, H_2_O_2_, SOD, CAT, APX	Photosynthetic impairment, oxidative imbalance, altered hormonal homeostasis	Oxidative stress is predominantly associated with physical particle interactions and contaminant vectoring rather than direct chemical toxicity.
Pharmaceuticals	~13	Interference with respiratory, chloroplastic and redox metabolism, mitochondrial dysfunction, ROS accumulation during xenobiotic metabolism	H_2_O_2_, CAT, APX, GST, MDA	Metabolic imbalance, oxidative stress, growth inhibition	Current evidence suggests that oxidative stress arises mainly as a consequence of xenobiotic metabolism, although the database remains comparatively limited.
Pesticides/herbicides	~11	Target-site inhibition of photosynthesis, disruption of electron transport chains, ROS amplification through metabolic inhibition	SOD, CAT, POD, APX, MDA	Photochemical stress, oxidative damage, reduced growth	ROS generation is closely linked to disruption of photosynthetic electron transport, but responses vary according to herbicide mode of action.
PFAS and industrial contaminants	~8	Membrane interactions, oxidative imbalance, mitochondrial dysfunction	ROS, antioxidant enzymes, MDA	Tissue-specific stress responses, altered metabolism	Available evidence remains limited, preventing generalization of oxidative mechanisms across PFAS classes.
Other emerging contaminants	~8	Variable mechanisms depending on contaminant chemistry	ROS, antioxidant enzymes, oxidative biomarkers	Diverse physiological responses	Current knowledge is fragmented, highlighting the need for mechanistic studies across a broader diversity of contaminant classes.

* Metal-based stress responses included primarily as mechanistic comparators because of their established oxidative pathways and relevance for understanding ROS generation in EEC-exposed plants. ** Table derived from literature screening performed using the Elicit platform (2019–present), including only primary experimental studies reporting oxidative stress biomarkers in plants exposed to EECs. Review papers, editorials, and non-experimental studies were excluded. Approximate representation reflects the number of retrieved studies meeting inclusion criteria and is intended to illustrate research emphasis rather than provide bibliometric quantification. Full study information, including plant species, contaminant identity, biomarkers evaluated, and experimental conditions, is provided in **Supplementary Table 3**.

A quantitative analysis of the compiled literature (**Table 3**) indicates that oxidative responses have been reported in the vast majority of studies, with hydrogen peroxide (H_2_O_2_), lipid peroxidation (MDA/TBARS), and antioxidant enzyme activities (SOD, CAT, and APX) forming the dominant response framework. Reactive oxygen species originate from multiple intracellular compartments, including chloroplasts, mitochondria, peroxisomes, and plasma membrane respiratory burst oxidase homologs (RBOHs/NADPH oxidases), emphasizing that oxidative stress reflects an interconnected signaling network rather than isolated ROS production. In particular, RBOH-mediated ROS generation plays a key role in stress amplification and signal propagation, often acting synergistically with calcium signaling pathways to coordinate systemic responses to environmental perturbations. The quantitative response patterns summarized throughout this section are derived from representative experimental studies included in the literature survey (**Table 3** and S3) and are intended to illustrate the variability of oxidative responses rather than define universal physiological thresholds. Their magnitude varies considerably according to contaminant chemistry, plant species, tissue type, exposure duration, and analytical methodology.

However, the compiled literature also revealed a substantial representation imbalance, with nanomaterials and plastic-derived contaminants accounting for a disproportionately large fraction of available experimental studies, whereas pharmaceuticals and pesticides remain comparatively underrepresented (**Table 3**). This imbalance has likely influenced the prevailing conceptual models of oxidative stress, which are frequently based on acute or particle-driven oxidative perturbations rather than the chronic, metabolism-mediated stress that is more characteristic of dissolved contaminants. Importantly, oxidative stress responses are not uniform across contaminant classes, plant species and tissues. Instead, they depend strongly on (i) the physicochemical properties of the contaminant, (ii) the exposure route, and (iii) organ-specific physiology. Root tissues consistently display higher contaminant accumulation and an earlier onset of oxidative imbalance, whereas leaves frequently exhibit a delayed yet stronger perturbation of photosynthetic metabolism. This tissue-specific pattern may also explain why APX exhibits greater sensitivity in leaves than in roots. As chloroplasts are major ROS-generating sites during photosynthetic electron transport, APX plays a particularly important role in maintaining redox homeostasis within photosynthetically active tissues. Consequently, moderate disturbances in chloroplastic metabolism often trigger earlier or proportionally stronger modulation of APX activity relative to CAT, especially under contaminant exposure, which affects PSII function or electron transport stability. Mitochondrial ROS generation may also contribute substantially under non-light conditions, including nighttime metabolism, when respiratory electron transport becomes the dominant source of oxidative signaling. This spatial and temporal decoupling highlights that oxidative stress in plants exposed to EECs should be interpreted as a compartmentalized and dynamic process, rather than a bulk cellular response.

An important but insufficiently resolved aspect of this research is the temporal hierarchy of oxidative stress biomarkers following contaminant exposure. Available evidence suggests that molecular and biochemical responses frequently precede whole-organism physiological impairment, although the precise timing remains highly species-, tissue-, and contaminant-dependent. Rapid responses often involve ROS accumulation and early antioxidant activation (e.g., SOD and CAT), whereas declines in photochemical performance (e.g., Fv/Fm) and visible growth impairment generally emerge later during sustained stress. Gene expression responses may occur even earlier, but temporal comparability across studies remains limited owing to substantial variations in exposure duration, sampling frequency, contaminant classes, and analytical endpoints. Consequently, although a generalized lag-time framework remains premature, current evidence supports the interpretation that oxidative biomarkers operate hierarchically, with early molecular perturbations progressively propagating toward physiological dysfunction under persistent exposures. Despite these emerging patterns, important uncertainties remain regarding the temporal transition from ROS-mediated signaling to irreversible oxidative injury, particularly under chronic exposure to contaminant mixtures. Most currently available evidence derives from short-term experiments involving single contaminants, limiting our ability to predict oxidative responses under environmentally realistic conditions where multiple stressors interact simultaneously.

### 5.1 Oxidative stress as a quantitatively recurrent but mechanistically heterogeneous response

Exposure of plants to EECs frequently disrupts cellular redox homeostasis, leading to the accumulation of ROS, including superoxide radicals (O_2_•^-^), H_2_O_2_, and hydroxyl radicals (•OH). However, evidence synthesized from recent primary studies indicates that although ROS accumulation is nearly ubiquitous, its magnitude, origin, and functional consequences vary substantially across experimental systems (**Table 4**). Across contaminant classes, quantitative responses show consistent trends: increases in H_2_O_2_ and lipid peroxidation are widely reported, with MDA levels frequently rising between ~20% and >100% depending on exposure conditions, and H_2_O_2_ often increasing by 1.5- to 5-fold in moderate-to-high exposure scenarios (**Table 4**). Although no universal threshold currently exists to define irreversible oxidative damage based on MDA accumulation, increases exceeding ~100% are frequently associated with severe membrane disruption and impaired photosynthetic performance under experimental conditions. Nevertheless, their biological significance remains strongly influenced by contaminant identity, plant species, antioxidant buffering capacity, and exposure duration. Importantly, ROS signaling rarely operates independently of other signaling pathways. Crosstalk among chloroplasts, mitochondria, peroxisomes, and plasma membrane ROS-generating systems is frequently amplified by calcium (Ca²^+^)-dependent signaling pathways, which act synergistically to coordinate systemic stress responses and transcriptional reprogramming upon contaminant exposure.

**Table 4:** Organ-resolved oxidative and antioxidant responses have been reported in primary studies evaluating plant exposure to emerging environmental contaminants (2019–present).

Contaminant class	Specific contaminant(s)	Exposure concentration	Species	Organ/tissue	Main exposure route	H_2_O_2_ response	MDA/TBARS response	Antioxidant response	Mechanistic insight	Reference
Nanoplastics	Polystyrene nanoplastics (PS-NPs)	100 and 200 mg L^-^¹	*Lemna minor*	Whole plant/fronds	Aqueous, predominantly root-contact	Increased at high dose; ~25% at 200 mg L^-^¹	TBARS +17% at 200 mg L^-^¹	SOD increased 3-fold (100 mg L^-^¹) and 4.6-fold (200 mg L^-^¹)	Mild exposure activated antioxidant defense; higher exposure exceeded detoxification capacity and induced oxidative damage	(Arikan et al., 2022)
Nanomaterials/nanoplastics	Engineered nanoparticles and polystyrene nanoplastics	Dose range not clearly extractable from summary	*Lactuca sativa*	Roots	Root uptake	Increased, not quantified	MDA +124.6%	Not specified in abstract-level extraction	Severe oxidative damage in roots linked to particle-induced metabolic disruption	(Li et al., 2024b)
Microplastics	Microplastics (polystyrene)	Not clearly specified in extracted summary	*Tetrastigma hemsleyanum*	Leaves/aerial tissues	Root uptake with systemic translocation	Not clearly specified	MDA +45.2%	CAT increased; POD increased	Oxidative stress was associated with collapse of photosynthetic-energy metabolism and activation of flavonoid-centered defense	(Xie et al., 2026)
Pharmaceuticals	Carbamazepine	Hydroponic exposure; concentration range not recoverable in current extraction	*Lactuca sativa*	Leaves and roots	Root uptake	Increased in both organs	Increased in both organs	Leaves: SOD, CAT, GPOD, APX increased; roots: CAT, GPOD, GR increased	Roots were the main detoxification interface, but leaves showed stronger oxidative impairment	(Leitão et al., 2021a)
Pharmaceuticals	Acetaminophen	Hydroponic exposure; concentration range not fully recoverable in current extraction	*Lactuca sativa*	Leaves and roots	Root uptake	Increased, more pronounced in leaves	Increased, more pronounced in leaves	CAT and SOD increased; GR activity and GSH content decreased; APX less important than CAT/GPOD for H_2_O_2_ removal	Oxidative stress was accompanied by a coordinated but non-redundant antioxidant response, with CAT playing a dominant H_2_O_2_-scavenging role	(Leitão et al., 2021b)
PFAS	PFOS	5 and 50 μg L^-^¹, 10 d	*Lactuca sativa*	Roots and leaves	Root uptake	Oxidative stress increased, not quantified in extracted summary	Not quantified in extracted summary	APX, POD, CAT and GPX involved	Roots accumulated much higher PFOS burdens than leaves and acted as the major detoxification compartment	(Li et al., 2020b)
PFAS	PFOS	5 and 50 μg L^-^¹, 10 d	*Lactuca sativa*	Roots vs leaves	Root uptake	Not quantified	Not quantified	Antioxidant defenses differed by organ	Roots accumulated ~150.9 and 1445.6 ng g^-^¹ DW; leaves accumulated ~8.8 and 82.5 ng g^-^¹ DW, highlighting strong organ asymmetry in contaminant load and oxidative buffering	(Li et al., 2020b)
Nanoplastics + metalloid	PS-NPs and PMMA-NPs + arsenate	PS 100 mg L^-^¹; PMMA 100 mg L^-^¹; As 100 μM	*Lemna minor*	Whole plant	Aqueous, predominantly root-contact	Not reported quantitatively	Lipid peroxidation altered; not quantitatively resolved in extraction	Individual PS or PMMA activated SOD, POX, GST, GPX and improved AsA/DHA and GSH/GSSG status; combined PS+PMMA was less effective	Mixture effects were non-additive and altered antioxidant organization rather than simply amplifying stress	(Ozfidan-Konakci et al., 2023)
Antibiotics	Amoxicillin, erythromycin, ciprofloxacin (with enzyme-inhibitor assays)	Up to 1.05 mg L^-^¹ depending on compound/treatment	*Lemna minor*	Whole plant	Aqueous, root uptake	Increased when scavenging capacity was inhibited	MDA increased under inhibitor treatments; decreased with AMX alone	APX, CAT and SOD central to tolerance	The study mechanistically demonstrated that H_2_O_2_-scavenging enzymes are not merely markers, but determinants of antibiotic tolerance and phytoremediation performance	(Gomes et al., 2020)
Pesticide	Chlorpyrifos	50, 100 and 150 μg dm^-^³, 21 d	*Elodea canadensis*, *Eleocharis acicularis*, *Mentha aquatica*	Leaves and roots	Aqueous exposure	Increased, not quantified	Increased lipid peroxidation, not quantified	GPX and GST altered; strongest responses at 150 μg dm^-^³	Antioxidant responses were strongly species- and tissue-dependent, indicating that aquatic macrophytes do not share a single oxidative strategy	(Sobiecka et al., 2022)
Nanomaterials	ZnO nanoparticles or ZnSO_4_	250–2000 mg L^-^¹	*Phaseolus vulgaris*	Leaves/whole seedlings	Soil and foliar applications	Not quantified	Increased at high concentrations	Antioxidant activity increased at low concentrations; not enzyme-resolved in extracted summary	Clear hormetic pattern: low doses stimulated antioxidant metabolism, whereas high doses promoted lipid peroxidation and stress	(Salehi et al., 2021)
Metal comparator	Cd	0, 0.25, 0.50 and 1 μM	*Phaseolus vulgaris*	Roots and leaves	Root uptake	Increased, not quantified	Increased, not quantified	Roots: SOD, APX and GPX increased at 0.25 μM but declined at higher Cd; leaves: CAT increased at 0.5 and 1 μM	One of the clearest demonstrations that antioxidant responses are both dose-dependent and organ-specific	(Gutiérrez-Martínez et al., 2020)
Nanomaterials	ZnO nanoparticles	300 and 2000 mg L^-^¹	*Hordeum vulgare*	Roots and shoots	Root uptake	Increased, not quantified	Increased	GR increased in roots and shoots; GST increased ~1.2- and 2.5-fold in roots; CAT increased ~1.19- and 1.56-fold in roots	Barley showed coordinated activation of glutathione-dependent defense and CAT under ZnO stress	(Voloshina et al., 2022)
Metal oxide nanoparticles	Nano-CdO and micro-CdO	Dose range present in study but not fully recoverable in current extraction	*Hordeum vulgare*	Roots and shoots	Root uptake	Increased, not quantified	Increased lipid peroxidation	SOD, CAT, GR and GST affected; root enzyme inhibition at high dose; glutathione and ascorbate increased in some tissues	CdO triggered a mixed phenotype of antioxidant activation and enzymatic inhibition, especially in roots	(Azarin et al., 2022)
Inorganic contaminant	Perchlorate	1 ppm	*Pistia stratiotes*, *Eichhornia crassipes*, *Salvinia molesta*, *Spirodela polyrhiza*	Roots and shoots	Aquatic exposure	Not quantified	Not quantified	CAT markedly elevated in shoots and roots of *Pistia*; APX increased in *Pistia* shoots, *Eichhornia* roots and *Salvinia* shoots; GPX changed little	A strong example of species- and organ-resolved antioxidant deployment across aquatic macrophytes	(Vivek, 2025)
Nanoplastics + pharmaceutical	Polar PE-NPs or non-polar PP-NPs + ketoprofen	Nanoplastics 100 nm; ketoprofen concentration not clearly extractable from current summary	*Brassica rapa*	Roots and aerial tissues	Root uptake with differential translocation	Increased	Increased	SOD and CAT upregulated	Non-polar PP nanoparticles induced stronger oxidative stress than polar PE, emphasizing the importance of particle chemistry in mixture toxicity	(Lu et al., 2025)
Microplastics + PFAS	PS microplastics + PFOA	PS 10 mg L^-^¹; PFOA 0.05, 0.5 and 5 mg L^-^¹, 96 h	*Chlorella sorokiniana*	Whole cells	Aquatic exposure	Increased; co-exposure aggravated oxidative stress	Not reported quantitatively in extraction	Antioxidant system activated, but enzyme-level details not resolved in abstract extraction	Co-exposure intensified oxidative stress beyond single-compound treatments	(Zhao et al., 2023)
Micro(nano)plastics + metal	PS micro(nano)plastics + Cd	Split-root system; concentration not fully recoverable from extraction	Parsley	Exposed root half vs uncontaminated root half	Split-root, localized root exposure	Increased at contaminated side	Increased at contaminated side	Non-enzymatic antioxidants involved, especially AsA, non-flavonoid polyphenols and α-pinene	Growth inhibition and oxidative damage were spatially restricted to the contaminated root compartment, showing that oxidative injury may remain localized under heterogeneous exposure	(Chen et al., 2025a)

Nanomaterials and nanoplastics typically induce rapid and high-intensity ROS production, which is driven by surface redox activity, membrane destabilization, and interference with the electron transport chain (Voloshina et al., 2022; Li et al., 2024a; Mallin et al., 2025). Recent evidence indicates that cobalt nanoparticles can penetrate chloroplast compartments, inducing thylakoid disorganization, plastoglobuli accumulation, and severe oxidative bursts associated with both nanoparticle surface reactivity and intracellular metal ion release (Mallin et al., 2025). In contrast, pharmaceuticals and pesticides more commonly generate ROS indirectly through target-specific metabolic disruption, such as the inhibition of photosynthetic electron flow or perturbation of mitochondrial respiration (Gomes et al., 2020; Leitão et al., 2021b, Leitão et al., 2021a). PFAS and other persistent contaminants exhibit another pattern characterized by chronic, low-intensity oxidative stress coupled with strong root accumulation and long-term metabolic imbalances (Li et al., 2020b).

These differences are also reflected in tissue-specific responses. Quantitative analysis of the compiled dataset (**Supplementary Table 3**) showed that root-dominated oxidative responses predominated in studies involving dissolved contaminants, particularly pharmaceuticals and PFAS, where uptake occurred primarily through the rhizosphere. Conversely, leaf oxidative responses were more common under particle-associated stress, especially when contaminants interfered with photosynthetic metabolism or were efficiently translocated to aerial tissues. Moreover, several studies demonstrated nonlinear dose–response relationships characterized by hormesis (Agathokleous et al., 2022). Low contaminant concentrations often stimulate antioxidant defenses without causing detectable damage, whereas higher concentrations result in the breakdown of redox homeostasis and accumulation of oxidative damage (Gutiérrez-Martínez et al., 2020; Salehi et al., 2021). This transition reflects the saturation of the antioxidant capacity and a shift from ROS signaling to ROS toxicity. Importantly, ROS accumulation should not be interpreted solely as an indicator of damage. Instead, it represents a central signaling node that links contaminant exposure to downstream physiological processes. Moderate ROS levels can trigger the transcriptional activation of antioxidant enzymes, detoxification pathways, and stress-responsive genes, whereas excessive ROS levels lead to lipid peroxidation, protein oxidation, and metabolic collapse (Molnár et al., 2020b; Soares et al., 2021; Voloshina et al., 2022).

Another key insight emerging from the quantitative synthesis is that oxidative stress responses are strongly influenced by exposure configuration, particularly under mixed-contaminant scenarios. Co-exposure studies (e.g., nanoplastics with metals or pharmaceuticals) have revealed that oxidative responses are frequently non-additive, with antagonistic or synergistic effects depending on contaminant interactions (Ozfidan-Konakci et al., 2023; Zhao et al., 2023; Chen et al., 2025a). These findings challenge the widespread assumption of independent contaminant effects and underscore the importance of interpreting oxidative stress within the framework of mixture toxicity. Most available studies, however, remain restricted to H_2_O_2_, MDA, SOD, and CAT, whereas major components of cellular redox buffering, including the ascorbate–glutathione cycle, thioredoxins, and non-enzymatic antioxidants, remain comparatively understudied. This methodological bias limits mechanistic interpretation and hinders identification of the key determinants of plant tolerance and phytoremediation capacity. Taken together, these findings demonstrate that oxidative stress under EEC exposure is best understood not as a single physiological endpoint but as a quantitatively recurrent yet mechanistically heterogeneous response shaped by contaminant chemistry, plant physiology, and environmental context. This conceptual shift is essential for accurately linking oxidative stress to downstream processes, including antioxidant defense, hormonal signaling, and phytoremediation efficiency.

Quantitative responses were extracted from primary experimental studies whenever numerical data were available. When exact values could not be reliably recovered from the original publication or abstract-level extraction, qualitative trends were indicated. A full literature synthesis and experimental metadata are provided in Supplementary Table.

### 5.2 Antioxidant systems: coordination, compartmentalization, and functional specificity

The activation of antioxidant systems is the primary mechanism by which plants maintain redox homeostasis under EEC exposure. However, quantitative evidence compiled from recent studies (**Table 4**) demonstrates that antioxidant responses are neither uniform nor redundant but instead comprise coordinated, compartment-specific networks whose organization depends on the origin, intensity, and persistence of ROS production.

This hierarchical organization is particularly evident within the core enzymatic triad of SOD, CAT, and APX. Across contaminant classes, SOD consistently exhibited the most pronounced and rapid induction, reflecting its upstream role in converting O_2_•^-^ to H_2_O_2_. Because superoxide radicals are highly reactive and poorly diffusible (Karpinska and Foyer, 2024), their rapid conversion is essential to prevent oxidative propagation and preserve cellular redox stability. Consequently, SOD frequently represents the first enzymatic response to contaminant-induced oxidative stress, often increasing several-fold before downstream antioxidant systems are fully activated. For example, exposure to nanoplastics in *Lemna minor* resulted in a 3–4.6-fold increase in SOD activity, accompanied by CAT increases of ~ 40–90% and MDA accumulation exceeding 60–120% at higher concentrations (Arikan et al., 2022). This pattern indicates that although SOD efficiently channels ROS into H_2_O_2_, the subsequent detoxification capacity becomes limited when ROS production exceeds enzymatic buffering, leading to lipid peroxidation.

In contrast, studies involving pharmaceuticals have revealed a distinct antioxidant configuration, in which CAT and peroxidase-mediated detoxification play a more prominent role (Gomes et al., 2022c). Exposure of *Lactuca sativa* to carbamazepine and acetaminophen induced significant increases in CAT activity (~30–80%) and peroxidase systems, whereas APX responses were comparatively variable and, in some cases, secondary (Leitão et al., 2021a, Leitão et al., 2021b). This suggests that under metabolically mediated stress, H_2_O_2_ detoxification relies predominantly on CAT- and peroxidase-mediated removal rather than chloroplast-centered APX regulation.

The importance of glutathione-dependent systems is particularly evident in chronic or chemically persistent exposure scenarios. In *L. sativa* exposed to PFOS, root tissues showed strong activation of CAT, APX, and GPX (typically ranging from 50% to 120% increases), accompanied by substantial contaminant accumulation, indicating that glutathione-mediated detoxification is central to maintaining redox balance under prolonged stress (Li et al., 2021). Notably, several studies have indicated that increases in enzymatic activity are not always sufficient to prevent oxidative damage when glutathione availability is limited. For instance, exposure of *L. sativa* to pharmaceutical compounds resulted in increases in CAT and peroxidase activities on the order of ~30%–80%, yet was accompanied by reductions in reduced glutathione (GSH) levels of approximately 20%–50% and concomitant increases in lipid peroxidation (MDA) exceeding 40%, indicating that enzymatic upregulation alone did not prevent oxidative damage (Leitão et al., 2021b). Similarly, nanoparticle exposure studies frequently report strong induction of SOD and CAT activities (often exceeding 100%–200% relative to control), while GSH depletion ranges from 25%–60%, accompanied by increases in H_2_O_2_ and MDA between 50%–150%, suggesting that redox buffering capacity, rather than enzyme activity *per se*, determines oxidative stress outcomes (Voloshina et al., 2022; Xie et al., 2026). In aquatic macrophytes exposed to mixed contaminants, shifts in GSH/GSSG ratios—often declining by 30%–70%—have been directly associated with the transition from adaptive antioxidant responses to oxidative injury, even under sustained enzymatic activation (Ozfidan-Konakci et al., 2023). Collectively, these findings indicate that antioxidant efficiency is governed less by enzyme induction alone than by the capacity to sustain intracellular reducing power and preserve redox buffering integrity under prolonged stress.

A major pattern emerging from the dataset is the strong divergence between root and leaf antioxidant responses, reflecting the differences in exposure pathways and metabolic roles. Roots, as the primary interface with dissolved contaminants, generally exhibit early and intense activation of antioxidant enzymes. For instance, PFOS exposure resulted in significantly higher accumulation in roots than in leaves, accompanied by marked increases in CAT, APX, and GPX activities (frequently >70–150% above control levels) (Li et al., 2021). Similar trends have been observed in metal and nanoparticle studies, where root tissues display rapid induction of SOD and CAT, even at relatively low concentrations, but also show higher susceptibility to enzymatic inhibition under severe stress (Gutiérrez-Martínez et al., 2020; Azarin et al., 2024). This suggests that while roots function as the first line of defense, their antioxidant systems may be more prone to collapse under sustained or high-intensity exposures.

Whereas roots function as the first biochemical barrier against contaminant entry, leaves exhibit delayed but functionally distinct antioxidant responses that are closely integrated with photosynthetic performance and energy metabolism. In these tissues, APX activity is often tightly regulated, particularly within chloroplasts, where it contributes to maintaining redox balance during photosynthetic electron transport. The heightened sensitivity of APX in leaves likely reflects its central role in chloroplast-associated H_2_O_2_ detoxification. As APX exhibits a high affinity for H_2_O_2_ and is tightly coupled to the ascorbate–glutathione cycle, its activity is particularly critical in photosynthetically active tissues, where ROS production is spatially concentrated around electron transport systems. This sensitivity is particularly relevant because chloroplastic APX isoforms localized in the stromal and thylakoid compartments are tightly associated with maintaining photosynthetic electron transport stability. Consequently, APX often responds more sensitively in leaves than other antioxidant enzymes, functioning as a fine regulator of chloroplastic redox homeostasis under contaminant-induced oxidative stress (Li, 2023; Yoshimura and Ishikawa, 2024). Disruptions in this system frequently result in a decline in photochemical efficiency (e.g., Fv/Fm), linking oxidative stress directly to physiological performance. In *L. minor* and *Myriophyllum aquaticum*, pharmaceutical stress resulted in increased H_2_O_2_ levels (~40–110%) and modulation of APX activity (typically 20–60% changes), which were directly associated with declines in Fv/Fm (reductions of ~10–25%) and photochemical performance (Castilho et al., 2025). Similarly, nanoparticle exposure in crop species has been shown to disrupt chloroplast redox balance, leading to altered APX activity and significant impairment of PSII efficiency (Fv/Fm decreases of ~15–30%) (Xu et al., 2024). These findings demonstrate that oxidative stress in leaves is not merely a consequence of ROS accumulation but reflects a breakdown in the tight regulation of chloroplastic redox homeostasis, directly linking the dynamics of antioxidants to photosynthetic performance. Together, this spatial decoupling indicates that antioxidant systems are not only tissue-specific but also functionally specialized, with roots primarily acting as detoxification barriers and leaves integrating redox signals with metabolic regulation.

Although enzymatic antioxidants dominate the literature, non-enzymatic components represent a critical, yet comparatively underexplored, dimension of redox regulation. The ascorbate–glutathione (AsA–GSH) cycle plays a central role in buffering H_2_O_2_ and sustaining antioxidant activity. In *L. minor* exposed to combined nanoplastics and arsenate, alterations in GSH/GSSG ratios (decline of approximately 30–70%) were closely associated with oxidative stress intensity and antioxidant enzyme modulation, indicating that redox buffering capacity directly influences tolerance outcomes (Ozfidan-Konakci et al., 2023). Additionally, exposure to microplastics in *Tetrastigma hemsleyanum* led to increased flavonoid accumulation (~20–80%) and elevated ROS levels, suggesting that secondary metabolites contribute to ROS scavenging and membrane stabilization under complex contaminant stress (Xie et al., 2024). Declines in glutathione despite enhanced enzymatic activity further indicate that antioxidant systems may become metabolically constrained during prolonged contaminant exposure (Leitão et al., 2021b).

The complexity of antioxidant regulation is further amplified in mixed-contaminant scenarios. Evidence indicates that co-exposure frequently produces non-additive responses, reflecting interactions that alter the bioavailability and metabolic processing of contaminants. In *L. minor*, combined exposure to nanoplastics and arsenate resulted in attenuated antioxidant enzyme responses (reductions of approximately 20–50% compared to single exposures), suggesting antagonistic interactions at the level of uptake or signaling (Ozfidan-Konakci et al., 2023). Conversely, co-exposure to microplastics and PFAS in microalgae intensified ROS production and antioxidant activation beyond additive expectations (ROS increases exceeding 120–200% relative to the control), indicating synergistic amplification of oxidative stress (Zhang et al., 2019). These contrasting patterns demonstrate that antioxidant systems respond not only to ROS levels but also to the interactions among co-occurring contaminants.

Taken together, the evidence supports a conceptual shift in the interpretation of antioxidant responses to EEC exposure. Rather than functioning as isolated stress markers, antioxidant systems operate as integrated and dynamic networks, the effectiveness of which depends on coordination across cellular compartments, the balance between detoxification and signaling, and the sustained metabolic support. Therefore, plant tolerance to contaminants emerges not simply from the activation of antioxidant defenses but from the capacity to maintain functional integration under complex and variable environmental conditions.

### 5.3 Contaminant-specific cellular targets and primary ROS-generation pathways

ROS production under EEC exposure is largely determined by the primary cellular targets of each contaminant class rather than representing a uniform stress response (**Table 5**). Current evidence indicates that chloroplasts, mitochondria, peroxisomes, plasma membrane NADPH oxidases (RBOHs), and the apoplastic interface constitute the principal ROS-generating sites, although their relative contributions depend on contaminant chemistry and exposure route (**Figure 1**).

**Table 5:** Contaminant-specific cellular targets, dominant ROS generation sites, and mechanistic pathways associated with oxidative stress in plants exposed to emerging environmental contaminants.

Contaminant class	Representative compounds	Primary cellular target	Dominant ROS-generation site	Mechanism	Predominant ROS	Evidence type	References
Pharmaceuticals	Carbamazepine, acetaminophen, antibiotics	Metabolic enzymes, respiratory metabolism, ETC	Mitochondria > chloroplasts (compound-dependent)	Disruption of respiration and photosynthetic metabolism	O_2_•^-^, H_2_O_2_	Biochemical, physiological	Leitão et al., 2021c, Leitão et al., 2021a; Gomes et al., 2022c
Herbicides/pesticides	Glyphosate, chlorpyrifos, PSII-targeting herbicides	Photosystems, metabolic pathways	Chloroplast	Electron transport disruption and photochemical imbalance	O_2_•^-^, H_2_O_2_	Physiological, biochemical	Soares et al., 2021; Sobiecka et al., 2022
Nanomaterials	ZnO, TiO_2_, Ag, CuO	Cell wall, membranes, organelles	Apoplast + chloroplast	Surface-mediated redox reactions + metabolic disruption	•OH, O_2_•^-^, H_2_O_2_	Physicochemical, biochemical	El-Zohri et al., 2021; Voloshina et al., 2022; Azarin et al., 2024
Micro-/nanoplastics	Polystyrene, polyethylene (PE)	Membranes, chloroplasts	Multiple compartments	Physical disruption, nutrient interference, carrier effects for co-contaminants	O_2_•^-^, H_2_O_2_, secondary •OH formation	Physiological, structural	Arikan et al., 2022; Xu et al., 2024; Lu et al., 2025
Metals/metalloids*	Cd, As, Cu	ETC, enzymes	Mitochondria + peroxisomes	Redox cycling and Fenton-like reactions	O_2_•^-^, H_2_O_2_, •OH	Biochemical	Molnár et al., 2020b

* Included as mechanistic comparators because of their established ROS generation pathways and relevance for interpreting oxidative mechanisms in EEC-exposed plants.

Contaminants that interfere with photosynthetic structures or electron transport preferentially induce ROS generation in chloroplasts. Herbicides targeting PSII represent the most direct case; however, pharmaceuticals and nanoplastics have also been shown to disrupt chloroplast ultrastructure and pigment organization, indirectly promoting electron leakage toward oxygen (Soares et al., 2021; Gomes et al., 2022c; Xu et al., 2024). This primarily results in superoxide formation at PSI, followed by dismutation to produce hydrogen peroxide. Thus, structural perturbations alone may destabilize photochemical redox homeostasis even in the absence of direct interactions with photosynthetic proteins.

Mitochondria are the second major source of ROS, particularly in the presence of contaminants that interfere with cellular energy metabolism. Pharmaceuticals, such as carbamazepine and acetaminophen, have been shown to alter respiratory electron transport, thereby promoting electron leakage from complexes I and III (Leitão et al., 2021c; Voloshina et al., 2022). Unlike chloroplastic ROS, mitochondrial ROS production is not light-dependent and can therefore sustain oxidative pressure under both light and dark conditions.

Peroxisomal ROS generation is largely associated with secondary metabolic imbalances, particularly through the photorespiration and lipid oxidation pathways. Under contaminant-induced disruption of carbon assimilation or stomatal conductance, an increased photorespiratory flux enhances the production of hydrogen peroxide via glycolate oxidase activity (Molnár et al., 2020b; Soares et al., 2021). Thus, peroxisomes primarily amplify oxidative stress arising from contaminant-induced metabolic dysfunction rather than serving as direct contaminant targets.

In contrast to these metabolically driven sources, plasma membrane-localized NADPH oxidases (RBOHs) generate ROS in a regulated, signal-mediated manner. Exposure to nanomaterials and microplastics has been shown to activates RBOH-dependent ROS production, suggesting that certain contaminants are perceived as external stimuli that trigger redox signaling cascades (Manna et al., 2021; Voloshina et al., 2022). This ROS production is typically rapid and localized and differs fundamentally from organelle-derived ROS associated with metabolic dysfunction.

Nanomaterials introduce an additional ROS generation pathway through abiotic surface-mediated reactions. Metal-based nanoparticles, such as ZnO, CuO, TiO_2_, and Ag, can catalyze redox reactions at the cell wall–membrane interface, producing ROS independently of cellular metabolism (El-Zohri et al., 2021; Azarin et al., 2024). These apoplastic ROS can subsequently propagate across membranes and trigger intracellular oxidative responses. This mechanism distinguishes nanomaterials from most other contaminant classes because ROS generation may precede metabolic disruption. Micro- and nanoplastics exhibit hybrid mechanisms of ROS induction, combining physical membrane perturbation, interference with nutrient transport, and carrier-mediated effects that enhance the uptake of co-contaminants (Arikan et al., 2022; Xu et al., 2024; Lu et al., 2025). Consequently, ROS production under plastic exposure is typically distributed across multiple compartments rather than being localized to a single site.

Importantly, the biological consequences of oxidative stress depend not only on the quantity of ROS but also on the identity, lifetime, diffusibility, and compartment of origin of ROS. H_2_O_2_, for example, functions as a comparatively stable signaling molecule capable of intracellular propagation, whereas •OH exhibit extremely high reactivity and are generally associated with localized cellular injury. The spatial organization of ROS generation is further shaped by the exposure route (Herb et al., 2021; Averill-Bates, 2024). Root uptake favors interactions in the apoplast, plasma membrane, and mitochondria, whereas foliar exposure directly targets chloroplast-rich tissues and epidermal compartments. Furthermore, inter-organelle signaling may amplify oxidative responses through ROS-induced ROS release, reinforcing the integration of chloroplasts, mitochondria, peroxisomes, and plasma membrane ROS sources into a dynamic redox network. This inter-organelle amplification is frequently coordinated by Ca²^+^-dependent signaling pathways, reinforcing the integration of redox signaling within cellular compartments.

Collectively, the available evidence supports a contaminant-centered framework in which ROS generation arises from specific interactions between contaminant properties and cellular targets. This mechanistic perspective complements the antioxidant dynamics described in Section 5.2 and provides the structural basis for understanding how oxidative stress propagates across cellular compartments (**Figure 1**).

### 5.4 From oxidative stress to metabolic reprogramming: adaptive and maladaptive trajectories

Oxidative stress induced by EECs extends beyond redox imbalance, triggering coordinated reprogramming of primary and secondary metabolism. Rather than representing isolated biochemical disturbances, ROS act as integrative signals that reshape carbon and nitrogen fluxes, redistribute reducing power, and reallocate cellular energy, ultimately determining whether plants maintain homeostasis or transition to metabolic dysfunction.

One of the earliest consequences of ROS accumulation is the reconfiguration of the central carbon metabolism. Perturbations in photosynthetic electron transport and mitochondrial respiration alter the balance between ATP production and reducing equivalents (NADH/NADPH), thereby forcing adjustments in glycolysis and tricarboxylic acid (TCA) cycle fluxes. Metabolomic studies in woody and crop species exposed to nanoplastics and nanoparticles have consistently reported significant shifts in key intermediates, such as citrate, malate, and succinate, with variations typically ranging from ±20% to ±70% relative to controls, indicating a dynamic redistribution of respiratory activity under oxidative pressure (Yu et al., 2021; Xu et al., 2024). These changes represent active metabolic adjustments that sustain energy supply and redox balance rather than merely reflecting cellular damage. Because chloroplast and mitochondrial metabolism are tightly interconnected, disturbances in photosynthetic electron transport frequently propagate to respiratory metabolism, reinforcing compensatory carbon reallocation under oxidative stress.

A tightly coupled response occurs in nitrogen metabolism, which is highly sensitive to redox changes. Alterations in nitrate reductase (NR), glutamine synthetase (GS), and glutamate synthase (GOGAT) activities have been consistently reported, reflecting the disruption of nitrogen assimilation pathways following contaminant exposure (Molnár et al., 2020b; Soares et al., 2021; Xu et al., 2024). Across contaminant classes, NR activity frequently declines by approximately 20–60%, particularly under herbicide and nanoparticle exposure, whereas GS and GOGAT exhibit biphasic responses, with moderate induction (~20–80%) under low to intermediate stress and inhibition (~15–50%) under higher exposure levels. The pronounced sensitivity of NR appears to be closely associated with ROS-mediated regulatory disruption. In addition to the direct oxidative modification of proteins, excessive ROS accumulation may interfere with nitrate signaling pathways and suppress the transcriptional and post-translational regulation of nitrogen-assimilation enzymes (Chu et al., 2021; Costa-Broseta et al., 2021). Stress-induced phosphorylation events and 14-3-3 protein interactions may further contribute to NR deactivation, reinforcing the sensitivity of nitrate assimilation to redox (Munier et al., 2021; García-Yagüe and Cuadrado, 2023). Thus, reduced NR activity reflects integrated redox signaling and metabolic reprogramming rather than simple enzymatic inhibition. As oxidative stress intensifies, dysregulation of the GS–GOGAT cycle promotes ammonium accumulation and disrupts amino acid homeostasis, further amplifying redox imbalance. Concomitantly, the accumulation of stress-associated amino acids, particularly proline (~30–150% increase), provides both osmoprotection and redox buffering, highlighting the integration of nitrogen metabolism in the mitigation of oxidative stress.

As oxidative stress intensifies, plants progressively redirect carbon from primary metabolism to secondary metabolite biosynthesis. This shift represents one of the most consistent metabolic signatures of contaminant exposure. Under moderate stress, enzymatic antioxidant systems dominate redox control; however, as ROS levels increase, the phenylpropanoid pathway is strongly activated, leading to the enhanced production of flavonoids, phenolic acids, anthocyanins, and related compounds. In *Populus* exposed to nanoplastics, flavonoid accumulation increased by approximately 50–120%, coinciding with a broader metabolic reorganization and partial stabilization of the redox status (Xu et al., 2024). Similar trends have been observed following exposure to PFAS, BPA, and metal-based contaminants, where increased phenolic content contributes to ROS scavenging and membrane protection (Li et al., 2021; Malea et al., 2022).

Lipid metabolism is another critical interface between oxidative stress and metabolic reprogramming. ROS-induced lipid peroxidation is frequently accompanied by adaptive membrane remodeling. Several studies have reported an increase in unsaturated fatty acids (~15–40%) under moderate contaminant exposure, suggesting an attempt to preserve membrane fluidity and function (Yusefi-Tanha et al., 2020; Voloshina et al., 2022). However, at higher stress intensities, lipid degradation dominates, leading to the accumulation of peroxidation products and loss of membrane integrity, reinforcing the transition from adaptive to damaging responses.

Metabolic reprogramming is coupled with detoxification pathway activation. ROS signaling also coordinates xenobiotic detoxification by inducing cytochrome P450 monooxygenases, glutathione S-transferases (GSTs), and conjugation pathways, thereby linking redox regulation directly to contaminant metabolism. Evidence from studies on pharmaceuticals and herbicides indicates that increased ROS levels frequently coincide with the enhanced activity of these detoxification enzymes, suggesting coordinated regulation between redox signaling and xenobiotic metabolism (Soares et al., 2021; Gomes et al., 2022c).

Importantly, these metabolic trajectories frequently follow hormetic patterns in which low or moderate contaminant exposure stimulates compensatory metabolic responses, whereas higher exposure progressively exceeds the buffering capacity and drives metabolic dysfunction. Under moderate exposure, plants exhibit adaptive reprogramming, characterized by metabolic flexibility, maintenance of energy balance, and effective integration of redox and metabolic pathways. In contrast, under severe or prolonged exposure, metabolic coordination is progressively disrupted. This maladaptive state is characterized by depletion of energy reserves, accumulation of toxic intermediates, collapse of redox buffering systems, and loss of metabolic control. The transition between these states marks a critical physiological threshold frequently associated with persistent impairment. Taken together, these findings demonstrate that oxidative stress operates as a central regulatory hub linking contaminant exposure to a large-scale metabolic reconfiguration. Plant tolerance therefore depends not only on ROS detoxification but also on the capacity to dynamically reorganize carbon and nitrogen metabolism in an energetically sustainable manner. This metabolic flexibility ultimately determines whether plants sustain contaminant tolerance and phytoremediation performance or undergo physiological collapse under prolonged exposure.

### 5.5 Mixture exposure and the limits of single-contaminant oxidative stress models

A critical limitation of the current knowledge on plant responses to EECs lies in the predominance of single-compound experimental designs. Although these studies have elucidated key oxidative stress mechanisms, they fail to capture the complexity of natural environments, where plants are simultaneously exposed to pharmaceuticals, pesticides, plastics, nanomaterials, and other co-occurring contaminants. Increasing evidence indicates that oxidative stress responses under mixture exposure are not additive but emerge from interactions that alter contaminant bioavailability, cellular targets, and metabolic processing.

One of the most consistent findings across mixture studies is the occurrence of nonlinear responses that deviate from classical dose–response expectations. In many cases, co-exposure leads to the synergistic amplification of oxidative stress, particularly when one contaminant enhances the uptake or persistence of another. For instance, nanoplastics have been shown to increase the bioavailability of sorbed pharmaceuticals and metals, resulting in elevated intracellular accumulation and ROS production exceeding 120–200% relative to single compound exposure (Zhao et al., 2023; Wang et al., 2024; Luo et al., 2025). Similar synergistic effects have been reported in microalgae and higher plants exposed to combined plastic–metal or plastic–pharmaceutical systems, where increased membrane permeability and carrier effects amplify oxidative and metabolic disruptions (Adeleye et al., 2024; Yang et al., 2024; Bryant et al., 2025). Conversely, antagonistic interactions may occur, particularly when one contaminant reduces the bioavailability of another through adsorption or sequestration. For example, graphene oxide has been shown to immobilize hydrophobic organic contaminants, thereby reducing their uptake and attenuating oxidative stress responses compared to single-compound treatments (Chakraborty et al., 2026). These contrasting responses demonstrate that mixture toxicity depends not only on intrinsic contaminant toxicity but also on physicochemical interactions occurring before cellular uptake.

Mixtures also modify the spatial organization of oxidative stress by simultaneously targeting multiple cellular compartments. This favors inter-organelle communication through ROS- and Ca²^+^-dependent signaling, amplifying oxidative responses beyond those expected from individual contaminants (Ravi et al., 2023; Shitaw et al., 2025). Such multi-compartment activation is particularly evident when nanomaterials are combined with metabolically active contaminants, coupling surface-mediated ROS production with intracellular metabolic disruption. Mixtures also accelerate the transition from adaptive to maladaptive metabolism by simultaneously activating multiple stress pathways with competing energetic demands. The resulting depletion of reducing equivalents and antioxidant reserves compromises detoxification efficiency and promotes earlier metabolic collapse than that observed under single-contaminant exposure (Zhang et al., 2023; Chen et al., 2024b).

A key implication of these findings is that traditional oxidative stress biomarkers may be insufficient to mechanistically resolve plant responses in mixed scenarios. Most studies continue to rely on endpoints such as ROS levels, lipid peroxidation, and antioxidant enzyme activity, which, although informative, do not elucidate the underlying interaction mechanisms driving non-additive effects. There is increasing recognition that future research must integrate toxicokinetic processes, subcellular localization of contaminants, and system-level metabolic responses to accurately characterize oxidative stress under realistic environmental conditions. Collectively, the available evidence demonstrates that oxidative stress under mixture exposure cannot be predicted from single-contaminant responses but emerges from complex interactions among contaminant behavior, cellular targets, and metabolic regulation. This highlights the need for integrative multi-contaminant frameworks capable of improving both mechanistic understanding and predictive assessments of plant performance under environmentally realistic conditions.

The metabolic transformations described in the previous section demonstrate that plant responses to emerging contaminants are not limited to detoxification processes but also involve extensive biochemical reconfiguration. These transformations are inherently coupled to redox metabolism, as Phase I and Phase II reactions frequently generate reactive intermediates and alter the cellular redox balance. In particular, enzymatic systems, such as cytochrome P450 monooxygenases and peroxidases, while central to contaminant transformation, contribute directly to the production of ROS (Stading et al., 2020), establishing a mechanistic link between detoxification pathways and oxidative stress signaling. Consequently, contaminant metabolism cannot be dissociated from redox dynamics, and the accumulation of ROS emerges as a systemic consequence of biochemical processing. This redox perturbation extends beyond metabolic compartments and propagates into regulatory networks, where ROS act as central signaling intermediates that control phytohormonal balance and physiological responses. Therefore, the metabolic and redox transformations described above must be interpreted as the upstream drivers of a broader regulatory cascade in which oxidative signals reprogram hormonal pathways and determine plant performance following contaminant exposure. These interconnected metabolic and redox responses provide the mechanistic basis for the redox-centered regulatory framework discussed in the following section, in which ROS signaling orchestrates phytohormonal regulation and whole-plant physiological responses.

## 6 Hormonal reprogramming under emerging contaminant exposure: organ-specific and mechanistic integration

Phytohormonal reprogramming under EEC exposure represents a direct extension of the redox perturbations described in Section 5, where ROS integrate contaminant perception with physiological and metabolic responses. Rather than acting independently, ROS and phytohormones form tightly interconnected regulatory networks that coordinate hormone biosynthesis, transport, perception, and downstream signaling.

Unlike antioxidant responses, which frequently converge across contaminant classes, phytohormonal responses are highly context-dependent. Their magnitude, direction, and physiological consequences vary substantially according to contaminant chemistry, exposure intensity, tissue sensitivity, and developmental stage. Consequently, plant hormonal responses to EECs exhibit strong organ specificity, nonlinear dose dependence, and mechanistic diversity rooted in the interplay among hormone biosynthesis, transport, compartmentalization, and signaling pathways.

Available evidence indicates that phytohormonal perturbations rarely result from direct contaminant–hormone interactions (**Supplementary Table 4**). Instead, they emerge from contaminant-induced redox reprogramming, metabolic disruption, and altered intracellular signaling. This framework helps explain why similar contaminants may trigger contrasting hormonal outcomes, depending on the plant species, exposure duration, and environmental context. In particular, root tissues often exhibit the earliest hormonal perturbations because they constitute the primary interface with the dissolved contaminants. Changes in auxin transport, abscisic acid (ABA) accumulation, jasmonate (JA) signaling, and gibberellin suppression have been repeatedly associated with contaminant-induced impairments in root elongation, meristem organization, and nutrient acquisition. In contrast, leaf tissues frequently integrate hormonal responses with photosynthetic regulation, stomatal behavior, and redox buffering, linking oxidative perturbations directly to carbon assimilation and energy metabolism.

Importantly, hormonal regulation during contaminant exposure frequently follows nonlinear trajectories. Moderate or transient oxidative perturbation may stimulate adaptive signaling responses that partially sustain growth, stress acclimation, and metabolic flexibility, whereas prolonged or high-intensity stress often shifts the hormonal balance toward defense-oriented programs characterized by elevated ABA and JA signaling, altered auxin homeostasis, reduced gibberellin activity, and growth inhibition. These responses frequently follow hormetic trajectories, in which low contaminant exposure activates compensatory physiological mechanisms, whereas higher exposure progressively exceeds the buffering capacity and promotes maladaptive outcomes.

**Figure 2:**
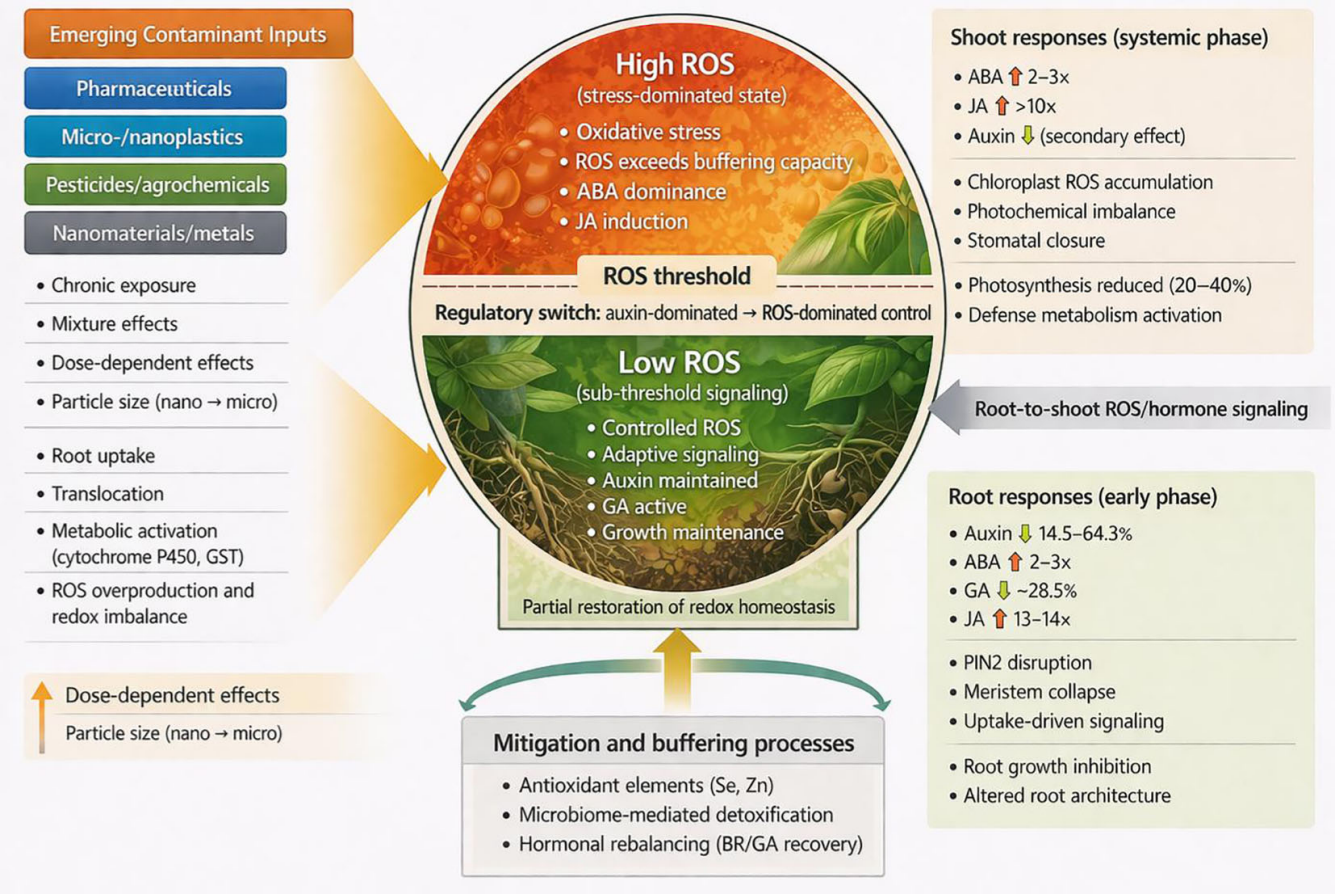
ROS-mediated phytohormonal reprogramming following exposure to emerging environmental contaminants (EECs). Pharmaceuticals, micro-/nanoplastics, pesticides, and nanomaterials disrupt plant redox homeostasis following uptake, translocation, and metabolic transformation, thereby promoting ROS accumulation and activation of stress-responsive signaling pathways. Depending on the identity of the contaminant, exposure intensity, and tissue sensitivity, ROS signaling may induce distinct hormonal reprogramming trajectories. Under moderate or subthreshold oxidative conditions, controlled ROS signaling contributes to adaptive responses, maintaining auxin homeostasis, photosynthetic performance, and growth regulation. In contrast, stronger or prolonged oxidative perturbations are frequently associated with increased abscisic acid (ABA) and jasmonate (JA) signaling, reduced gibberellin activity, altered auxin transport, impaired meristem maintenance, and growth inhibition. Root tissues frequently exhibit early hormonal disruption, including PIN-mediated auxin redistribution and altered ABA–JA balance, which may subsequently propagate to aerial tissues through vascular and systemic signaling pathways. Antioxidant buffering, micronutrient-mediated protection (e.g., Se and Zn), and microbiome-mediated modulation of contaminant uptake, antioxidant capacity, and hormonal homeostasis may partially restore redox homeostasis, thereby contributing to physiological recovery.

Together, the evidence summarized in **Figure 2** and **Supplementary Table 4** indicates that phytohormonal responses to EECs emerge from an integrated ROS–hormone regulatory network rather than isolated hormone-specific perturbations. This systems-level perspective provides the mechanistic basis for understanding how oxidative stress is translated into developmental, physiological, and metabolic outcomes under contaminant exposure. It also provides the conceptual framework for understanding how plant-associated microorganisms modulate contaminant responses through effects on hormone homeostasis, antioxidant capacity, and contaminant transformation, as discussed in the following section.

### 6.1 Root-centered hormonal reprogramming: uptake-driven signaling and early stress integration

Root tissues constitute the primary interface for the uptake of dissolved contaminants and therefore represent the earliest site of hormonal reprogramming in plants. Consistent with the patterns of contaminant accumulation and oxidative imbalance described previously for dissolved EECs, roots exhibit rapid and quantitatively pronounced hormonal shifts that reflect both direct chemical interactions and ROS-mediated signaling cascades.

Auxin is the most sensitive hormonal axis in this compartment. Across multiple contaminant classes, including pharmaceuticals and nanoplastics, root auxin levels decrease by approximately 14.5–64.3%, reflecting the disruption of auxin biosynthesis and, more critically, the impairment of polar auxin transport (Chen et al., 2025d; Ma et al., 2025; Yang et al., 2025). Mechanistically, this disruption is closely associated with ROS accumulation at the root apex, which alters the localization, stability, and membrane trafficking of PIN transporters. Increasing evidence suggests that ROS accumulation may promote PIN2 internalization and disrupt trafficking through endocytosis-associated processes (Zwiewka et al., 2019), thereby destabilizing the auxin gradients required to sustain root meristem organization. The resulting phenotype is characterized by reduced cell division, impaired lateral root formation, and decreased root elongation. Importantly, these effects are not merely symptomatic of toxicity; they define a structured signaling response in which ROS-mediated auxin redistribution actively redirects plant physiology away from growth and toward stress-adaptation. According to the logic shown in **Figure 2**, this constitutes the earliest transition from contaminant input to ROS-centered regulatory control.

Abscisic acid exhibits a markedly conserved response pattern in roots, with its concentration increasing by approximately two- to three-fold across a wide range of contaminants, including pharmaceuticals, nanomaterials, and persistent organic pollutants (Groffen et al., 2023; Ozfidan-Konakci et al., 2023; Xu, 2024). This increase reflects a tightly regulated feedback loop in which ROS stimulate ABA biosynthesis, while ABA enhances antioxidant defenses and modulates root hydraulic conductivity. However, deviations from this pattern provide valuable mechanistic insights. Under specific nanoplastic co-exposure scenarios, ABA levels decrease by approximately 16%, and this reduction is associated with increased co-contaminant toxicity, indicating that suppression of ABA signaling can directly compromise the plant’s capacity to buffer oxidative stress and sustain detoxification efficiency (Ozfidan-Konakci et al., 2023). Thus, ABA does not merely mark stress intensity; it determines whether the root systems remain functionally buffered or shift toward metabolic destabilization.

Gibberellins in root tissues generally exhibit negative regulation under contaminant stress, with reductions of approximately 28.5% reported under metalloid-associated exposure regimes (Xu et al., 2025). This decline contributes directly to growth inhibition and reflects broader suppression of anabolic metabolism. Conversely, when the redox balance is partially restored, such as in mitigation scenarios involving selenium or Zn-based treatments, gibberellin levels may increase by 66.2–160%, indicating the recovery of growth-associated signaling (Angelini et al., 2022; Asad et al., 2024). This bidirectional behavior reinforces the concept that ROS-dependent signaling progressively shifts hormonal regulation between growth-associated and defense-oriented programs, depending on stress intensity and buffering capacity.

Jasmonic acid is one of the most strongly induced hormones in root tissues, with increases ranging from approximately 13.1- to 14.7-fold under contaminant exposure (Li et al., 2022; Chen et al., 2025b). This pronounced upregulation reflects the activation of defense-related metabolic pathways and is tightly coupled to the intensity of ROS signaling. Because jasmonates originate from oxylipin metabolism, ROS-induced membrane lipid peroxidation may further reinforce JA biosynthesis, establishing a positive feedback loop between oxidative stress and defense signaling under contaminant exposure. In contrast, salicylic acid responses are more variable and frequently antagonistic to jasmonate signaling, indicating that ROS-mediated crosstalk determines the prioritization of defense pathways rather than inducing a uniform defense syndrome. Because JA- and SA-dependent signaling pathways frequently exhibit antagonistic interactions, prolonged activation of jasmonate-mediated responses under contaminant stress may also compromise resistance against certain biotrophic pathogens, reflecting a physiological trade-off between chemical stress tolerance and immune response. Ethylene further amplifies this regulatory structure by reinforcing ROS signaling and intensifying stress responses through interactions with ABA and jasmonate-associated pathways (Tripathi et al., 2024; Azhar et al., 2025).

Taken together, root hormonal responses are characterized by early onset, high sensitivity, and a strong association with contaminant uptake and redox imbalance. Therefore, roots function not only as contaminant entry points but also as primary signaling hubs from which systemic hormonal and physiological reprogramming is coordinated across plant tissues.

### 6.2 Aerial tissue responses: photosynthesis-driven signaling and delayed hormonal integration

In contrast to roots, aerial tissues experience the effects of contaminants primarily through systemic translocation and disruption of photosynthetic metabolism. As described in Section 5, oxidative stress in leaves is frequently delayed relative to that in roots but is more directly associated with chloroplast dysfunction and photochemical imbalances. This temporal and functional distinction is reflected in the hormonal responses of aerial tissues and corresponds to the shoot-centered outcome domain in **Figure 2**, where the ROS hub is linked to integrated stress phenotypes rather than to early transport disruption.

Auxin dynamics in leaves are less directly linked to transporter destabilization and are more strongly shaped by systemic signaling. Although reductions in auxin levels are commonly observed, they are generally less pronounced than in roots and often reflect downstream effects of root-derived signals rather than purely local biosynthetic inhibition. Under conditions of strong oxidative stress, particularly those affecting chloroplast integrity, auxin signaling may be further suppressed, contributing to reduced leaf expansion and an altered canopy architecture. Thus, whereas root auxin disruption is primarily associated with impaired transport and meristem organization, aerial auxin responses more frequently reflect systemic and metabolic reprogramming in the plant.

Abscisic acid plays a central role in coordinating aerial responses, particularly through regulation of stomatal conductance and water balance. The two- to three-fold increase in ABA observed across contaminant exposures frequently extends to leaves, where it contributes to stomatal closure, modulation of transpiration, and propagation of whole-plant stress signaling. However, in aerial tissues, ABA signaling becomes more tightly associated with chloroplast-derived ROS and guard cell regulation, linking oxidative imbalance directly to photosynthetic performance and gas exchange. In **Figure 2**, ABA occupies a dominant position in the high-ROS state, where stress signaling overrides growth-associated hormonal programs.

Jasmonic acid induction in leaves mirrors that observed in roots but is more directly associated with defense metabolism and secondary metabolite production. The strong upregulation of JA, often exceeding tenfold, is accompanied by enhanced synthesis of phenolics and flavonoids, linking hormonal signaling directly to the metabolic reprogramming described in Section 5. In photosynthetically active tissues, this response appears to be more closely coupled to chloroplast dysfunction and oxidative membrane perturbation, reinforcing the role of JA in coordinating secondary metabolism under stress. This coordination helps explain why contaminant-driven stress in aerial tissues is frequently accompanied by a marked reorganization of secondary metabolism.

Leaf tissues also exhibit a tighter association between hormonal signaling and photosynthetic performance than do roots. Disruptions in chloroplast redox balance, reflected by declines in photochemical efficiency, are frequently accompanied by coordinated changes in hormone levels, particularly ABA and JA. This integration highlights the role of chloroplast-derived ROS as signals that modulate hormonal pathways and reorganize metabolic priorities in plants. Accordingly, aerial tissues do not simply receive root-derived stress signals; instead, they integrate, amplify, and reprocess these signals through photochemical dysfunction and redox-sensitive hormonal control.

### 6.3 Concentration-, size-, and mechanism-dependent hormonal regulation

A defining feature of hormonal responses to EECs is their nonlinear behavior, which reflects the underlying dynamics of ROS production and signaling. Hormetic responses are widely observed, with low contaminant concentrations promoting growth and high concentrations inducing stress. For example, exposure to microcystins at 1 μg L^-^¹ stimulates growth through increased auxin and gibberellin levels, whereas concentrations ranging from 10 to 1000 μg L^-^¹ inhibit growth via elevated ABA levels and oxidative stress (Liang and Liu, 2020). Rather than reflecting a fixed threshold, this transition appears to emerge progressively as oxidative perturbation exceeds the buffering capacity of the antioxidant and hormonal regulatory systems.

Particle size introduces an additional and mechanistically decisive layer of complexity. Nanoplastics with diameters on the order of 20 nm induce strong oxidative stress and disrupt auxin transport through PIN2-mediated mechanisms, leading to root growth inhibition and meristem impairment (Ma et al., 2026). In contrast, microplastics of approximately 2 μm promote growth by approximately 11.87–23.47%, which is associated with increased auxin and gibberellin signaling (Zhang et al., 2024; Shao et al., 2026). These contrasting responses reflect a shift from intracellular oxidative stress toward organism-level signaling modulation, emphasizing that contaminant size influences not only uptake and localization but also the dominant regulatory mechanism governing hormonal outcomes.

Quantitative analyses further demonstrated that different mechanisms dominated under different exposure scenarios. Growth inhibition associated with larger particles is primarily driven by the disruption of auxin homeostasis, accounting for approximately 32.4% of the observed variation, whereas toxicity associated with smaller particles is more strongly linked to oxidative stress pathways, accounting for approximately 38.6% of the variation (Yang et al., 2025). These findings are particularly relevant because they move the discussion beyond descriptive toxicology and indicate that hormonal outcomes are shaped by shifts in mechanistic predominance across exposure contexts rather than by contaminant identity alone. In **Figure 2**, this concept is represented by the progressive transition between adaptive ROS signaling and defense-oriented hormonal reprogramming. Accordingly, the figure should be interpreted not as a static signaling network but as a conceptual model illustrating how hormonal regulation shifts according to the contaminant intensity, size-dependent uptake, and redox perturbation.

### 6.4 Integration of ROS–hormone networks with metabolic and detoxification processes

Hormonal reprogramming following EEC exposure is tightly coupled to metabolic reconfiguration and detoxification processes. As described in Section 5, ROS signaling drives large-scale changes in carbon and nitrogen metabolism, which are mediated in part by hormonal pathways. Accordingly, hormonal responses should not be interpreted as processes operating in parallel to metabolism; rather, they represent one of the principal regulatory outcomes of the same redox perturbations that govern detoxification, physiological stability, and stress acclimation.

The suppression of auxin and gibberellin signaling, combined with the activation of ABA and jasmonate pathways, reflects a coordinated shift from growth-oriented to defense-oriented metabolism. This transition is associated with increased allocation of resources toward antioxidant [production, secondary metabolism, and xenobiotic detoxification. Consequently, hormonal regulation plays an important role in modulating the expression and activity of detoxification pathways, including cytochrome P450 monooxygenases, glutathione S-transferases, and transporter systems involved in sequestration and compartmentalization of these compounds. Importantly, the effectiveness of these responses depends on the maintenance of cellular redox balance. Under sustained oxidative perturbation, excessive ROS accumulation may progressively compromise both hormonal coordination and detoxification performance, thereby reducing the capacity of plants to sustain integrated metabolic regulation. Conversely, interventions that partially restore hormonal balance, including brassinosteroids, auxin, selenium, and gibberellic acid, have been shown to mitigate contaminant toxicity by enhancing antioxidant defense and stabilizing metabolic function (Angelini et al., 2022; Zeeshan et al., 2024).

This recovery-oriented framework is conceptually illustrated in **Figure 2** through the mitigation module, which illustrates how antioxidant buffering, hormonal rebalancing, and metabolic stabilization may partially restore physiological homeostasis under contaminant stress. Although quantitative restoration thresholds remain insufficiently resolved, the mechanistic evidence synthesized in **Supplementary Table 4** supports the view that hormonal regulation is a critical determinant of whether plants maintain stress acclimation or transition toward metabolic dysfunction following prolonged contaminant exposure.

### 6.5 Synthesis: a ROS-centered, organ-resolved hormonal framework

The available evidence supports a conceptual framework in which ROS act as central integrators of hormonal crosstalk, linking contaminant exposure to the physiological responses of plants. This framework is inherently organ-specific, with roots functioning as primary sensing and signaling compartments, whereas aerial tissues integrate and amplify these signals through the photosynthetic and metabolic pathways. This organizational logic is depicted in **Figure 2**, where diverse contaminant classes converge on a central ROS signaling hub that modulates the downstream hormonal and physiological outputs. The magnitude and direction of hormonal responses are determined by the contaminant properties, including the dose and particle size, as well as the plant physiological context. These interactions generate complex and nonlinear outcomes that cannot be adequately captured by simplified stress-response models. Instead, plant responses to EECs should be interpreted as emergent properties of a dynamically regulated network in which ROS, hormones, metabolism, and detoxification processes are tightly interlinked.

Simultaneously, the broader evidence base compiled in **Supplementary Table 4** highlights important limitations in quantitative resolution, particularly for mitigation pathways and underrepresented contaminant classes. Several studies were retained primarily for mechanistic interpretation rather than quantitative hormonal resolution, emphasizing the current imbalance between mechanistic understanding and numerical standardization in the literature. This limitation identifies an important research priority: the need for future experimental designs capable of quantifying hormonal recovery dynamics and regulatory transitions with the same rigor currently applied to oxidative biomarkers, as discussed in Section 5. Overall, this integrated perspective provides a mechanistic basis for predicting plant responses under realistic environmental conditions and reinforces the importance of simultaneously considering the spatial, physiological, and quantitative dimensions of plant ecotoxicology.

## 7 Climate–contaminant interactions as determinants of plant physiological reprogramming and contaminant fate

Plant responses to EECs cannot be adequately understood under static environmental conditions, as physiological performance, redox homeostasis, and contaminant fate are intrinsically modulated by climatic drivers such as temperature, radiation, atmospheric CO_2_, and water availability. These factors do not merely act as external modifiers but fundamentally reshape the internal biochemical and physiological landscape of plants, thereby redefining both toxicity outcomes and phytoremediation pathways. The integration of recent evidence (**Table 6**) demonstrates that climate variables simultaneously regulate contaminant bioavailability, plant metabolic activity, and the mechanisms governing contaminant fate.

**Table 6:** Climate-driven modulation of plant physiological responses and contaminant fate in emerging contaminant exposure systems.

Climate factor	Contaminant(s)	Plant system	Key physiological response	Quantitative effect^1^	Fate pathway dominance^2^	Reference
Temperature (10–21 °C)	Atrazine; S-metolachlor	*Spirodela polyrhiza*	Photosynthetic sensitivity > growth	3–8% → 17% removal	Biotransformation	(Cruz et al., 2025b)
Temperature + UV	Antibiotics	*Salvinia*; *Myriophyllum*	ROS and pigment shifts	Up to 5-fold synergistic	Retention vs transformation	(Castilho et al., 2025)
Elevated CO_2_	Nanoplastics	*Hordeum vulgare*	Redox reprogramming	3.3× accumulation	Uptake	(Jian et al., 2023)
Drought	Microplastics; antibiotics	*Oryza sativa*	Metabolic disruption	Stronger combined stress	Reduced uptake	(Khan et al., 2024)
Temperature/light	Pesticides	Aquatic macrophytes	Enhanced remediation	38.86% increase	Combined pathways	(Cruz et al., 2025a)

¹ Quantitative values represent experimentally reported or meta-analytical outcomes.

² Dominant fate pathway refers to the predominant mechanism under given climatic conditions (uptake, biotransformation, retention). Additional supporting studies, including those lacking full mechanistic partitioning, are summarized in **Supplementary Table 5** (**Supplementary Materials**).

Temperature is a central regulator of contaminant–plant interactions because it controls enzymatic kinetics, membrane fluidity, contaminant transport and metabolic turnover. Experimental evidence indicates that lower temperatures intensify contaminant toxicity while constraining detoxification capacity. In *Spirodela polyrhiza*, exposure to atrazine and S-metolachlor resulted in greater photosynthetic impairment at 10 °C than at 15–21 °C, whereas S-metolachlor removal increased from 3–8% at lower temperatures to 17% at 21 °C (Cruz et al., 2025b). This pattern reflects physiological decoupling between toxicity and remediation. Reduced metabolic activity under cold conditions limits biotransformation pathways, including cytochrome P450-mediated oxidation and conjugation reactions, while simultaneously diminishing antioxidant buffering and membrane transport efficiency, thereby increasing oxidative damage and limiting the processing of contaminants.

Radiation further complicates this relationship by simultaneously acting as a degradative and stress-inducing factor. UV exposure enhances the abiotic photodegradation of contaminants while promoting ROS overproduction within plant tissues. Under combined temperature and UV stress, *Salvinia molesta* exhibited synergistic growth inhibition, reaching up to fivefold greater than additive expectations, whereas antagonistic interactions emerged at higher temperatures (Castilho et al., 2025). These findings indicate that radiation-induced ROS production interacts with temperature-dependent antioxidant systems, generating nonlinear physiological responses. Moreover, plant morphology modulates the outcome, with floating species favoring contaminant retention through shading and submerged or emergent species facilitating UV-driven transformation processes. Thus, radiation reshapes not only toxicity but also the dominant contaminant fate pathways.

Elevated atmospheric CO_2_ levels introduce an additional layer of complexity by modifying carbon metabolism, redox balance, and resource allocation. Under elevated CO_2_ (~800 µmol mol^-^¹), barley exposed to nanoplastics exhibited a 3.3-fold increase in root accumulation, along with reduced oxidative stress, characterized by decreased superoxide dismutase activity and increased catalase activity (Jian et al., 2023). This pattern suggests partial physiological decoupling between the contaminant burden and physiological impairment, likely mediated by enhanced carbon assimilation and improved antioxidant buffering. Accordingly, elevated atmospheric CO_2_ may partially enhance the capacity of plants to tolerate and accumulate contaminants without immediate physiological disruption, potentially altering long-term contaminant cycling and ecological risks.

Water limitation and osmotic stress represent critical axes of interaction. Drought conditions consistently amplify contaminant toxicity, as demonstrated in rice systems co-exposed to microplastics or antibiotics, where combined stress produces stronger reductions in growth, yield, and metabolic stability than individual stressors (Khan et al., 2024). These responses are largely mediated by ABA-driven signaling, reduced stomatal conductance, and impaired photosynthesis, which collectively constrain the energetic resources required for detoxification processes. At the same time, decreased transpiration limits contaminant uptake from the external environment while potentially increasing internal concentrations due to reduced dilution, thereby exacerbating toxicity. Consequently, under drought conditions, plants progressively transition from functional phytoremediation systems to stress-dominated physiological states with reduced remediation capacity.

A key emerging insight from the integrated analysis is that climate drivers determine not only the magnitude of plant responses but also the dominant pathways that govern contaminant fate. Elevated CO_2_ conditions tend to favor uptake and accumulation, whereas increased temperature and radiation frequently enhance the biotransformation processes. At the same time, plant structural traits modulate sorption and retention dynamics. Importantly, these interactions are frequently nonlinear and may generate additive, antagonistic, or synergistic outcomes, depending on climatic intensity, contaminant identity, and plant physiology. Consequently, contaminant fate should not be interpreted as a fixed property of plant systems but rather as an emergent outcome of interactions between environmental forcing and physiological states.

At the mechanistic level, climate-driven effects are mediated by ROS signaling networks and hormonal regulation, as discussed in Section 6. Climate stressors alter ROS production, shifting the balance between adaptive signaling and oxidative damage, which in turn influences hormonal pathways involving ABA, auxins, and jasmonates. Although quantitative hormonal data under combined climate–contaminant exposure remain limited, the evidence synthesized in **Supplementary Table 4** supports the interpretation that hormonal reprogramming is a central component of plant adaptation to complex environmental stresses. Rather than reflecting a fixed-threshold process, transitions between acclimation and physiological impairment appear to emerge progressively as oxidative perturbation exceeds the buffering capacity of redox and hormonal systems.

Despite these recent advances, substantial knowledge gaps remain. Few studies have simultaneously quantified climate variables, contaminant exposure, hormonal responses, and contaminant fate partitioning within a single experimental framework. Additionally, climate-driven shifts in toxicity are rarely expressed using standardized physiological thresholds (e.g., EC_10_ or EC_50_), limiting their comparability across studies. Addressing these limitations will require integrative experimental designs that combine environmental gradients, contaminant mixtures, and multilevel physiological and biochemical measurements.

In summary, climate factors function as primary regulators of plant–contaminant interactions, modulating physiological performance, redox balance, and contaminant fate pathways in a highly interconnected manner. Therefore, integrating climate dynamics into plant ecotoxicology is essential for improving predictions of contaminant behavior under future environmental conditions and designing effective nature-based remediation strategies.

## 8 Plant–microbiome interactions under emerging contaminant exposure

Plant responses to EECs cannot be fully understood in isolation from the plant-associated microbiome, which functions as an integral extension of the plant stress response system. Consequently, microbiome-mediated processes influence nearly every physiological stage discussed in the preceding sections, including contaminant uptake and internal exposure (Section 3), detoxification (Section 4), redox homeostasis (Section 5), and phytohormonal regulation (Section 6), thereby integrating plant stress responses across multiple biological scales. Increasing evidence supports the concept of the plant holobiont, in which plant performance under environmental stress emerges from the functional complementarity and metabolic coordination between the host and its rhizosphere-associated and endophytic microbial communities (Trivedi et al., 2020; Gomes et al., 2026). Within this framework, contaminant exposure frequently induces a “cry for help” response, whereby plants actively reshape their root exudation profiles to recruit beneficial microorganisms capable of mitigating chemical stress. These alterations frequently involve changes in the release of low-molecular-weight organic acids (e.g., malate and citrate), phenolic compounds, flavonoids, amino acids, and soluble sugars, which collectively regulate microbial recruitment, contaminant bioavailability, and rhizospheric metabolic function. Importantly, these exudate-mediated changes may promote extracellular contaminant transformation at the root interface, thereby reducing internal contaminant exposure and reshaping downstream ROS dynamics, hormonal regulation, and microbial community assembly (Rizaludin et al., 2021; Afridi et al., 2024).

The rhizosphere is a highly dynamic biochemical interface that regulates contaminant mobility, bioavailability, and transformation. Root exudates modulate microbial community assembly and metabolic activity, thereby influencing xenobiotic degradation and the speciation of contaminants. Rhizospheric microorganisms directly participate in contaminant transformation through oxygenases, reductases, hydrolases, and peroxidases, which collectively alter contaminant persistence, mobility, and toxicity (Wei et al., 2023b). In parallel, horizontal gene transfer mediated by catabolic plasmids accelerates microbial adaptation to chemically stressed environments, facilitating the rapid dissemination of degradation and resistance traits within these communities (Ijaz et al., 2025). By transforming contaminants at the root interface, these microorganisms reshape internal exposure dynamics and thereby influence the downstream detoxification, ROS, and hormonal responses described in the preceding sections. Under pharmaceutical and antibiotic exposure, these processes may additionally favor the enrichment of antimicrobial resistance determinants, reinforcing the need to interpret phytoremediation systems within the broader One Health context.

Plant growth-promoting microorganisms (PGPM), particularly rhizobacteria and endophytes, play a central role in enhancing plant tolerance to EECs. Mechanistically, these organisms promote stress mitigation through multiple complementary pathways, including phytohormone production, ACC (1-aminocyclopropane-1-carboxylate) deaminase activity, siderophore-mediated metal chelation, biofilm formation, and the upregulation of antioxidant defenses (Naing et al., 2021; Shahid et al., 2023). Beyond these classical PGPM functions, recent evidence from a pilot-scale hybrid constructed wetland demonstrated that antibiotic attenuation was accompanied by structured shifts in rhizosphere microbial communities across treatment compartments, highlighting that microbial community assembly itself contributes to contaminant attenuation and ecosystem functioning in nature-based treatment systems (Maranho et al., 2026) Recent evidence has demonstrated that microbiota-associated modulation of plant physiology can directly influence pharmaceutical phytoremediation efficiency and stress resilience. Gomes et al. (2026) demonstrated that microbial symbiosis in *Lemna minor* reduced internal metformin accumulation by regulating extracellular degradation pathways, thereby reducing the physiological burden through the coordinated modulation of oxidative stress, hormonal homeostasis, and photosynthetic performance. Axenic plants accumulated 2.1-fold more metformin and 1.7-fold more guanylurea, accompanied by elevated lipid peroxidation and stronger activation of cytochrome P450-dependent detoxification pathways, whereas non-axenic systems showed enhanced antioxidant activity and a lower physiological burden. These findings demonstrate that microbiome-mediated regulation extends beyond contaminant degradation, directly influencing oxidative stress, hormonal homeostasis, metabolic adjustment, and the physiological processes sustaining phytoremediation efficiency under pharmaceutical exposure. Collectively, these mechanisms reduce ethylene-mediated growth inhibition while modulating ABA-, auxin-, and jasmonate-dependent signaling, enhancing antioxidant capacity and maintaining redox homeostasis during contaminant exposure (Upadhyay, 2025; Das and Maji, 2026). Importantly, recent evidence has demonstrated that microbial consortia generally provide greater functional resilience than single-strain inoculants, particularly under complex contaminant mixtures, by expanding metabolic redundancy and enhancing xenobiotic transformation capacity (Santoyo et al., 2021; Kochi et al., 2023; Zhang et al., 2025).

Beyond direct metabolic interactions, the microbiome modulates plant stress physiology by fine-tuning ROS dynamics and phytohormonal signaling networks. These microbiome-mediated processes directly influence the ROS–hormone regulatory network described in Sections 5 and 6, reinforcing the concept that oxidative stress and hormonal reprogramming emerge from coordinated plant–microbe interactions rather than plant responses alone. Microbial metabolites can influence ABA-, JA-, and auxin-dependent pathways while simultaneously altering antioxidant activity and ROS accumulation patterns, effectively priming plants for enhanced tolerance during acute or chronic exposure (Singh et al., 2023; Yue et al., 2026). This interaction reinforces the concept that oxidative stress should not be interpreted exclusively as a plant-centered process, but rather as a system-level phenomenon extending across the plant–microbiome metabolic network. However, contaminant exposure also reshapes the microbiome structure and function. Pharmaceuticals, antibiotics, herbicides, nanomaterials, and microplastics can reduce microbial diversity, select for resistant taxa, and alter the abundance of functional genes associated with xenobiotic degradation, nutrient cycling, and antimicrobial resistance (Zhang et al., 2022; Ahmed et al., 2024; Chen et al., 2024a; Malinoski et al., 2026). These shifts may either enhance or impair phytoremediation efficiency, depending on the ecological stability and metabolic adaptability of the microbial community. Consequently, plant–microbiome interactions under EEC exposure should be interpreted as bidirectional and dynamically co-regulated.

Collectively, plant-associated microbiota should be viewed as integral components of the physiological framework governing contaminant uptake, detoxification, redox homeostasis, hormonal regulation, and phytoremediation efficiency. This interpretation is consistent with recent microbiome-informed frameworks proposing that rhizosphere communities regulate contaminant transformation, resistance selection, and ecosystem functioning in nature-based systems (Barros et al., 2026). By reshaping contaminant fate and internal exposure dynamics, microbial communities influence oxidative signaling, plant metabolism, and ecosystem resilience under realistic multi-stressor scenarios.

## 9 Translational applications in phytoremediation and environmental monitoring

The increasing mechanistic understanding of plant responses to EECs is progressively transforming phytoremediation from an empirical remediation strategy into a predictive, physiology-informed nature-based solution. Advances in redox biology, transport physiology, microbiome research, and multi-omics have demonstrated that phytoremediation efficiency depends not only on contaminant removal capacity but also on the ability of plants to maintain metabolic stability, regulate oxidative stress, and sustain physiological performance under chronic chemical exposure. Consequently, physiological knowledge is becoming an essential component in the design, monitoring, and optimization of plant-based remediation technologies. Physiological and biochemical biomarkers traditionally employed in plant ecotoxicology are increasingly being incorporated into environmental monitoring programs and phytoremediation management. Parameters such as chlorophyll fluorescence, ROS accumulation, antioxidant enzyme activity, phytohormonal modulation, detoxification metabolism, and redox biomarkers provide mechanistic insight into contaminant bioavailability and internal stress dynamics that cannot be obtained through physicochemical analyses alone (Oláh et al., 2021; Anjitha et al., 2023; Zaidi et al., 2024). Importantly, these responses frequently precede visible toxicity, enabling early detection of sublethal stress, physiological decline, or acclimation before reductions in remediation efficiency become evident.

Recent advances in transcriptomics, metabolomics, proteomics, and microbiome profiling have further expanded the translational potential of plant-based monitoring systems. Rather than functioning as isolated biomarkers, these approaches generate integrated molecular signatures capable of discriminating contaminant classes, exposure intensities, and stress persistence (Jain et al., 2024; Li et al., 2026). Combined with contaminant fate analyses and physiological phenotyping, these tools support the development of predictive monitoring frameworks capable of identifying early alterations in transport, detoxification, redox regulation, and microbiome-mediated stress buffering. In particular, microbiome-associated indicators are emerging as highly sensitive descriptors of environmental disturbance because contaminant exposure frequently reshapes rhizospheric community structure, metabolic functionality, and resistance-associated gene abundance (Aryal, 2024; Chukwuneme and Babalola, 2025).

These mechanistic advances are also driving the development of microbiome-assisted phytotechnologies designed to improve contaminant removal while maintaining plant physiological performance. Rather than relying exclusively on the intrinsic metabolic capacity of plants, recent phytoremediation strategies increasingly employ plant growth-promoting microorganisms, rhizospheric bioaugmentation, and engineered microbial consortia to enhance contaminant degradation, nutrient acquisition, antioxidant capacity, and stress resilience (Naing et al., 2021; Shahid et al., 2023). Recent evidence further demonstrates that microbial symbiosis can substantially modify contaminant fate and plant physiology. Gomes et al. (2026) showed that microbial symbiosis in *Lemna minor* reduced internal metformin accumulation through extracellular degradation pathways, resulting in lower oxidative stress, improved photosynthetic performance, and reduced physiological burden. These findings support the emerging concept of engineered phytobiomes, in which microbial inoculants are selected not only for their degradation potential but also for their capacity to regulate ROS dynamics, phytohormonal balance, and plant stress acclimation.

The incorporation of phytoremediation into engineered treatment systems has further expanded its operational applicability. Constructed wetlands and hybrid treatment systems integrate plant uptake, rhizospheric degradation, microbial metabolism, sediment interactions, and physicochemical filtration into multifunctional platforms for wastewater polishing and sludge treatment (Kochi et al., 2020; Maranho and Gomes, 2024). Emergent, floating, and submerged macrophytes provide complementary physiological niches that integrate plant uptake, microbial degradation, oxidative transformation, and contaminant sequestration across treatment compartments, thereby improving contaminant attenuation and ecological resilience (Maranho and Gomes, 2024). In parallel, bioaugmentation strategies employing tailored microbial consortia and emerging genetic and metabolic engineering approaches are contributing to increasingly sophisticated phytoremediation systems (“Phytoremediation 4.0”), in which ecological engineering, synthetic biology, and systems-based optimization are combined to maximize remediation performance (Thakur et al., 2025; Wentzell, 2025). Nevertheless, most engineered microbial consortia and synthetic biology approaches remain at the proof-of-concept stage, emphasizing the need for long-term field validation before widespread implementation.

An additional challenge for operational phytoremediation is the management of contaminant-enriched biomass following treatment. Although plants effectively attenuate contaminants through uptake, sequestration, transformation, or rhizospheric degradation, harvested biomass may retain contaminants or transformation products that require appropriate disposal. This issue is particularly relevant for pharmaceuticals, PFAS, pesticides, and metal-based contaminants characterized by persistence or incomplete degradation. Strategies including controlled composting, anaerobic digestion, bioenergy conversion, thermal treatment, and contaminant-specific disposal routes have been proposed (Kochi et al., 2020), although standardized guidelines remain limited and continue to represent an important barrier to large-scale implementation.

Despite these advances, environmental heterogeneity, seasonal variability, fluctuating contaminant loads, and multiple climate-related stressors continue to influence phytoremediation performance in nonlinear ways. Consequently, operational optimization increasingly depends on integrating physiological biomarkers, omics technologies, ecological engineering, and predictive computational tools into dynamic decision-support frameworks capable of adapting phytoremediation systems to changing environmental conditions.

From a One Health perspective, phytoremediation represents far more than an environmental cleanup strategy. By reducing contaminant loads and limiting contaminant transfer across soils, water, food webs, and ultimately human populations, plant-based remediation systems contribute simultaneously to ecosystem restoration, biodiversity conservation, and public health protection. These translational applications illustrate how advances in plant physiology can be converted into practical nature-based solutions addressing the growing challenges posed by emerging environmental contaminants.

## 10 Research gaps and future priorities

Despite recent advances, several critical knowledge gaps continue to hinder both the mechanistic understanding of plant responses to EECs and the translation of plant physiology into operational phytoremediation technologies. As discussed throughout this review, plant responses emerge from dynamic interactions among contaminant uptake, internal exposure, detoxification, redox regulation, metabolic reprogramming, phytohormonal signaling, and plant–microbiome interactions. However, these processes are still predominantly investigated in isolation, limiting the development of predictive frameworks capable of explaining plant performance under environmentally realistic conditions.

One of the most significant limitations is the predominance of experiments based on single contaminants under controlled laboratory conditions. In contrast, plants in natural ecosystems are chronically exposed to chemically complex contaminant mixtures interacting with multiple abiotic stressors, including drought, heat, salinity, ultraviolet radiation, and nutrient limitation. These combined stressors frequently generate nonlinear physiological responses that cannot be predicted from individual contaminant studies. Future research should therefore prioritize environmentally realistic exposure scenarios integrating contaminant mixtures with climate-related stressors to improve the ecological relevance of mechanistic investigations.

Another unresolved challenge is the limited understanding of interspecific variability in contaminant uptake, internal transport, detoxification capacity, and physiological tolerance. Current knowledge is heavily biased toward a relatively small number of model species, making it difficult to extrapolate mechanistic findings across different taxonomic groups, ecological strategies, and phytoremediation systems. Comparative studies involving terrestrial and aquatic plants, crop species, native vegetation, and wetland macrophytes are needed to identify conserved physiological mechanisms as well as species-specific adaptive strategies.

Progress is also constrained by the limited integration of biological organization across scales. Physiological, biochemical, transcriptomic, metabolomic, proteomic, microbiome, and contaminant fate studies are frequently conducted independently, despite describing interconnected processes. Future investigations should integrate these complementary approaches within unified experimental frameworks capable of linking contaminant transport, intracellular compartmentalization, oxidative signaling, metabolic adaptation, and whole-plant performance. Such integration will facilitate the identification of robust mechanistic biomarkers while improving predictive models for environmental monitoring and phytoremediation optimization.

The plant microbiome represents another major research frontier. Rather than being considered an external modifier of plant performance, rhizosphere and endophytic microbial communities should be investigated as integral components of contaminant response networks. Future studies should clarify how microbial communities regulate contaminant transformation, internal exposure dynamics, antioxidant capacity, phytohormonal homeostasis, and long-term stress resilience, particularly under realistic contaminant mixtures and field conditions. Understanding these interactions will be fundamental for the development of microbiome-assisted phytotechnologies and engineered phytobiomes capable of enhancing remediation efficiency while maintaining plant physiological stability.

Finally, translating mechanistic knowledge into practical environmental solutions remains a major challenge. Long-term field validation, standardized experimental protocols, predictive physiological models, and the integration of systems biology, artificial intelligence, and ecological engineering will be essential for improving the scalability and reliability of phytoremediation technologies. Ultimately, future progress will depend on shifting plant ecotoxicology from predominantly descriptive studies toward predictive, systems-based frameworks capable of integrating contaminant fate, plant physiology, microbiome functionality, and ecosystem processes across multiple biological scales.

## 11 Conclusions and future perspectives

Emerging environmental contaminants (EECs) impose complex and multidimensional physiological pressures that extend far beyond conventional toxicological paradigms centered solely on growth inhibition or oxidative damage. The evidence synthesized in this review demonstrates that plant responses to pharmaceuticals, pesticides, PFAS, nanomaterials, and microplastics emerge from the dynamic integration of contaminant uptake, internal exposure, detoxification pathways, redox regulation, phytohormonal signaling, and microbiome-mediated buffering. Rather than isolated stress responses, these processes constitute interconnected regulatory networks operating across molecular, cellular, organismal, and ecosystem scales.

A major conceptual advance emerging from recent research is the recognition that contaminant toxicity is fundamentally governed by internal exposure dynamics rather than external concentrations alone. Physicochemical properties, vascular transport, tissue compartmentalization, metabolic transformation, and plant–microbiome interactions collectively determine contaminant fate and ultimately shape physiological responses. Within this framework, oxidative stress should no longer be interpreted merely as a downstream symptom of toxicity but as a central regulatory hub linking xenobiotic perception to metabolic reprogramming, hormonal regulation, and stress acclimation. This perspective also explains why contaminant mixtures and interactions with climate-related stressors frequently produce nonlinear responses that cannot be predicted from single-contaminant experiments.

Another major advance is the recognition of the plant microbiome as an active determinant of contaminant tolerance and phytoremediation efficiency. Rhizospheric and endophytic microorganisms not only participate in contaminant transformation but also regulate internal exposure, antioxidant capacity, phytohormonal homeostasis, nutrient acquisition, and physiological resilience. Consequently, phytoremediation should be interpreted as a holobiont-mediated process in which plant physiology and microbial functionality operate as an integrated biological system.

From a translational perspective, the convergence of mechanistic plant physiology, microbiome engineering, and ecological treatment technologies is progressively transforming phytoremediation from an empirical remediation strategy into a predictive, physiology-informed nature-based solution. Constructed wetlands, microbiome-assisted phytotechnologies, and hybrid ecological systems illustrate how mechanistic understanding can be translated into practical environmental applications that simultaneously improve contaminant removal, ecosystem restoration, biodiversity conservation, and environmental resilience.

Ultimately, plants occupy a strategic interface linking environmental contamination, ecosystem functioning, and human health. Future progress will depend on replacing reductionist endpoint-based approaches with predictive, systems-oriented frameworks that integrate contaminant fate, plant physiology, microbiome functionality, and ecosystem processes across multiple biological scales. Such an integrated perspective places plant physiology at the center of sustainable environmental management and reinforces its fundamental role within the One Health framework.

## References

[B1] Abdelmoneim M. S. Hafez E. E. Dawood M. F. A. Hammad S. F. Ghazy M. A. (2024). Toxicity of bisphenol A and p -nitrophenol on tomato plants: Morpho-physiological, ionomic profile, and antioxidants/defense-related gene expression studies. Biomol. Concepts 15, 20220049. doi: 10.1515/bmc-2022-0049 38924751

[B2] Adeleye A. T. Bahar M. M. Megharaj M. Fang C. Rahman M. M. (2024). The unseen threat of the synergistic effects of microplastics and heavy metals in aquatic environments: A critical review. Curr. pollut. Rep. 10, 478–497. doi: 10.1007/s40726-024-00298-7 30311153

[B3] Afridi M. S. Kumar A. Javed M. A. Dubey A. de Medeiros F. H. V. Santoyo G. (2024). Harnessing root exudates for plant microbiome engineering and stress resistance in plants. Microbiol. Res. 279, 127564. doi: 10.1016/j.micres.2023.127564 38071833

[B4] Agathokleous E. Zhou B. Geng C. Xu J. Saitanis C. J. Feng Z. . (2022). Mechanisms of cerium-induced stress in plants: A meta-analysis. Sci. Total Environ. 852, 158352. doi: 10.1016/j.scitotenv.2022.158352 36063950

[B5] Ahmed A. A. M. Jui S. J. J. Sharma E. Ahmed M. H. Raj N. Bose A. (2024). An advanced deep learning predictive model for air quality index forecasting with remote satellite-derived hydro-climatological variables. Sci. Total Environ. 906, 167234. doi: 10.1016/j.scitotenv.2023.167234 37739083

[B6] Angelini J. Klassen R. Široká J. Novák O. Záruba K. Siegel J. . (2022). Silver nanoparticles alter microtubule arrangement, dynamics and stress phytohormone levels. Plants 11, 313. doi: 10.3390/plants11030313 35161294 PMC8838976

[B7] Anjitha K. S. Sarath N. G. Sameena P. P. Janeeshma E. Shackira A. M. Puthur J. T. (2023). Plant response to heavy metal stress toxicity: The role of metabolomics and other omics tools. Funct. Plant Biol. 50, 965–982. doi: 10.1071/FP23145 37995340

[B8] Arikan B. Alp F. N. Ozfidan-Konakci C. Yildiztugay E. Turan M. Cavusoglu H. (2022). The impacts of nanoplastic toxicity on the accumulation, hormonal regulation and tolerance mechanisms in a potential hyperaccumulator - Lemna minor L. J. Hazard. Mater. 440, 129692. doi: 10.1016/j.jhazmat.2022.129692 35963084

[B9] Aryal M. (2024). Rhizomicrobiome dynamics: A promising path towards environmental contaminant mitigation through bioremediation. J. Environ. Chem. Eng. 12, 112221. doi: 10.1016/j.jece.2024.112221 38826717

[B10] Asad M. Khan Z. Shah T. Shah M. A. Imran A. Rasool S. . (2024). Mitigation of microplastic toxicity in soybean by synthetic bacterial community and arbuscular mycorrhizal fungi interaction: Altering carbohydrate metabolism, hormonal transduction, and genes associated with lipid and protein metabolism. Plant Stress 14, 100631. doi: 10.1016/j.stress.2024.100631 38826717

[B11] Asare M. O. Száková J. Tlustoš P. (2022). The fate of secondary metabolites in plants growing on Cd-, As-, and Pb-contaminated soils—a comprehensive review. Environ. Sci. pollut. Res. 30, 11378–11398. doi: 10.1007/s11356-022-24776-x 36529801 PMC9760545

[B12] Avellan A. Yun J. Morais B. P. Clement E. T. Rodrigues S. M. Lowry G. V. (2021). Critical review: Role of inorganic nanoparticle properties on their foliar uptake and in planta translocation. Environ. Sci. Technol. 55, 13417–13431. doi: 10.1021/acs.est.1c00178 33988374

[B13] Averill-Bates D. (2024). Reactive oxygen species and cell signaling. Review. Biochim. Biophys. Acta Mol. Cell. Res. 1871, 119573. doi: 10.1016/j.bbamcr.2023.119573 37949302

[B14] Azarin K. Usatov A. Minkina T. Alliluev I. Duplii N. Mandzhieva S. . (2024). Impact nano- and micro-forms of CdO on barley growth and oxidative stress response. J. King Saud Univ. Sci. 36, 103493. doi: 10.1016/j.jksus.2024.103493 38826717

[B15] Azarin K. Usatov A. Minkina T. Plotnikov A. Kasyanova A. Fedorenko A. . (2022). Effects of ZnO nanoparticles and its bulk form on growth, antioxidant defense system and expression of oxidative stress related genes in Hordeum vulgare L. Chemosphere 287, 132167. doi: 10.1016/j.chemosphere.2021.132167 34509010

[B16] Azhar W. Salam A. Khan A. R. Ahmad I. Gan Y. (2025). Brassinosteroids alleviate ethylene-induced copper oxide nanoparticle toxicity and ultrastructural and stomatal damage in rice seedlings. Agriculture 15, 907. doi: 10.3390/agriculture15080907 30654563

[B17] Aziz S. Zaib S. Iqbal A. Chuadhry M. A. Shahzad S. Dhara B. . (2026). Emerging contaminants and their influence on plants: An in-depth review. Environ. Toxicol. Pharmacol. 121, 104872. doi: 10.1016/j.etap.2025.104872 41270919

[B18] Barros D. C. de Freitas L. H. K. Gomes M. P. (2026). Microbiome‐informed pathways linking nature‐based treatment systems to antimicrobial resistance outcomes. Environ. Microbiol. 28, e70358. doi: 10.1111/1462-2920.70358 42309504

[B19] Bigott Y. Khalaf D. M. Schröder P. Schröder P. M. Cruzeiro C. (2021). Uptake and translocation of pharmaceuticals in plants: Principles and data analysis. Handbook of Environmental Chemistry. 103, 103–140. doi: 10.1007/6982020622/SAVE-RESEARCH

[B20] Boahen E. Owusu L. Adjei-Anim S. O. (2025). A comprehensive review of emerging environmental contaminants of global concern. Discov. Environ. 3, 144. doi: 10.1007/s44274-025-00259-x 30311153

[B21] Bryant M. T. Rossi L. Mu R. Cao Z. Ma X. (2025). Synergistic effects of polystyrene nanoplastics and cadmium on the metabolic processes and their accumulation in hydroponically grown lettuce (Lactuca sativa). J. Agric. Food. Chem. 73, 16157–16164. doi: 10.1021/acs.jafc.5c03215 40551680 PMC12232387

[B22] Carter L. J. Harris E. Williams M. Ryan J. J. Kookana R. S. Boxall A. B. A. (2014). Fate and uptake of pharmaceuticals in soil–plant systems. J. Agric. Food. Chem. 62, 816–825. doi: 10.1021/jf404282y 24405013 PMC3908740

[B23] Castilho B. M. Simões L. C. Soares M. S. Carneiro D. N. M. Gomes M. P. (2025). Interactive effects of temperature and UV radiation on antibiotic uptake and degradation in a floating (Salvinia molesta) and a submerged/emergent (Myriophyllum aquaticum) macrophyte. J. Hazard. Mater. 496, 139366. doi: 10.1016/j.jhazmat.2025.139366 40749653

[B24] Chakraborty P. Paul N. Christudoss A. C. Mukherjee A. (2026). Graphene oxide nanomaterials reduce tetrabromobisphenol-A accumulation and attenuate phytotoxicity in Allium cepa. Plant Nano Biol. 15, 100257. doi: 10.1016/j.plana.2026.100257 38826717

[B25] Chen C. C. Huang Z. Zhao X. Song Z. Gao M. (2025a). Assessing heterogeneous pollution risks from polystyrene micro(nano)plastics and cadmium to physiology and biochemistry in parsley via a split-root system. Phytochemistry 238, 114565. doi: 10.1016/j.phytochem.2025.114565 40436305

[B26] Chen L. Xu Z. He Y. Zhang X. Li L. Zhu R. . (2025b). Multiomics analysis reveals key targeted metabolic pathways underlying the hormesis and detrimental effects of enrofloxacin on rice plants. J. Agric. Food. Chem. 73, 2678–2695. doi: 10.1021/acs.jafc.4c09001 39834325

[B27] Chen S. Teng Y. Luo Y. Kuramae E. Ren W. (2024a). Threats to the soil microbiome from nanomaterials: A global meta and machine-learning analysis. Soil Biol. Biochem. 188, 109248. doi: 10.1016/j.soilbio.2023.109248 38826717

[B28] Chen S. Zhou S. Xuan X. Niu L. Zhao L. Guo J. . (2025c). 24-epibrassinolide regulates oxytetracycline-induced phytotoxicity and its detoxification mechanism. Ecotoxicol. Environ. Saf. 290, 117763. doi: 10.1016/j.ecoenv.2025.117763 39862695

[B29] Chen X. Chen J. Zhang Y. Ling C. Shen Y. (2025d). Biochar nanoparticles reduce ciprofloxacin accumulation and restore growth and hormonal balance in rice seedlings. Plants 14, 380. doi: 10.3390/plants14030380 39942942 PMC11819727

[B30] Chen X. Ma H. Kong C. Pan T. Gao D. Liao H. . (2024b). Bioaccumulation of polystyrene nanoplastics and BDE-209 induced oxidative stress, photosynthesis and growth impairments in floating fern Salvinia natans. Sci. Total Environ. 909, 168541. doi: 10.1016/j.scitotenv.2023.168541 37979866

[B31] Chu X. Wang J.-G. Li M. Zhang S. Gao Y. Fan M. . (2021). HBI transcription factor-mediated ROS homeostasis regulates nitrate signal transduction. Plant Cell 33, 3004–3021. doi: 10.1093/plcell/koab165 34129038 PMC8462818

[B32] Chukwuneme C. F. Babalola O. O. (2025). Microbial diversity and function in the rhizosphere microbiome: Driving forces and monitoring approaches. Agrosystems Geosci. Environ. 8, e70169. doi: 10.1002/agg2.70169 41531421

[B33] Costa-Broseta Á. Castillo M. León J. (2021). Post-translational modifications of nitrate reductases autoregulates nitric oxide biosynthesis in Arabidopsis. Int. J. Mol. Sci. 22, 549. doi: 10.3390/ijms22020549 33430433 PMC7827142

[B34] Cruz F. V. S. Cardoso S. J. Juneau P. (2025a). Phytoremediation of pesticide-contaminated freshwaters by aquatic plants: A meta-analysis. Chemosphere 383, 144465. doi: 10.1016/j.chemosphere.2025.144465 40479970

[B35] Cruz F. V. S. Venne P. Segura P. Juneau P. (2025b). Effect of temperature on the physiology and phytoremediation capacity of Spirodela polyrhiza exposed to atrazine and S-metolachlor. Aquat. Toxicol. 282, 107304. doi: 10.1016/j.aquatox.2025.107304 40054159

[B36] Dal Ferro N. Pellizzaro A. Fant M. Zerlottin M. Borin M. (2021). Uptake and translocation of perfluoroalkyl acids by hydroponically grown lettuce and spinach exposed to spiked solution and treated wastewaters. Sci. Total Environ. 772, 145523. doi: 10.1016/j.scitotenv.2021.145523 33578170

[B37] Das N. Maji A. (2026). Microbial synergies in phytoremediation: A comprehensive review. Int. J. Adv. Biochem. Res. 10, 191–202. doi: 10.33545/26174693.2026.v10.i1Sc.6909

[B38] Dimaano N. G. Iwakami S. (2021). Cytochrome <scp>P450</scp> ‐mediated herbicide metabolism in plants: Current understanding and prospects. Pest Manage. Sci. 77, 22–32. doi: 10.1002/ps.6040 32776423

[B39] Dreier D. A. Mello D. F. Meyer J. N. Martyniuk C. J. (2019). Linking mitochondrial dysfunction to organismal and population health in the context of environmental pollutants: Progress and considerations for mitochondrial adverse outcome pathways. Environ. Toxicol. Chem. 38, 1625–1634. doi: 10.1002/etc.4453 31034624 PMC6961808

[B40] Du J. Qiu L. Zhou Q. Jin M. Wu W. (2025). Microplastics as vectors for environmental contaminants in the food chain: Assessing the combined toxicological effects and bioavailability. Toxicol. Lett. 413, 111734. doi: 10.1016/j.toxlet.2025.111734 41016659

[B41] Duarte-Hospital C. Tête A. Brial F. Benoit L. Koual M. Tomkiewicz C. . (2021). Mitochondrial dysfunction as a hallmark of environmental injury. Cells 11, 110. doi: 10.3390/cells11010110 35011671 PMC8750015

[B42] El Gemayel L. J. Bashour I. I. (2020). Mechanism of Antibiotics Uptake in Plants. 177–193. doi: 10.1007/978-3-030-40422-2_8

[B43] El-Zohri M. Al-Wadaani N. A. Bafeel S. O. (2021). Foliar sprayed green zinc oxide nanoparticles mitigate drought-induced oxidative stress in tomato. Plants 10, 2400. doi: 10.3390/plants10112400 34834763 PMC8622210

[B44] Fan T. Chen X. Xu Z. Liu L. Shen D. Dong S. . (2020). Uptake and translocation of triflumezopyrim in rice plants. J. Agric. Food. Chem. 68, 7086–7092. doi: 10.1021/acs.jafc.9b07868 32530611

[B45] García-Yagüe Á.J. Cuadrado A. (2023). Mechanisms of NURR1 regulation: Consequences for its biological activity and involvement in pathology. Int. J. Mol. Sci. 24, 12280. doi: 10.3390/ijms241512280 37569656 PMC10419244

[B46] Gomes M. P. (2024a). Climate change and aquatic phytoremediation of contaminants: Exploring the future of contaminant removal. Phyton (B. Aires). 93, 2127–2147. doi: 10.32604/phyton.2024.056360

[B47] Gomes M. P. (2024b). The convergence of antibiotic contamination, resistance, and climate dynamics in freshwater ecosystems. Water 16, 2606. doi: 10.3390/w16182606 30654563

[B48] Gomes M. P. Brito J. C. M. Rocha D. C. Navarro-Silva M. A. Juneau P. (2020). Individual and combined effects of amoxicillin, enrofloxacin, and oxytetracycline on Lemna minor physiology. Ecotoxicol. Environ. Saf. 203, 111025. doi: 10.1016/j.ecoenv.2020.111025 32888593

[B49] Gomes M. P. Brito J. C. M. Vieira F. Kitamura R. Juneau P. (2022a). Emerging contaminants in streams of Doce river watershed, Minas Gerais, Brazil. Front. Environ. Sci. 9, 801599. doi: 10.3389/FENVS.2021.801599/TEXT

[B50] Gomes M. P. Freitas P. L. Kitamura R. S. A. Pereira E. G. Juneau P. (2022b). How aminomethylphosphonic acid (AMPA), the main glyphosate metabolite, interferes with chlorophyll biosynthesis? Environ. Exp. Bot. 203, 105039. doi: 10.1016/j.envexpbot.2022.105039 38826717

[B51] Gomes M. P. Kitamura R. S. A. Marques R. Z. Barbato M. L. Zámocký M. (2022c). The role of H2O2-scavenging enzymes (ascorbate peroxidase and catalase) in the tolerance of Lemna minor to antibiotics: Implications for phytoremediation. Antioxidants 11, 151. doi: 10.3390/antiox11010151 35052655 PMC8772849

[B52] Gomes M. P. Maccario S. Lucotte M. Labrecque M. Juneau P. (2015). Consequences of phosphate application on glyphosate uptake by roots: Impacts for environmental management practices. Sci. Total Environ. 537, 115–119. doi: 10.1016/j.scitotenv.2015.07.054 26282745

[B53] Gomes M. P. Malinoski L. Maranho L. T. Carneiro D. N. M. Richardi V. S. Martinez M. G. (2026). Microbiota modulate metformin phytoremediation and stress responses in Lemna minor. J. Hazard. Mater. 505, 141427. doi: 10.1016/j.jhazmat.2026.141427 41687584

[B54] Gomes M. P. Richardi V. S. Bicalho E. M. da Rocha D. C. Navarro-Silva M. A. Soffiatti P. . (2019a). Effects of ciprofloxacin and Roundup on seed germination and root development of maize. Sci. Total Environ. 651, 2671–2678. doi: 10.1016/j.scitotenv.2018.09.365 30463122

[B55] Gomes M. P. Tavares D. S. Richardi V. S. Marques R. Z. Wistuba N. Moreira de Brito J. C. . (2019b). Enrofloxacin and Roundup® interactive effects on the aquatic macrophyte Elodea canadensis physiology. Environ. pollut. 249, 453–462. doi: 10.1016/j.envpol.2019.03.026 30927690

[B56] Groffen T. Kuijper N. Oden S. Willems T. Bervoets L. Prinsen E. (2023). Growth hormones in broad bean (Vicia faba L.) and radish (Raphanus raphanistrum subsp. sativus L.) are associated with accumulated concentrations of perfluoroalkyl substances. Toxics 11, 922. doi: 10.3390/toxics11110922 37999574 PMC10674852

[B57] Guo J. Li S. Brestic M. Li N. Zhang P. Liu L. . (2023). Modulations in protein phosphorylation explain the physiological responses of barley (Hordeum vulgare) to nanoplastics and ZnO nanoparticles. J. Hazard. Mater. 443, 130196. doi: 10.1016/j.jhazmat.2022.130196 36272376

[B58] Gutiérrez-Martínez P. B. Torres-Morán M. I. Romero-Puertas M. C. Casas-Solís J. Zarazúa-Villaseñor P. Sandoval-Pinto E. . (2020). Assessment of antioxidant enzymes in leaves and roots of Phaseolus vulgaris plants under cadmium stress//Evaluación de enzimas antioxidantes en hojas y raíces de plantas Phaseolus vulgaris bajo estrés de cadmio. Biotecnia 22, 110–118. doi: 10.18633/biotecnia.v22i2.1252

[B59] Hasanuzzaman M. Bhuyan M. H. M. B. Zulfiqar F. Raza A. Mohsin S. M. Al Mahmud J. . (2020). Reactive oxygen species and antioxidant defense in plants under abiotic stress: Revisiting the crucial role of a universal defense regulator. Antioxidants 9, 1–52. doi: 10.3390/ANTIOX9080681 32751256 PMC7465626

[B60] Hauser-Davis R. A. (2026). Beyond total metal burdens: The critical role of intracellular metal compartmentalization in ecotoxicology and environmental risk assessments. Curr. Opin. Environ. Sci. Heal. 50, 100708. doi: 10.1016/j.coesh.2026.100708 38826717

[B61] Herb M. Gluschko A. Schramm M. (2021). Reactive oxygen species: Not omnipresent but important in many locations. Front. Cell Dev. Biol. 9. doi: 10.3389/fcell.2021.716406 34557488 PMC8452931

[B62] Hu P. An J. Faulkner M. M. Wu H. Li Z. Tian X. . (2020). Nanoparticle charge and size control foliar delivery efficiency to plant cells and organelles. ACS Nano 14, 7970–7986. doi: 10.1021/acsnano.9b09178 32628442

[B63] Hu Y. Habibul N. Hu Y.-Y. Meng F.-L. Sheng G.-P. (2021). Chemical speciation of ciprofloxacin in aqueous solution regulates its phytotoxicity and uptake by rice (Oryza sativa L.). Sci. Total Environ. 771, 144787. doi: 10.1016/j.scitotenv.2020.144787 33548726

[B64] Hu S. He R. Zeng J. Zhao D. Wang S. He F. . (2023). Lower compositional variation and higher network complexity of rhizosphere bacterial community in constructed wetland compared to natural wetland. Microb. Ecol. 85, 965–979. doi: 10.1007/s00248-022-02040-6 35641581

[B65] Hu M. Huang Y. Liu L. Ren L. Li C. Yang R. . (2024). The effects of micro/nano-plastics exposure on plants and their toxic mechanisms: A review from multi-omics perspectives. J. Hazard. Mater. 465, 133279. doi: 10.1016/j.jhazmat.2023.133279 38141304

[B66] Ijaz U. Z. Qiu Y. Zhou X. Yin H. Li B. (2025). Editorial: Horizontal transfer of antibiotic resistance genes in the environment: Dynamic, contributing factors, and control. Front. Microbiol. 16. doi: 10.3389/fmicb.2025.1692478 41221393 PMC12597940

[B67] Jain A. Sarsaiya S. Singh R. Gong Q. Wu Q. Shi J. (2024). Omics approaches in understanding the benefits of plant-microbe interactions. Front. Microbiol. 15. doi: 10.3389/fmicb.2024.1391059 38860224 PMC11163067

[B68] Jardim-Messeder D. de Souza-Vieira Y. Sachetto-Martins G. (2025). Dressed up to the nines: The interplay of phytohormones signaling and redox metabolism during plant response to drought. Plants 14, 208. doi: 10.3390/plants14020208 39861561 PMC11768152

[B69] Jian S. Li S. Liu F. Liu S. Gong L. Jiang Y. . (2023). Elevated atmospheric CO2 concentration changes the eco-physiological response of barley to polystyrene nanoplastics. Chem. Eng. J. 457, 141135. doi: 10.1016/j.cej.2022.141135 38826717

[B70] Karpinska B. Foyer C. H. (2024). Superoxide signalling and antioxidant processing in the plant nucleus. J. Exp. Bot. 75, 4599–4610. doi: 10.1093/jxb/erae090 38460122 PMC11317529

[B71] Khan K. Y. Ali B. Ghani H. U. Cui X. Luo X. Ali Z. . (2024). Polyvinyl chloride microplastics and drought co-exposure alter rice growth by affecting metabolomics and proteomics. Sci. Total Environ. 955, 177002. doi: 10.1016/j.scitotenv.2024.177002 39427893

[B72] Khan A. Kanwal F. Ullah S. Fahad M. Tariq L. Altaf M. . (2025). Plant secondary metabolites—central regulators against abiotic and biotic stresses. Metabolites 15, 276. doi: 10.3390/metabo15040276 40278405 PMC12029941

[B73] Kitamura R. S. A. Brito J. C. M. Silva de Assis H. C. Gomes M. P. (2023). Physiological responses and phytoremediation capacity of floating and submerged aquatic macrophytes exposed to ciprofloxacin. Environ. Sci. Pollut. Res. 30, 622–639. doi: 10.1007/s11356-022-22253-z 35904744

[B74] Kochi L. Y. Freitas P. L. Maranho L. T. Juneau P. Gomes M. P. (2020). Aquatic macrophytes in constructed wetlands: A fight against water pollution. Sustain. 12, 1–21. doi: 10.3390/su12219202 30654563

[B75] Kochi L. Y. Kitamura R. S. A. Rocha C. S. Brito J. C. M. Juneau P. Gomes M. P. (2023). Synergistic removal of ciprofloxacin and sulfamethoxazole by Lemna minor and Salvinia molesta in mixed culture: Implications for phytoremediation of antibiotic-contaminated water. Water 15, 1899. doi: 10.3390/w15101899 30654563

[B76] Leitão I. Leclercq C. C. Ribeiro D. M. Renaut J. Almeida A. M. Martins L. L. . (2021a). Stress response of lettuce (Lactuca sativa) to environmental contamination with selected pharmaceuticals: A proteomic study. J. Proteomics 245, 104291. doi: 10.1016/j.jprot.2021.104291 34089899

[B77] Leitão I. Martins L. L. Carvalho L. Oliveira M. C. Marques M. M. Mourato M. P. (2021b). Acetaminophen induces an antioxidative response in lettuce plants. Plants 10, 1152. doi: 10.3390/plants10061152 34204080 PMC8229777

[B78] Leitão I. Mourato M. P. Carvalho L. Oliveira M. C. Marques M. M. Martins L. L. (2021c). Antioxidative response of lettuce (Lactuca sativa) to carbamazepine-induced stress. Environ. Sci. pollut. Res. 28, 45920–45932. doi: 10.1007/s11356-021-13979-3 33881698

[B79] Li B. Zhang Y. Du J. Liu C. Zhou G. Li M. . (2023). Application of multi-omics techniques in aquatic ecotoxicology: A review. Toxics 13, 653. doi: 10.3390/toxics13080653 40863928 PMC12389999

[B80] Li B. Zhang Y. Du J. Liu C. Zhou G. Li M. (2025). Application of Multi-Omics Techniques in Aquatic Ecotoxicology: A Review. Toxics. 13, 653. doi: 10.3390/TOXICS13080653 40863928 PMC12389999

[B81] Li S. (2023). Novel insight into functions of ascorbate peroxidase in higher plants: More than a simple antioxidant enzyme. Redox Biol. 64, 102789. doi: 10.1016/j.redox.2023.102789 37352686 PMC10333675

[B82] Li S. Chiodi C. Maucieri C. Della Lucia M. C. Zardinoni G. Ravi S. . (2026). Profiling soil–plant–microbial communities: DNA and multi-omics techniques. Genes (Basel). 17, 303. doi: 10.3390/genes17030303 41898837 PMC13025542

[B83] Li Y. Lin X. Xu G. Yan Q. Yu Y. (2024b). Toxic effects and mechanisms of engineered nanoparticles and nanoplastics on lettuce (Lactuca sativa L.). Sci. Total Environ. 908, 168421. doi: 10.1016/j.scitotenv.2023.168421 37951267

[B84] Li L. Luo Y. Li R. Zhou Q. Peijnenburg W. J. G. M. Yin N. . (2020a). Effective uptake of submicrometre plastics by crop plants via a crack-entry mode. Nat. Sustain. 3, 929–937. doi: 10.1038/s41893-020-0567-9 37880705

[B85] Li P. Sun J. Xie X. Li Z. Huang B. Zhang G. . (2021). Stress response and tolerance to perfluorooctane sulfonate (PFOS) in lettuce (Lactuca sativa). J. Hazard. Mater. 404, 124213. doi: 10.1016/j.jhazmat.2020.124213 33086182

[B86] Li Q. Tian X. Gu P. Yang G. Deng H. Zhang J. . (2022). Transcriptomic analysis reveals phytohormone and photosynthetic molecular mechanisms of a submerged macrophyte in response to microcystin-LR stress. Aquat. Toxicol. 245, 106119. doi: 10.1016/j.aquatox.2022.106119 35220087

[B87] Li X. Wang X. Ren C. Palansooriya K. N. Wang Z. Chang S. X. (2024a). Microplastic pollution: Phytotoxicity, environmental risks, and phytoremediation strategies. Crit. Rev. Environ. Sci. Technol. 54, 486–507. doi: 10.1080/10643389.2023.2252310 37339054

[B88] Li P. Xiao Z. Sun J. Oyang X. Xie X. Li Z. . (2020b). Metabolic regulations in lettuce root under combined exposure to perfluorooctanoic acid and perfluorooctane sulfonate in hydroponic media. Sci. Total Environ. 726, 138382. doi: 10.1016/j.scitotenv.2020.138382 32481221

[B89] Li Z. (2022). Modeling plant uptake of organic contaminants by root vegetables: The role of diffusion, xylem, and phloem uptake routes. J. Hazard. Mater. 434, 128911. doi: 10.1016/j.jhazmat.2022.128911 35460996

[B90] Liang C. Liu H. (2020). Response of hormone in rice seedlings to irrigation contaminated with cyanobacterial extract containing microcystins. Chemosphere 256, 127157. doi: 10.1016/j.chemosphere.2020.127157 32470740

[B91] López-Climent M. F. Arbona V. Pérez-Clemente R. M. Zandalinas S. I. Gómez-Cadenas A. (2013). Effect of cadmium and calcium treatments on phytochelatin and glutathione levels in citrus plants. Plant Biol. (Stuttg). doi: 10.1111/plb.12006 23574491

[B92] Lu S. Chen J. Wang J. Wu D. Bian H. Jiang H. . (2023). Toxicological effects and transcriptome mechanisms of rice (Oryza sativa L.) under stress of quinclorac and polystyrene nanoplastics. Ecotoxicol. Environ. Saf. 249, 114380. doi: 10.1016/j.ecoenv.2022.114380 36508812

[B93] Lu X. Gao P. Chen Q. Lin Z. Song Z. Lei M. (2025). The response of Chinese cabbage (Brassica rapa L.) to the co-contamination of nanoplastics with different polarity and ketoprofen. Environ. Technol. Innov. 38, 104188. doi: 10.1016/j.eti.2025.104188 38826717

[B94] Luo S. Zhang Y. Gu X. He C. Wang Z. Du H. . (2025). Mechanisms for iron oxide nanoparticle alleviation of nanoplastic-induced stress in Perilla frutescens revealed by integrated physiological and transcriptomic analysis. Plant Physiol. Biochem. 222, 109712. doi: 10.1016/j.plaphy.2025.109712 40024149

[B95] Ma S. Hua Z. Tang C. Qiu X. Ding L. Liang X. . (2025). Root meristem maintenance mechanisms are key to plant defense against nanoplastics. Adv. Sci. 12, e11837. doi: 10.1002/advs.202511837 40856033 PMC12631816

[B96] Ma S. Hua Z. Zhou Q. Meng S. Qiu X. Ding L. . (2026). Disruption of auxin homeostasis by negatively charged nanoplastics inhibits plant primary root development. J. Hazard. Mater. 503, 141140. doi: 10.1016/j.jhazmat.2026.141140 41544595

[B97] Malea P. Kokkinidi D. Kevrekidou A. Adamakis I.-D. (2022). The enzymatic and non-enzymatic antioxidant system response of the seagrass Cymodocea nodosa to bisphenol-A toxicity. Int. J. Mol. Sci. 23, 1348. doi: 10.3390/ijms23031348 35163270 PMC8835922

[B98] Malinoski L. Silva G. G. Rodrigues L. K. I. Carneiro L. F. Gomes M. P. (2026). How glyphosate and its derivatives influence antimicrobial resistance emergence and transmission: A one health perspective. Antibiotics 15, 419. doi: 10.3390/antibiotics15040419 42041382 PMC13114102

[B99] Mallin A. C. Marques R. Z. Akiyama Kitamura R. S. Prodocimo M. M. César de Moraes L. Figueredo C. C. . (2025). Lemna minor as a phytoremediator of cobalt nanoparticles: Insights into toxicity and remediation. Water Biol. Secur., 100442. doi: 10.1016/j.watbs.2025.100442 38826717

[B100] Manna I. Sahoo S. Bandyopadhyay M. (2021). Effect of engineered nickel oxide nanoparticle on reactive oxygen species–nitric oxide interplay in the roots of Allium cepa L. Front. Plant Sci. 12. doi: 10.3389/fpls.2021.586509 33633755 PMC7901573

[B101] Maranho L. T. Geranldo M. R. Nogueira K. d. S. Gomes M. P. (2026). Associations between rhizosphere microbial community structure and antibiotic attenuation in a pilot-scale hybrid constructed wetland. J. Hazard. Mater. 514, 142886. doi: 10.1016/J.JHAZMAT.2026.142886 42430909

[B102] Maranho L. T. Gomes M. P. (2024). Morphophysiological adaptations of aquatic macrophytes in wetland-based sewage treatment systems: Strategies for resilience and efficiency under environmental stress. Plants 13, 2870. doi: 10.3390/plants13202870 39458817 PMC11511398

[B103] Marques R. Z. da Silva Nogueira K. de Oliveira Tomaz A. P. Juneau P. Wang S. Gomes M. P. (2024). Emerging threat: Antimicrobial resistance proliferation during epidemics — A case study of the SARS-CoV-2 pandemic in South Brazil. J. Hazard. Mater. 470, 134202. doi: 10.1016/j.jhazmat.2024.134202 38581873

[B104] Mei W. Sun H. Song M. Jiang L. Li Y. Lu W. . (2021). Per- and polyfluoroalkyl substances (PFASs) in the soil–plant system: Sorption, root uptake, and translocation. Environ. Int. 156, 106642. doi: 10.1016/j.envint.2021.106642 34004449

[B105] Molnár Á. Papp M. Zoltán Kovács D. Bélteky P. Oláh D. Feigl G. . (2020a). Nitro-oxidative signalling induced by chemically synthetized zinc oxide nanoparticles (ZnO NPs) in Brassica species. Chemosphere 251, 126419. doi: 10.1016/j.chemosphere.2020.126419 32171133

[B106] Molnár Á. Rónavári A. Bélteky P. Szőllősi R. Valyon E. Oláh D. . (2020b). ZnO nanoparticles induce cell wall remodeling and modify ROS/ RNS signalling in roots of Brassica seedlings. Ecotoxicol. Environ. Saf. 206, 111158. doi: 10.1016/j.ecoenv.2020.111158 32866892

[B107] Munier C. C. De Maria L. Edman K. Gunnarsson A. Longo M. MacKintosh C. . (2021). Glucocorticoid receptor Thr524 phosphorylation by MINK1 induces interactions with 14-3-3 protein regulators. J. Biol. Chem. 296, 100551. doi: 10.1016/j.jbc.2021.100551 33744286 PMC8080530

[B108] Naing A. H. Maung T. Kim C. K. (2021). The <scp>ACC</scp> deaminase‐producing plant growth‐promoting bacteria: Influences of bacterial strains and <scp>ACC</scp> deaminase activities in plant tolerance to abiotic stress. Physiol. Plant 173, 1992–2012. doi: 10.1111/ppl.13545 34487352

[B109] O’Brien A. L. Dafforn K. A. Chariton A. A. Johnston E. L. Mayer-Pinto M. (2019). After decades of stressor research in urban estuarine ecosystems the focus is still on single stressors: A systematic literature review and meta-analysis. Sci. Total Environ. 684, 753–764. doi: 10.1016/j.scitotenv.2019.02.131 30803690

[B110] Oláh V. Hepp A. Irfan M. Mészáros I. (2021). Chlorophyll fluorescence imaging-based duckweed phenotyping to assess acute phytotoxic effects. Plants 10, 2763. doi: 10.3390/plants10122763 34961232 PMC8707530

[B111] Ozfidan-Konakci C. Yildiztugay E. Arikan B. Alp-Turgut F. N. Turan M. Cavusoglu H. . (2023). Responses of individual and combined polystyrene and polymethyl methacrylate nanoplastics on hormonal content, fluorescence/photochemistry of chlorophylls and ROS scavenging capacity in Lemna minor under arsenic-induced oxidative stress. Free Radic. Biol. Med. 196, 93–107. doi: 10.1016/j.freeradbiomed.2023.01.015 36657731

[B112] Pande P. M. Tremblay J. St-Arnaud M. Yergeau E. (2025). The metatranscriptomic response of the wheat rhizosphere to drought varies with growth stages. doi: 10.1101/2025.11.26.690733 PMC13082227 41993800

[B113] Parveen K. Kumari R. Malaviya P. (2023). Impact of pharmaceutical industry wastewater on stress physiological responses of Spirodela polyrhiza (L.) Schleiden. Environ. Sci. pollut. Res. 30, 119275–119284. doi: 10.1007/s11356-023-30729-9 37924407

[B114] Ravi B. Foyer C. H. Pandey G. K. (2023). The integration of reactive oxygen species (ROS) and calcium signalling in abiotic stress responses. Plant Cell Environ. 46, 1985–2006. doi: 10.1111/pce.14596 37132157

[B115] Rizaludin M. S. Stopnisek N. Raaijmakers J. M. Garbeva P. (2021). The chemistry of stress: Understanding the ‘Cry for Help’ of plant roots. Metabolites 11, 357. doi: 10.3390/metabo11060357 34199628 PMC8228326

[B116] Sabir F. Di Pizio A. Loureiro-Dias M. C. Casini A. Soveral G. Prista C. (2020). Insights into the selectivity mechanisms of grapevine NIP aquaporins. Int. J. Mol. Sci. 21, 6697. doi: 10.3390/ijms21186697 32933135 PMC7576499

[B117] Salehi H. De Diego N. Chehregani Rad A. Benjamin J. J. Trevisan M. Lucini L. (2021). Exogenous application of ZnO nanoparticles and ZnSO4 distinctly influence the metabolic response in Phaseolus vulgaris L. Sci. Total Environ. 778, 146331. doi: 10.1016/j.scitotenv.2021.146331 33725605

[B118] Santoyo G. Guzmán-Guzmán P. Parra-Cota F. I. Santos-Villalobos S. Orozco-Mosqueda M. C. Glick B. R. (2021). Plant growth stimulation by microbial consortia. Agronomy 11, 219. doi: 10.3390/agronomy11020219 30654563

[B119] Shahid M. Singh U. B. Khan M. S. Singh P. Kumar R. Singh R. N. . (2023). Bacterial ACC deaminase: Insights into enzymology, biochemistry, genetics, and potential role in amelioration of environmental stress in crop plants. Front. Microbiol. 14. doi: 10.3389/fmicb.2023.1132770 37180266 PMC10174264

[B120] Shao H. Wang H. Ding Y. Chen S. Zeng L. Luo L. . (2026). Integrated physiological, transcriptomic, and metabolic analysis reveals the effects of nanoplastics exposure on tea plants. J. Hazard. Mater. 501, 140862. doi: 10.1016/j.jhazmat.2025.140862 41418461

[B121] Shitaw E. E. AlAhmad M. Sivaprasadarao A. (2025). Inter-organelle crosstalk in oxidative distress: A unified TRPM2-NOX2 mediated vicious cycle involving Ca2+, Zn2+, and ROS amplification. Antioxidants 14, 776. doi: 10.3390/antiox14070776 40722879 PMC12291955

[B122] Shukla V. Barberon M. (2021). Building and breaking of a barrier: Suberin plasticity and function in the endodermis. Curr. Opin. Plant Biol. 64, 102153. doi: 10.1016/j.pbi.2021.102153 34861611

[B123] Singh A. Mazahar S. Chapadgaonkar S. S. Giri P. Shourie A. (2023). Phyto-microbiome to mitigate abiotic stress in crop plants. Front. Microbiol. 14. doi: 10.3389/fmicb.2023.1210890 37601386 PMC10433232

[B124] Soares C. Nadais P. Sousa B. Pinto E. Ferreira I. M. P. L. V. O. Pereira R. . (2021). Silicon improves the redox homeostasis to alleviate glyphosate toxicity in tomato plants—Are nanomaterials relevant? Antioxidants 10, 1320. doi: 10.3390/antiox10081320 34439568 PMC8389300

[B125] Sobiecka E. Mroczkowska M. Olejnik T. P. (2022). The enzymatic antioxidants activities changes in water plants tissues exposed to chlorpyrifos stress. Antioxidants 11, 2104. doi: 10.3390/antiox11112104 36358476 PMC9686717

[B126] Stading R. Chu C. Couroucli X. Lingappan K. Moorthy B. (2020). Molecular role of cytochrome P4501A enzymes in oxidative stress. Curr. Opin. Toxicol. 20–21, 77–84. doi: 10.1016/j.cotox.2020.07.001 33283080 PMC7709944

[B127] Sun H. Lei C. Xu J. Li R. (2021). Foliar uptake and leaf-to-root translocation of nanoplastics with different coating charge in maize plants. J. Hazard. Mater. 416, 125854. doi: 10.1016/j.jhazmat.2021.125854 33892383

[B128] Tang X. Hao F. Yuan H. Yan X. Yang D. Taylor A. G. (2020). Uptake and translocation of imidacloprid via seed pathway and root pathway during early seedling growth of corn. Pest Manage. Sci. 76, 3792–3799. doi: 10.1002/ps.5930 32452624

[B129] Tansel B. (2026). Noncovalent interactions as key modulators of PFAS translocation, lipid-protein affinity, and tissue partitioning. J. Hazard. Mater. 501, 140933. doi: 10.1016/j.jhazmat.2025.140933 41456435

[B130] Thakur N. Sharma P. Negi N. Sharma N. Sharma P. (2025). Decoding oxidative stress regulation in food crops exposed to heavy metals: Interdisciplinary strategies for sustainable mitigation. Discov. Sustain. 6, 904. doi: 10.1007/s43621-025-00912-8 30311153

[B131] Tripathi S. Sharma S. Rai P. Mahra S. Tripathi D. K. Sharma S. (2024). Synergy of plant growth promoting rhizobacteria and silicon in regulation of AgNPs induced stress of rice seedlings. Plant Physiol. Biochem. 213, 108720. doi: 10.1016/j.plaphy.2024.108720 38901227

[B132] Trisotto L. F. R. Figueredo C. C. Gomes M. P. (2025). Rivers at risks: The interplay of “COVID kit” medication misuse and urban waterway contaminants. Chemosphere 370, 143933. doi: 10.1016/j.chemosphere.2024.143933 39672345

[B133] Trivedi P. Leach J. E. Tringe S. G. Sa T. Singh B. K. (2020). Plant–microbiome interactions: From community assembly to plant health. Nat. Rev. Microbiol. 18, 607–621. doi: 10.1038/s41579-020-0412-1 32788714

[B134] UNEP (2019). Global Chemicals Outlook II - From Legacies to Innovative Solutions: Implementing the 2030 Agenda for Sustainable Development. Available online at: https://wedocs.unep.org/handle/20.500.11822/28113 (Accessed July 19, 2026).

[B135] Upadhyay S. K. (2025). Relevance of cross talk between root exudates, hormones, and root-associated microbes in developing sustainable phytoremediation strategies: A comprehensive review. Physiol. Mol. Biol. Plants 31, 1629–1649. doi: 10.1007/s12298-025-01593-3 41164111 PMC12559568

[B136] Vitelli V. Giamborino A. Bertolini A. Saba A. Andreucci A. (2024). Cadmium stress signaling pathways in plants: Molecular responses and mechanisms. Curr. Issues Mol. Biol. 46, 6052–6068. doi: 10.3390/cimb46060361 38921032 PMC11202648

[B137] Vivek K. (2025). Biochemical changes in aquatic weeds in response to perchlorate contamination, an *in vitro* study. Environ. Ecol. 43, 425–432. doi: 10.60151/envec/DJIJ6750 27598717

[B138] Voloshina M. Rajput V. Minkina T. Vechkanov E. Mandzhieva S. Mazarji M. . (2022). Zinc oxide nanoparticles: Physiological and biochemical responses in barley (Hordeum vulgare L.). Plants 11, 2759. doi: 10.3390/plants11202759 36297783 PMC9607964

[B139] Vymazal J . (2019). Is removal of organics and suspended solids in horizontal sub-surface flow constructed wetlands sustainable for twenty and more years? Chem. Eng. J. 378, 122117. doi: 10.1016/J.CEJ.2019.122117 36297783

[B140] Wang J. Liu W. Wang X. Zeb A. Wang Q. Mo F. . (2024). Assessing stress responses in potherb mustard (Brassica juncea var. multiceps) exposed to a synergy of microplastics and cadmium: Insights from physiology, oxidative damage, and metabolomics. Sci. Total Environ. 907, 167920. doi: 10.1016/j.scitotenv.2023.167920 37863229

[B141] Wang X. Xie H. Wang P. Yin H. (2023). Nanoparticles in plants: Uptake, transport and physiological activity in leaf and root. Materials (Basel). 16, 3097. doi: 10.3390/ma16083097 37109933 PMC10146108

[B142] Wang T.-T. Ying G.-G. Shi W.-J. Zhao J.-L. Liu Y.-S. Chen J. . (2020). Uptake and translocation of perfluorooctanoic acid (PFOA) and perfluorooctanesulfonic acid (PFOS) by wetland plants: Tissue- and cell-level distribution visualization with desorption electrospray ionization mass spectrometry (DESI-MS) and transmission ele. Environ. Sci. Technol. 54, 6009–6020. doi: 10.1021/acs.est.9b05160 32324390

[B143] Wei W. Ling S. Wu X. Li X. (2023b). Geochemical accumulation and source tracing of heavy metals in arable soils from a black shale catchment, southwestern China. Sci. Total Environ. 857, 159467. doi: 10.1016/j.scitotenv.2022.159467 36257439

[B144] Wei H. Tang M. Xu X. (2023a). Mechanism of uptake, accumulation, transport, metabolism and phytotoxic effects of pharmaceuticals and personal care products within plants: A review. Sci. Total Environ. 892, 164413. doi: 10.1016/j.scitotenv.2023.164413 37247738

[B145] Wei Y. H. Wang D. Q. Li G. S. Yu H. C. Dong X. W. Jiang H. Q. (2023c). Research on the descaling characteristics of a new electrochemical water treatment device. Water Sci. Technol. 88, 2566–2580. doi: 10.2166/wst.2023.365 38017678

[B146] Wentzell B. M. (2025). Phytoremediation for water quality improvement: Current advances and future prospects. Bio/Technol. Environ. 2, 7. doi: 10.1186/s44314-025-00022-9 38164791

[B147] Wilkinson J. L. Boxall A. B. A. Kolpin D. W. Leung K. M. Y. Lai R. W. S. Galbán-Malagón C. . (2022). Pharmaceutical pollution of the world’s rivers. Proc. Natl. Acad. Sci. 119, e2113947119. doi: 10.1073/pnas.2113947119 35165193 PMC8872717

[B148] Xie H. Wei C. Wang W. Chen R. Cui L. Wang L. . (2024). Screening the phytotoxicity of micro/nanoplastics through non-targeted metallomics with synchrotron radiation X-ray fluorescence and deep learning: Taking micro/nano polyethylene terephthalate as an example. J. Hazard. Mater. 463, 132886. doi: 10.1016/j.jhazmat.2023.132886 37913659

[B149] Xie Z. Zeng Y. Zhu S. Zhu M. Yu Y. Cheng K. . (2026). From energy collapse to chemical defense: Microplastics reshape the metabolic landscape of Tetrastigma hemsleyanum (Vitaceae). Plant Physiol. Biochem. 231, 111007. doi: 10.1016/j.plaphy.2025.111007 41518827

[B150] Xu C. (2024). The Oryza sativa transcriptome responds spatiotemporally to polystyrene nanoplastic stress. Sci. Total Environ. 928, 172449. doi: 10.1016/j.scitotenv.2024.172449 38615784

[B151] Xu M. Li R. Liu W. (2025). Deciphering the molecular mechanisms underlying abscisic acid-alleviated growth inhibition caused by perfluorooctane sulfonic acid in tomato plants. J. Hazard. Mater. 495, 139109. doi: 10.1016/j.jhazmat.2025.139109 40602123

[B152] Xu L. Liu C. Ren Y. Huang Y. Liu Y. Feng S. . (2024). Nanoplastic toxicity induces metabolic shifts in Populus × euramericana cv. “74/76” revealed by multi-omics analysis. J. Hazard. Mater. 470, 134148. doi: 10.1016/j.jhazmat.2024.134148 38565012

[B153] Yang R. Chen M. Cheng L. Cui Y. Li C. Zhang Y. (2025). Molecular mechanisms underlying microplastics-induced inhibition of lateral root development in tomato (Solanum lycopersicum L.). J. Environ. Manage. 395, 127848. doi: 10.1016/j.jenvman.2025.127848 41207253

[B154] Yang J. Smagghe G. Wu X. Zhang W. Chen X. (2026). Divergent impacts of conventional and biodegradable microplastics on pesticide fate and toxicity in a soil–chive system, underscoring a soil-plant-microbe disruption. Pestic. Biochem. Physiol. 218, 106971. doi: 10.1016/j.pestbp.2026.106971 41629037

[B155] Yang W. Zhang H. Yang S. Xiao Y. Ye K. He R. . (2024). Combined effects of microplastics and pharmaceutical and personal care products on algae: A critical review. Environ. pollut. 358, 124478. doi: 10.1016/j.envpol.2024.124478 38950849

[B156] Yildiztugay E. Ozfidan-Konakci C. Arikan B. Alp F. N. Elbasan F. Zengin G. . (2022). The hormetic dose-risks of polymethyl methacrylate nanoplastics on chlorophyll a fluorescence transient, lipid composition and antioxidant system in Lactuca sativa. Environ. pollut. 308, 119651. doi: 10.1016/j.envpol.2022.119651 35752396

[B157] Yoshimura K. Ishikawa T. (2024). Physiological function and regulation of ascorbate peroxidase isoforms. J. Exp. Bot. 75, 2700–2715. doi: 10.1093/jxb/erae061 38367016

[B158] Yu H. Peng J. Cao X. Wang Y. Zhang Z. Xu Y. . (2021). Effects of microplastics and glyphosate on growth rate, morphological plasticity, photosynthesis, and oxidative stress in the aquatic species Salvinia cucullata. Environ. pollut. 279, 116900. doi: 10.1016/j.envpol.2021.116900 33744626

[B159] Yu Z. Xu X. Guo L. Jin R. Lu Y. (2024). Uptake and transport of micro/nanoplastics in terrestrial plants: Detection, mechanisms, and influencing factors. Sci. Total Environ. 907, 168155. doi: 10.1016/j.scitotenv.2023.168155 37898208

[B160] Yue L. Ye A. Uwaremwe C. Xie X. Turatsinze A. N. Jin L. . (2026). JA-dependent ROS modulation by acetoin-rich volatiles from amyloliquefaciens FZB42 triggers stomatal closure and enhances abiotic stress tolerance in Arabidopsis. Microbiol. Res. 305, 128427. doi: 10.1016/j.micres.2025.128427 41435632

[B161] Yusefi-Tanha E. Fallah S. Rostamnejadi A. Pokhrel L. R. (2020). Particle size and concentration dependent toxicity of copper oxide nanoparticles (CuONPs) on seed yield and antioxidant defense system in soil grown soybean (Glycine max cv. Kowsar). Sci. Total Environ. 715, 136994. doi: 10.1016/j.scitotenv.2020.136994 32041054

[B162] Zaidi S. Hayat S. Pichtel J. (2024). Arsenic-induced plant stress: Mitigation strategies and omics approaches to alleviate toxicity. Plant Physiol. Biochem. 213, 108811. doi: 10.1016/j.plaphy.2024.108811 38870680

[B163] Zeeshan M. Sun C. Wang X. Hu Y. Wu H. Li S. . (2024). Insights into the ameliorative effect of ZnONPs on arsenic toxicity in soybean mediated by hormonal regulation, transporter modulation, and stress responsive genes. Front. Plant Sci. 15. doi: 10.3389/fpls.2024.1427367 39139724 PMC11319271

[B164] Zhang Y. Chen Y. Jiao R.-Q. Gao S.-S. Li B. L. Li Y.-Y. . (2025). Beneficial microbial consortia effectively alleviated plant stress caused by the synergistic toxicity of microplastics and cadmium. Ind. Crops Prod. 225, 120479. doi: 10.1016/j.indcrop.2025.120479 38826717

[B165] Zhang T. Li N. Chen G. Xu J. Ouyang G. Zhu F. (2021). Stress symptoms and plant hormone-modulated defense response induced by the uptake of carbamazepine and ibuprofen in Malabar spinach (Basella alba L.). Sci. Total Environ. 793, 148628. doi: 10.1016/j.scitotenv.2021.148628 34328997

[B166] Zhang W. Li Q. Yang Y. Yu Y. Li S. Liu J. . (2023). Joint toxicity mechanisms of perfluorooctanoic acid and sulfadiazine on submerged macrophytes and periphytic biofilms. J. Hazard. Mater. 458, 131910. doi: 10.1016/J.JHAZMAT.2023.131910 37390681

[B167] Zhang L. Vaccari F. Ardenti F. Fiorini A. Tabaglio V. Puglisi E. . (2024). The dosage- and size-dependent effects of micro- and nanoplastics in lettuce roots and leaves at the growth, photosynthetic, and metabolomics levels. Plant Physiol. Biochem. 208, 108531. doi: 10.1016/j.plaphy.2024.108531 38513516

[B168] Zhang L. Yan C. Qi R. Yang F. (2022). Quantifying the contribution rates of sulfonamide antibiotics removal mechanisms in constructed wetlands using multivariate statistical analysis. Environ. pollut. 292, 118463. doi: 10.1016/j.envpol.2021.118463 34742821

[B169] Zhao Z. Zheng X. Han Z. Yang S. Zhang H. Lin T. . (2023). Response mechanisms of Chlorella sorokiniana to microplastics and PFOA stress: Photosynthesis, oxidative stress, extracellular polymeric substances and antioxidant system. Chemosphere 323, 138256. doi: 10.1016/j.chemosphere.2023.138256 36858114

[B170] Zwiewka M. Bielach A. Tamizhselvan P. Madhavan S. Ryad E. E. Tan S. . (2019). Root adaptation to H2O2-induced oxidative stress by ARF-GEF BEN1- and cytoskeleton-mediated PIN2 trafficking. Plant Cell Physiol. 60, 255–273. doi: 10.1093/pcp/pcz001 30668780

