# Supplementary material for: Plant stress physiology under environmental emerging contaminant exposure: from molecular responses to phytoremediation applications
Source: Front Plant Sci. 2026 Jul 27;17:1877141. doi: 10.3389/fpls.2026.1877141 (PMC13453730; doi:10.3389/fpls.2026.1877141)
Supplement: Supplementary file 1 [file Table1.docx]

**Plant Stress Physiology Under Environmental Emerging Contaminant Exposure: From Molecular Responses to Phytoremediation Applications**

Marcelo Pedrosa Gomes^1^, Leila Teresinha Maranho^1^, Letícia Estela Cavichiolo Espindola^1^, Flávia Yoshie Yamamoto^1^, Kui Xu^2^, David Dewez^3^

^1^Laboratório de Fisiologia de Plantas sob Estresse, Departamento de Botânica, Setor de Ciências Biológicas, Universidade Federal do Paraná, Avenida Coronel Francisco H. dos Santos, 100, Centro Politécnico Jardim das Américas, C.P. 19031, 81531-980, Curitiba, Paraná, Brazil

^2^Sun Yat-sen University, Guangzhou, China

^3^University of Quebec in Montreal, Montreal, Canada

**Table S1.** Physiological, biochemical, and molecular biomarkers commonly used to assess plant responses to emerging environmental contaminants.

| Biomarker category | Representative biomarkers* | Physiological meaning | Typical analytical methods | Relevance for contaminant stress studies | References |
| --- | --- | --- | --- | --- | --- |
| Growth and morphology | RGR, biomass, root length, leaf area | Overall plant performance and energy allocation | Biomass measurement, image analysis | Integrative stress endpoints | (Gong et al., 2021; Arikan et al., 2022; Xu et al., 2024) |
| Photosynthetic performance | Fv/Fm, ETR, NPQ, chlorophyll content | Photosystem II efficiency and photochemical balance | PAM fluorometry, SPAD, gas exchange | Early stress detection | (Gomes et al., 2020; Yildiztugay et al., 2022; Kochi et al., 2023; Xu et al., 2024) |
| Oxidative stress markers | H₂O₂, O₂⁻, MDA | Redox imbalance and oxidative damage | Spectrophotometry, fluorescence probes | Core indicators of contaminant stress | (Leitão et al., 2021b, 2021a; Arikan et al., 2022; Voloshina et al., 2022) |
| Antioxidant enzymes | SOD, CAT, APX, POD, GR | ROS detoxification capacity | Enzyme assays | Defense activation and tolerance mechanisms | (Adhikari et al., 2020; Voloshina et al., 2022; Mallin et al., 2025) |
| Detoxification metabolism | P450, GST, UGTs | Xenobiotic transformation | Enzyme assays, transcriptomics | Detoxification pathways | (Lu et al., 2023; Abdelmoneim et al., 2024; Chakraborty et al., 2026) |
| Nitrogen metabolism | NR, GS, GOGAT, NH₄⁺ | Nutrient assimilation and metabolic balance | Enzyme assays, metabolite quantification | Sensitive to contaminant stress | (Gomes et al., 2022; Liu et al., 2022; Shoaib et al., 2025) |
| Phytohormones | IAA, ABA, SA, ACC, JA | Stress signaling and regulation | LC-MS/MS, immunoassays | Coordination of stress responses | (Arikan et al., 2022; Ozfidan-Konakci et al., 2023; Rao et al., 2025) |
| Secondary metabolites | Phenolics, flavonoids, anthocyanins | Antioxidant and defense responses | HPLC, spectrophotometry | Long-term stress adaptation | (Malea et al., 2022; Xu et al., 2024; Xie et al., 2026) |
| Omics responses | Transcriptomics, proteomics, metabolomics | Systems-level responses | RNA-seq, LC-MS/MS | Mechanistic insights | (Kafle et al., 2022; Yu et al., 2022; Shoaib et al., 2025) |
| Bioaccumulation | Tissue concentration, BCF, TF | Uptake and transport | LC-MS/MS, GC-MS | Phytoremediation relevance | (Li et al., 2020b, 2022a; Lu et al., 2025) |

*These endpoints are mechanistically interconnected and should not be interpreted independently, as oxidative stress, detoxification, and hormonal regulation form a tightly coupled physiological network in response to contaminant exposure.

**Table S2.** Uptake, translocation, and kinetic descriptors of emerging contaminants in plants (post-2019 literature)

| Contaminant class | Representative compound | Plant species | Exposure system | Exposure concentration | Duration | Uptake route | TF (shoot/root) | BCF/BAF | Uptake rate / kinetic descriptor | Main translocation pathway | Main accumulation organ | Molecular weight (g/mol) | logKow | pKa | DOI |
| --- | --- | --- | --- | --- | --- | --- | --- | --- | --- | --- | --- | --- | --- | --- | --- |
| Pharmaceuticals (antibiotic) | Ciprofloxacin | *Oryza sativa* L. | Hydroponic; solution pH-gradient study | Concentration-dependent (article reports gradient; exact tested series not visible in abstract) | Up to 72 h for root adsorption; plant exposure period reported in article | Root | NR | NR | Pseudo-first-order adsorption on excised roots; uptake decreased as pH increased | Xylem after root entry | Roots, stems and leaves all accumulated CIP | 331.34 | 0.28 | 6.09; 8.74 | (Hu et al., 2021) |
| Insecticide | Imidacloprid | *Zea mays* L. | Seed pathway and root pathway during early seedling growth | Seed coating / root exposure experiment (see article for exact dose design) | Early seedling stage; published kinetics over initial growth period | Seed and root | NR | NR | Compared seed- versus root-derived uptake and translocation during early growth | Xylem-dominated systemic movement | Shoots and leaves after systemic translocation | 255.66 | 0.57 | 11.1 | (Tang et al., 2020) |
| Insecticide | Triflumezopyrim | *Oryza sativa* | Hydroponic and foliar treatment | 2.5 and 5 mg L⁻¹ | 24, 48 and 72 h | Root and foliar | NR | NR | Systemic distribution after hydroponic uptake; foliar uptake to lower leaves, but none detected in roots | Xylem after root uptake; limited downward redistribution after foliar exposure | Shoots/leaves under hydroponic and foliar treatments | 398.74 | 1.26 | NA | (Fan et al., 2020) |
| PFAS | Perfluoroalkyl acids (PFAAs) | *Lactuca sativa* L.; *Spinacia oleracea* L. | Hydroponic; spiked solution and treated wastewaters | 500 ng L⁻¹ each PFAA in spiked solution; treated WWTP effluents also tested | Hydroponic exposure period reported in article | Root | Up to 15-fold higher in spinach than lettuce | Wastewater concentration slightly affected BCFs | Sigmoidal root concentration factor at increasing PFCA chain length | Xylem | Roots and shoots; lettuce accumulated more than spinach | Class-dependent | Chain-length dependent | Strong acids | (Dal Ferro et al., 2021) |
| PFAS | Nine PFASs (including short- and long-chain homologues) | *Lactuca sativa* | Hydroponic | Nine PFASs in hydroponic solution (see article for individual concentrations) | Uptake experiment duration reported in article | Root | Average TF negatively related to molecular volume | Average root concentration factors positively correlated with logKow | Long-chain PFASs (≥C8) accumulated more in roots; short-chain (≤C7) preferentially transported to shoots | Xylem | Roots for long-chain; shoots for short-chain PFASs | Class-dependent | Class-dependent | Strong acids | (Wang et al., 2022) |
| PFAS | Legacy and emerging PFASs (17 compounds) | Lettuce, Chinese cabbage, chrysanthemum coronarium, cucumber | Hydroponic | 10 μg L⁻¹ each PFAS; 8:2 Cl-PFESA at 0.5 μg L⁻¹ | Hydroponic exposure period reported in article | Root | Appreciable but species-dependent for HFPO-DA and 8:2 FTSA; 8:2 Cl-PFESA hardly translocated | Root concentration factor 35.7–99.9 for HFPO-TA | Emerging substitutes showed stronger root retention or greater edible-part transfer depending on chemistry | Xylem | Roots for HFPO-TA and 8:2 Cl-PFESA; edible tissues for HFPO-DA and FTSAs | Class-dependent | Class-dependent | Strong acids | (Gu et al., 2023) |
| PFAS | PFOA; PFOS | Eight wetland macrophytes | Hydroponic | Hydroponic spiking experiment (see article for exact concentration) | Experiment duration reported in article | Root | PFOA translocated more readily than PFOS | Plants accumulated 1.67–16.7% of total spiked mass | PFOS accumulated largely in roots (48.8–95.8%); PFOA stored mostly aboveground (29.3–77.4%) | Apoplastic and symplastic root entry; xylem/cortex transport to shoots | PFOS roots; PFOA aboveground tissues | 414.07; 500.13 | 4.06; 4.49 | ~2.8; <0 | (Wang et al., 2020) |
| Micro-/nanoplastics | Polystyrene and PMMA particles | *Triticum aestivum*; *Lactuca sativa* | Hydroponic, sand, and sandy soil | Submicrometre and micrometre particles in treated wastewater matrices (see article for design) | Exposure period reported in article | Root | NR | NR | Higher transpiration enhanced uptake; submicrometre particles were taken up more efficiently | Crack-entry into stele followed by transpiration-driven upward transport | Roots and shoots | Polymer | NA | NA | (Li et al., 2020a) |
| Nanoplastics | PS-COOH and PS-NH2 nanoplastics | *Zea mays* L. | Foliar exposure | Foliar nanoplastic exposure (22–24 nm particles; see article for applied suspension concentration) | Exposure time-course reported in article | Leaf | Leaf-to-root translocation demonstrated; aggregation limited root transfer | NR | Positive particles associated more strongly with leaves; rootward transport decreased with aggregation | Stomatal entry to vasculature, then leaf-to-root movement through vascular bundle | Leaves, with subsequent root detection | Polymer | NA | Surface-functionalized particles | (Sun et al., 2021) |

TF = translocation factor (shoot/root). BCF/BAF = bioconcentration/bioaccumulation factor. NR = not reported in the primary paper. Physicochemical constants are shown only when a single small molecule is represented by the row; class-level rows or polymeric particles are marked as class-dependent or not applicable.

**Table S4.** Organ-resolved synthesis of hormone-mediated plant responses to emerging environmental contaminants (2019-present).

| Contaminant(s) | Contaminant class | Plant species | Organ / tissue | Exposure system | Hormonal pathway(s) affected | Hormonal response | Mechanistic interpretation | Main physiological outcome | Reference |
| --- | --- | --- | --- | --- | --- | --- | --- | --- | --- |
| Pharmaceutical mixture | Pharmaceuticals | Lettuce, cucumber | Whole seedling | Hydroponic | Auxin, ABA | Hormone homeostasis altered under chronic low-dose mixture exposure | Chronic mixture exposure likely perturbed growth-regulating and stress-related hormonal balance rather than inducing acute collapse | Altered germination and growth under environmentally relevant chronic exposure | (McGinnis et al., 2019) |
| Carbamazepine, ibuprofen | Pharmaceuticals | *Basella alba* | Whole plant | Root exposure / hydroponic-style system | ABA, auxin, defense-related hormones | Hormonal disruption has been reported. | Metabolic disturbance and oxidative stress likely redirected signaling from growth to defense | Impaired growth and activation of defense responses | (Zhang et al., 2021) |
| Ciprofloxacin | Pharmaceuticals | Rice | Roots and shoots | Likely hydroponic / controlled exposure | Auxin, ABA, detox-linked hormonal regulation | Auxin decreased within the broader reported range of 14.5–64.3%; mitigation-associated hormonal recovery reported | Antibiotic stress induced ROS-linked hormonal imbalance; mitigation restored growth–defense coordination | Reduced growth and stress tolerance under contamination; partial recovery under mitigation | (Chen et al., 2025b) |
| Chlortetracycline | Pharmaceuticals | Lettuce | Whole plant | Controlled exposure | ABA, auxin | Hormetic response reported; low-dose resilience with higher dose injury; | Pharmaceutical exposure induced non-linear hormonal regulation consistent with ROS-threshold behavior | Growth stimulation at low dose and inhibition at higher dose | (Choe et al., 2024) |
| Enrofloxacin | Pharmaceuticals | Rice | Whole plant | Controlled exposure | Auxin, ABA, stress hormones | Hormesis has been reported, with low-dose stimulation and high-dose suppression. | Redox-dependent shift from adaptive signaling to endocrine/metabolic disruption | Transition from growth promotion to injury with increasing dose | (Chen et al., 2025b) |
| Glyphosate-based herbicide | Pesticide / herbicide | Oat, potato, strawberry | Whole plant | Exposure not fully resolved in report | ABA, auxin, species-specific hormonal networks | ABA increased in line with the common 2.0–3.0-fold stress pattern; species-specific variation emphasized | Herbicide-driven oxidative disruption altered hormonal homeostasis differently across species | Species-specific stress phenotypes and altered development | (Fuchs et al., 2022) |
| Bisphenol A | Organic pollutant / endocrine-active contaminant | Pepper | Leaves and likely roots | Soil or substrate exposure | ABA, stress-related signaling | Hormonal stress signature reported | BPA-induced oxidative pressure likely triggered ABA-centered stress signaling and photophysiological dysfunction | Photosynthetic toxicity and stress syndrome | (Huang et al., 2025) |
| Bisphenol A | Organic pollutant / endocrine-active contaminant | *Arabidopsis thaliana* | Roots | Controlled exposure | Ethylene, auxin | Hormone-mediated root inhibition reported | BPA-induced root growth suppression was linked to signaling interference rather than purely structural toxicity | Root growth inhibition | (Bahmani et al., 2020) |
| Dibutyl phthalate | Organic pollutant | *Brassica rapa* var. chinensis | Seeds / seedlings | Likely soil exposure | JA, SA, defense hormones | Defense-related hormonal perturbation reported | Organic pollutant exposure activated defense-related signaling and altered early development | Germination and early growth impairment | (Ge et al., 2020) |
| PFAS mixture | PFAS | Radish, broad bean | Whole plant | Controlled exposure | ABA and broader hormone associations | Hormone–accumulation associations reported; ABA-centered stress regulation implicated | Persistent contaminants promoted chronic endocrine reconfiguration linked to accumulation and stress adaptation | Altered growth and contaminant accumulation patterns | (Groffen et al., 2023) |
| PFOS | PFAS | Tomato | Likely roots and shoots | Controlled exposure | ABA | ABA-mediated stress adaptation reported | Chronic oxidative imbalance likely positioned ABA as a long-term regulator of contaminant tolerance | Stress acclimation under persistent contaminant exposure | (Chen et al., 2025a) |
| Polystyrene nanoplastics (20–1000 nm) | Nanoplastics | *Arabidopsis thaliana* | Roots (meristem) | Controlled plate / seedling system | Auxin | Auxin pathway disruption; within the broader nanoplastic/pharmaceutical range, IAA decreased 14.5–64.3% | 20 nm particles disrupted PIN2-mediated auxin distribution, destabilizing meristem maintenance | Strong root growth inhibition and meristem disorganization | (Ma et al., 2026) |
| Polystyrene nanoplastics | Nanoplastics | Rice | Roots | Controlled exposure | Auxin | Auxin restoration under mitigation reported; low-dose or mitigation-related IAA increase fits the 30.1–154.4% range | Zinc-related mitigation appears to buffer ROS and partially restore auxin homeostasis | Improved tolerance relative to nanoplastic stress alone | (Xu, 2024) |
| Polystyrene and PMMA nanoplastics plus arsenic | Nanoplastics + metalloid co-stress | *Lemna minor* | Whole plant | Hydroponic | ABA, oxidative-stress-associated hormones | ABA decreased by approximately 16.0% under nanoplastic-associated co-stress scenario | Reduced ABA buffering capacity paradoxically coincided with enhanced arsenic toxicity, indicating maladaptive signaling under co-exposure | Increased toxicity despite activation of some antioxidant defenses | (Ozfidan-Konakci et al., 2023) |
| Polystyrene nanoplastics | Nanoplastics | Soybean | Whole plant | Controlled exposure | Auxin, ABA, stress-related signaling | Hormonal disruption reported | Toxicity likely involved ROS-associated transcriptomic reprogramming of growth and defense pathways | Physiological stress and molecular disruption | (Qiu et al., 2023) |
| Polystyrene nanoplastics | Nanoplastics | Tomato | Whole plant | Controlled exposure | Brassinosteroid interaction, auxin, ABA | Hormone restoration under brassinosteroid mitigation reported | Brassinosteroids partially counteracted nanoplastic toxicity by rebalancing ROS–hormone crosstalk | Improved tolerance and reduced oxidative damage under mitigation | (Gao et al., 2023) |
| PVC nanoplastics | Nanoplastics | Radish | Whole plant | Controlled exposure | Hormone signaling broadly | Hormonal signaling interference reported | PVC nanoplastics altered hormonal regulatory architecture rather than affecting a single pathway | Developmental and stress-related disruption | (Jiang et al., 2025) |
| Nano-/microplastics | Microplastics / nanoplastics | Reed | Whole plant | Controlled exposure | Auxin, gibberellins | Large microplastics promoted growth by 11.87–23.47%, associated with auxin and GA upregulation | Size-dependent shift from oxidative injury toward growth-promoting signaling under larger particles | Growth stimulation under specific particle-size conditions | (Wang et al., 2025) |
| Polyethylene micro-/nanoplastics | Microplastics / nanoplastics | Lettuce | Whole plant | Controlled exposure | Auxin, ABA, growth-regulating hormones | Size-dependent hormonal effects reported | Smaller particles likely intensified oxidative disruption, whereas larger particles elicited weaker signaling perturbation | Differential growth and metabolic responses according to particle size | (Zhang et al., 2024b) |
| Polyethylene microplastics | Microplastics | Garden pea | Whole plant | Controlled exposure | Auxin, gibberellins, ABA | Hormesis-like pattern reported; low-dose growth stimulation and stress at higher exposure | Low ROS burden likely stimulated growth hormones, whereas higher burden shifted signaling toward stress adaptation | Biphasic growth response | (Xiong et al., 2025) |
| Polypropylene and polyethylene microplastics | Microplastics | Tobacco | Roots and shoots | Soil / substrate exposure | Auxin, ABA, defense signaling | Hormone pathways perturbed | Plastics altered growth–defense balance through tissue-dependent molecular responses | Altered growth and molecular stress responses | (Arshad et al., 2025) |
| PET microplastics | Microplastics | Tomato | Roots | Seedling exposure | Auxin | Lateral root inhibition linked to auxin disruption; quantitative partitioning attributed 32.4% of variation to IAA homeostasis disruption under large microplastics | Large-particle effects were more strongly explained by auxin dysregulation than by direct oxidative collapse | Reduced lateral rooting and altered root architecture | (Cui et al., 2025) |
| Microcystins | Cyanotoxin / emerging aquatic contaminant | Rice | Whole plant | Aquatic / hydroponic | Auxin, GA3, ABA | At 1 μg L⁻¹, growth was promoted via increased IAA and GA3; at 10–1000 μg L⁻¹, inhibition was associated with elevated ABA | Clear ROS-threshold hormesis: low exposure maintained signaling, whereas higher exposure triggered ABA-dominated stress physiology | Growth stimulation at low dose and inhibition at higher dose | (Liang and Liu, 2020) |
| Microcystin-LR | Cyanotoxin / emerging aquatic contaminant | *Vallisneria natans* | Whole plant | Aquatic exposure | ABA, strigolactone interaction | Hormonal crosstalk reported | Stress adaptation involved coordinated interaction between ABA and strigolactone signaling | Altered aquatic plant stress physiology | (Li et al., 2022b) |
| Silver nanoparticles | Nanomaterials | *Arabidopsis thaliana* | Whole seedling / likely root-sensitive | Sterile agar plates | JA, stress hormones | JA increased within the broader 13.1–14.7-fold induction range reported for strong defense activation; additional stress hormone perturbation reported | AgNP exposure altered cytoskeletal integrity and activated ROS-linked stress hormone signaling | Developmental disturbance with strong defense activation | (Angelini et al., 2022) |
| Silver nanoparticles | Nanomaterials | Rice | Whole plant | Controlled exposure | Ethylene | Ethylene-mediated NO regulation reported | Nanoparticle stress mobilized ethylene-centered signaling linked to redox and nitric oxide integration | Stress signaling and tolerance modulation | (Tripathi et al., 2023) |
| ZnO nanoparticles under arsenic stress | Nanomaterials / mitigation context | Soybean | Whole plant | Controlled exposure | Gibberellins, ABA, stress-related hormones | GA increased by 66.2–160% under mitigation context; ABA modulation also reported | Nanoparticle-mediated redox buffering partially restored growth signaling under metalloid stress | Improved stress tolerance and growth recovery | (Asad et al., 2024) |
| CuO nanoparticles | Nanomaterials | Rice | Whole plant | Hydroponic | Ethylene, brassinosteroids | Hormonal crosstalk reported | Antagonistic or compensatory BR–ethylene interactions modulated ROS intensity under nanoparticle stress | Modulation of toxicity and defense capacity | (Azhar et al., 2025) |
| Silver nanoparticles | Nanomaterials | Tomato | Whole plant | Controlled exposure | Multiple phytohormones | Hormonal modulation reported | Nanoparticles altered nutrient–hormone interactions and broader stress physiology | Growth and nutrient-response modulation | (Guzmán-Báez et al., 2026) |
| Selenium and ZnO nanoparticles | Nanomaterials / mitigation | *Brassica napus* | Seedlings | Salt-stress context | Auxin, gibberellins, ABA | Hormone rebalancing reported | Nanoparticle treatments buffered stress by restoring redox–hormone coordination | Improved germination and early growth | (El-Badri et al., 2021) |
| Cobalt nanoparticles | Nanomaterial | *Lemna minor* | Whole plant, with strong chloroplast involvement | Hydroponic exposure in ASM1 medium | ROS-associated stress signaling pathways (ABA/JA-related responses inferred, not directly quantified) | Hormonal disruption not directly measured; oxidative-stress-mediated signaling inferred | CoNPs induced severe ROS overproduction through nanoparticle surface redox activity and intracellular Co²⁺ release, causing chloroplast penetration, thylakoid disorganization, plastoglobuli accumulation, and membrane destabilization, likely triggering secondary ROS–hormone crosstalk | Strong oxidative stress, chloroplast ultrastructural damage, photosynthetic impairment, reduced growth, and metabolic disruption | (Mallin et al., 2025) |
| Microplastics with selenium rescue | Microplastics / mitigation | Kale | Whole plant | Controlled exposure | ABA, auxin, stress hormones | Rescue-associated hormonal normalization reported | Selenium likely improved antioxidant capacity and stabilized hormonal homeostasis | Partial reversal of contaminant stress | (Tong et al., 2024) |
| Microplastics | Microplastics | *Pinellia ternata* | Whole plant | Controlled exposure | Brassinosteroid-related protection | Protective hormonal modulation reported | Brassinolide alleviated oxidative and endocrine perturbation caused by microplastics | Improved physiological tolerance | (Zhang et al., 2024a) |
| Microplastics | Microplastics | *Pinellia ternata* | Whole plant | Controlled exposure | Auxin | Auxin-mediated protection reported | Exogenous or preserved auxin signaling mitigated oxidative injury and restored growth control | Reduced stress and improved performance | (Zhang et al., 2025) |
| Polyethylene nanoplastics + lead with fungal mitigation | Nanoplastics + metal | Rice | Whole plant | Controlled exposure | Hormone signaling broadly | Hormonal rescue under mitigation reported | Biological mitigation likely reduced ROS burden and restored hormonal balance | Improved stress tolerance under co-exposure | (Peng et al., 2026) |

ABA, abscisic acid; EECs, emerging environmental contaminants; GA, gibberellin(s); IAA, indole-3-acetic acid; JA, jasmonic acid; NO, nitric oxide; PFAS, per- and polyfluoroalkyl substances; PFOS, perfluorooctane sulfonate; PMMA, polymethyl methacrylate; PS, polystyrene; ROS, reactive oxygen species.

**Table S5**. Climate–Emerging Contaminant Interactions in Plant Systems

| Climate factor | Contaminant(s) | Plant species / system | Experimental context | Main physiological response | Climate-dependent toxicity shift | Uptake / accumulation | Biotransformation | Sorption / retention | Main implication | Reference |
| --- | --- | --- | --- | --- | --- | --- | --- | --- | --- | --- |
| Temperature (10, 15, 21 °C) | Atrazine; S-metolachlor | *Spirodela polyrhiza* | Aquatic lab exposure | Photosynthesis more sensitive than growth; APX shifts | Higher toxicity at 10 °C | NR | Temperature-dependent removal | NR | Low T reduces removal efficiency | (Cruz et al., 2025b) |
| Temperature + UV | Antibiotic mixture | *Salvinia molesta*; *Myriophyllum aquaticum* | Factorial aquatic experiment | Species-specific photophysiology changes | Synergistic/antagonistic interactions depending on T | Retention in Salvinia | Higher degradation in Myriophyllum | Shading-mediated retention | Morphotype controls fate | (Castilho et al., 2025) |
| Elevated CO_2_ | Nanoplastics | *Hordeum vulgare* | Chamber experiment | Redox and respiration changes | Reduced oxidative stress under eCO2 | Increased accumulation | NR | NR | Decoupling of stress and accumulation | (Jian et al., 2023) |
| Drought | PVC microplastics | *Oryza sativa* | Soil co-exposure | Metabolome and proteome disruption | Stronger effects under combined stress | NR | Altered metabolism | NR | Reduced tolerance under drought | (Khan et al., 2024) |
| Temperature/light | Pesticides | Aquatic macrophytes | Meta-analysis | Enhanced removal with plants | Higher removal under warm/light conditions | NR | NR | NR | Plants increase removal efficiency | (Cruz et al., 2025a) |

**References**

Abdelmoneim, M. S., Hafez, E. E., Dawood, M. F. A., Hammad, S. F., and Ghazy, M. A. (2024). Toxicity of bisphenol A and p -nitrophenol on tomato plants: Morpho-physiological, ionomic profile, and antioxidants/defense-related gene expression studies. *Biomol. Concepts* 15. doi: 10.1515/bmc-2022-0049

Adhikari, S., Adhikari, A., Ghosh, S., Roy, D., Azahar, I., Basuli, D., et al. (2020). Assessment of ZnO-NPs toxicity in maize: An integrative microRNAomic approach. *Chemosphere* 249, 126197. doi: 10.1016/j.chemosphere.2020.126197

Angelini, J., Klassen, R., Široká, J., Novák, O., Záruba, K., Siegel, J., et al. (2022). Silver Nanoparticles Alter Microtubule Arrangement, Dynamics and Stress Phytohormone Levels. *Plants* 11, 313. doi: 10.3390/plants11030313

Arikan, B., Alp, F. N., Ozfidan-Konakci, C., Yildiztugay, E., Turan, M., and Cavusoglu, H. (2022). The impacts of nanoplastic toxicity on the accumulation, hormonal regulation and tolerance mechanisms in a potential hyperaccumulator - Lemna minor L. *J. Hazard. Mater.* 440, 129692. doi: 10.1016/j.jhazmat.2022.129692

Arshad, M., Zhou, Z., Zhang, Y., Lin, S., Shoaib, M., Zhang, H., et al. (2025). Unraveling the impact of nano-microscale polyethylene and polypropylene plastics on Nicotiana tabacum: Physiological responses and molecular mechanisms. *Environ. Exp. Bot.* 236, 106169. doi: 10.1016/j.envexpbot.2025.106169

Asad, M., Khan, Z., Shah, T., Shah, M. A., Imran, A., Rasool, S., et al. (2024). Mitigation of microplastic toxicity in soybean by synthetic bacterial community and arbuscular mycorrhizal fungi interaction: Altering carbohydrate metabolism, hormonal transduction, and genes associated with lipid and protein metabolism. *Plant Stress* 14, 100631. doi: 10.1016/j.stress.2024.100631

Azhar, W., Salam, A., Khan, A. R., Ahmad, I., and Gan, Y. (2025). Brassinosteroids Alleviate Ethylene-Induced Copper Oxide Nanoparticle Toxicity and Ultrastructural and Stomatal Damage in Rice Seedlings. *Agriculture* 15, 907. doi: 10.3390/agriculture15080907

Bahmani, R., Kim, D., Modareszadeh, M., Thompson, A. J., Park, J. H., Yoo, H. H., et al. (2020). The mechanism of root growth inhibition by the endocrine disruptor bisphenol A (BPA). *Environ. Pollut.* 257, 113516. doi: 10.1016/j.envpol.2019.113516

Castilho, B. M., Simões, L. de C., Soares, M. da S., Carneiro, D. N. M., and Gomes, M. P. (2025). Interactive effects of temperature and UV radiation on antibiotic uptake and degradation in a floating (Salvinia molesta) and a submerged/emergent (Myriophyllum aquaticum) macrophyte. *J. Hazard. Mater.* 496, 139366. doi: 10.1016/j.jhazmat.2025.139366

Chakraborty, P., Paul, N., Christudoss, A. C., and Mukherjee, A. (2026). Graphene oxide nanomaterials reduce tetrabromobisphenol-A accumulation and attenuate phytotoxicity in Allium cepa. *Plant Nano Biol.* 15, 100257. doi: 10.1016/j.plana.2026.100257

Chen, L., Xu, Z., He, Y., Zhang, X., Li, L., Zhu, R., et al. (2025a). Multiomics Analysis Reveals Key Targeted Metabolic Pathways Underlying the Hormesis and Detrimental Effects of Enrofloxacin on Rice Plants. *J. Agric. Food Chem.* 73, 2678–2695. doi: 10.1021/acs.jafc.4c09001

Chen, X., Chen, J., Zhang, Y., Ling, C., and Shen, Y. (2025b). Biochar Nanoparticles Reduce Ciprofloxacin Accumulation and Restore Growth and Hormonal Balance in Rice Seedlings. *Plants* 14, 380. doi: 10.3390/plants14030380

Choe, H., Kantharaj, V., Lee, K.-A., Shin, Y., Chohra, H., Yoon, Y.-E., et al. (2024). Morpho-physiological, biochemical, and molecular responses of lettuce (Lactuca sativa L.) seedlings to chlortetracycline stress. *Environ. Exp. Bot.* 219, 105615. doi: 10.1016/j.envexpbot.2023.105615

Cruz, F. V. da S., Cardoso, S. J., and Juneau, P. (2025a). Phytoremediation of pesticide-contaminated freshwaters by aquatic plants: a meta-analysis. *Chemosphere* 383, 144465. doi: 10.1016/j.chemosphere.2025.144465

Cruz, F. V. da S., Venne, P., Segura, P., and Juneau, P. (2025b). Effect of temperature on the physiology and phytoremediation capacity of Spirodela polyrhiza exposed to atrazine and S-metolachlor. *Aquat. Toxicol.*, 107304. doi: 10.1016/j.aquatox.2025.107304

Cui, C., Yang, W., Dang, W., Chen, R., García-Caparrós, P., Yang, G., et al. (2025). Bamboo Biochar and Sodium Silicate Alleviate Oxybenzone-Induced Phytotoxicity via Distinct Mechanisms for Sustainable Plant Protection. *Plants* 14, 2382. doi: 10.3390/plants14152382

Dal Ferro, N., Pellizzaro, A., Fant, M., Zerlottin, M., and Borin, M. (2021). Uptake and translocation of perfluoroalkyl acids by hydroponically grown lettuce and spinach exposed to spiked solution and treated wastewaters. *Sci. Total Environ.* 772, 145523. doi: 10.1016/j.scitotenv.2021.145523

El-Badri, A. M., Batool, M., Wang, C., Hashem, A. M., Tabl, K. M., Nishawy, E., et al. (2021). Selenium and zinc oxide nanoparticles modulate the molecular and morpho-physiological processes during seed germination of Brassica napus under salt stress. *Ecotoxicol. Environ. Saf.* 225, 112695. doi: 10.1016/j.ecoenv.2021.112695

Fan, T., Chen, X., Xu, Z., Liu, L., Shen, D., Dong, S., et al. (2020). Uptake and Translocation of Triflumezopyrim in Rice Plants. *J. Agric. Food Chem.* 68, 7086–7092. doi: 10.1021/acs.jafc.9b07868

Fuchs, B., Laihonen, M., Muola, A., Saikkonen, K., Dobrev, P. I., Vankova, R., et al. (2022). A Glyphosate-Based Herbicide in Soil Differentially Affects Hormonal Homeostasis and Performance of Non-target Crop Plants. *Front. Plant Sci.* 12. doi: 10.3389/fpls.2021.787958

Gao, M., Wang, Z., Jia, Z., Zhang, H., and Wang, T. (2023). Brassinosteroids alleviate nanoplastic toxicity in edible plants by activating antioxidant defense systems and suppressing nanoplastic uptake. *Environ. Int.* 174, 107901. doi: 10.1016/j.envint.2023.107901

Ge, J., Cheng, J., Li, Y., Li, Q. X., and Yu, X. (2020). Effects of dibutyl phthalate contamination on physiology, phytohormone homeostasis, rhizospheric and endophytic bacterial communities of Brassica rapa var. chinensis. *Environ. Res.* 189, 109953. doi: 10.1016/j.envres.2020.109953

Gomes, M. P., Brito, J. C. M., Rocha, D. C., Navarro-Silva, M. A., and Juneau, P. (2020). Individual and combined effects of amoxicillin, enrofloxacin, and oxytetracycline on *Lemna minor* physiology. *Ecotoxicol. Environ. Saf.* 203, 111025. doi: 10.1016/j.ecoenv.2020.111025

Gomes, M. P., Freitas, P. L., Kitamura, R. S. A., Pereira, E. G., and Juneau, P. (2022). How aminomethylphosphonic acid (AMPA), the main glyphosate metabolite, interferes with chlorophyll biosynthesis? *Environ. Exp. Bot.* 203, 105039. doi: 10.1016/j.envexpbot.2022.105039

Gong, W., Zhang, W., Jiang, M., Li, S., Liang, G., Bu, Q., et al. (2021). Species-dependent response of food crops to polystyrene nanoplastics and microplastics. *Sci. Total Environ.* 796, 148750. doi: 10.1016/j.scitotenv.2021.148750

Groffen, T., Kuijper, N., Oden, S., Willems, T., Bervoets, L., and Prinsen, E. (2023). Growth Hormones in Broad Bean (Vicia faba L.) and Radish (Raphanus raphanistrum subsp. sativus L.) Are Associated with Accumulated Concentrations of Perfluoroalkyl Substances. *Toxics* 11, 922. doi: 10.3390/toxics11110922

Gu, Q., Wen, Y., Wu, H., and Cui, X. (2023). Uptake and translocation of both legacy and emerging per- and polyfluorinated alkyl substances in hydroponic vegetables. *Sci. Total Environ.* 862, 160684. doi: 10.1016/j.scitotenv.2022.160684

Guzmán-Báez, G. A., Trejo-Téllez, L. I., Navarro-López, D. E., Mejía-Méndez, J. L., and Gómez-Merino, F. C. (2026). Influence of Silver Nanoparticles (AgNPs) on Vegetative Growth and Concentrations of Nutrients and Phytohormones in Tomato. *Plants* 15, 405. doi: 10.3390/plants15030405

Hu, Y., Habibul, N., Hu, Y.-Y., Meng, F.-L., and Sheng, G.-P. (2021). Chemical speciation of ciprofloxacin in aqueous solution regulates its phytotoxicity and uptake by rice (Oryza sativa L.). *Sci. Total Environ.* 771, 144787. doi: 10.1016/j.scitotenv.2020.144787

Huang, C., Liu, Y., Lu, J., Deng, X., Liu, Z., Molloy, D. P., et al. (2025). Integrated physiological, transcriptomics, and metabolite analysis reveal photosynthetic toxicity related to the bisphenol A stress response mechanism in pepper (Capsicum annuum L.). *Environ. Pollut.* 381, 126530. doi: 10.1016/j.envpol.2025.126530

Jian, S., Li, S., Liu, F., Liu, S., Gong, L., Jiang, Y., et al. (2023). Elevated atmospheric CO2 concentration changes the eco-physiological response of barley to polystyrene nanoplastics. *Chem. Eng. J.* 457, 141135. doi: 10.1016/j.cej.2022.141135

Jiang, L., Liu, Z., Li, W., Yang, Y., Yu, Z., Fan, J., et al. (2025). PVC Inhibits Radish (Raphanus sativus L.) Seedling Growth by Interfering with Plant Hormone Signal Transduction and Phenylpropanoid Biosynthesis. *Horticulturae* 11, 896. doi: 10.3390/horticulturae11080896

Kafle, A., Timilsina, A., Gautam, A., Adhikari, K., Bhattarai, A., and Aryal, N. (2022). Phytoremediation: Mechanisms, plant selection and enhancement by natural and synthetic agents. *Environ. Adv.* 8, 100203. doi: 10.1016/j.envadv.2022.100203

Khan, K. Y., Ali, B., Ghani, H. U., Cui, X., Luo, X., Ali, Z., et al. (2024). Polyvinyl chloride microplastics and drought co-exposure alter rice growth by affecting metabolomics and proteomics. *Sci. Total Environ.* 955, 177002. doi: 10.1016/j.scitotenv.2024.177002

Kochi, L. Y., Kitamura, R. S. A., Rocha, C. S., Brito, J. C. M., Juneau, P., and Gomes, M. P. (2023). Synergistic Removal of Ciprofloxacin and Sulfamethoxazole by Lemna minor and Salvinia molesta in Mixed Culture: Implications for Phytoremediation of Antibiotic-Contaminated Water. *Water* 15, 1899. doi: 10.3390/w15101899

Leitão, I., Leclercq, C. C., Ribeiro, D. M., Renaut, J., Almeida, A. M., Martins, L. L., et al. (2021a). Stress response of lettuce (Lactuca sativa) to environmental contamination with selected pharmaceuticals: A proteomic study. *J. Proteomics* 245, 104291. doi: 10.1016/j.jprot.2021.104291

Leitão, I., Mourato, M. P., Carvalho, L., Oliveira, M. C., Marques, M. M., and Martins, L. L. (2021b). Antioxidative response of lettuce (Lactuca sativa) to carbamazepine-induced stress. *Environ. Sci. Pollut. Res.* 28, 45920–45932. doi: 10.1007/s11356-021-13979-3

Li, J., Sun, J., and Li, P. (2022a). Exposure routes, bioaccumulation and toxic effects of per- and polyfluoroalkyl substances (PFASs) on plants: A critical review. *Environ. Int.* 158, 106891. doi: 10.1016/j.envint.2021.106891

Li, L., Luo, Y., Li, R., Zhou, Q., Peijnenburg, W. J. G. M., Yin, N., et al. (2020a). Effective uptake of submicrometre plastics by crop plants via a crack-entry mode. *Nat. Sustain.* 3, 929–937. doi: 10.1038/s41893-020-0567-9

Li, P., Xiao, Z., Sun, J., Oyang, X., Xie, X., Li, Z., et al. (2020b). Metabolic regulations in lettuce root under combined exposure to perfluorooctanoic acid and perfluorooctane sulfonate in hydroponic media. *Sci. Total Environ.* 726, 138382. doi: 10.1016/j.scitotenv.2020.138382

Li, Q., Tian, X., Gu, P., Yang, G., Deng, H., Zhang, J., et al. (2022b). Transcriptomic analysis reveals phytohormone and photosynthetic molecular mechanisms of a submerged macrophyte in response to microcystin-LR stress. *Aquat. Toxicol.* 245, 106119. doi: 10.1016/j.aquatox.2022.106119

Liang, C., and Liu, H. (2020). Response of hormone in rice seedlings to irrigation contaminated with cyanobacterial extract containing microcystins. *Chemosphere* 256, 127157. doi: 10.1016/j.chemosphere.2020.127157

Liu, Y., Wei, L., Yu, H., Cao, X., Peng, J., Liu, H., et al. (2022). Negative impacts of nanoplastics on the purification function of submerged plants in constructed wetlands: Responses of oxidative stress and metabolic processes. *Water Res.* 227, 119339. doi: 10.1016/j.watres.2022.119339

Lu, S., Huo, Z., Niu, T., Zhu, W., Wang, J., Wu, D., et al. (2023). Molecular mechanisms of toxicity and detoxification in rice (Oryza sativa L.) exposed to polystyrene nanoplastics. *Plant Physiol. Biochem.* 199, 107605. doi: 10.1016/j.plaphy.2023.02.035

Lu, X., Gao, P., Chen, Q., Lin, Z., Song, Z., and Lei, M. (2025). The response of Chinese Cabbage (Brassica rapa L.) to the co-contamination of nanoplastics with different polarity and Ketoprofen. *Environ. Technol. Innov.* 38, 104188. doi: 10.1016/j.eti.2025.104188

Ma, S., Hua, Z., Zhou, Q., Meng, S., Qiu, X., Ding, L., et al. (2026). Disruption of auxin homeostasis by negatively charged nanoplastics inhibits plant primary root development. *J. Hazard. Mater.* 503, 141140. doi: 10.1016/j.jhazmat.2026.141140

Malea, P., Kokkinidi, D., Kevrekidou, A., and Adamakis, I.-D. S. (2022). The Enzymatic and Non-Enzymatic Antioxidant System Response of the Seagrass Cymodocea nodosa to Bisphenol-A Toxicity. *Int. J. Mol. Sci.* 23, 1348. doi: 10.3390/ijms23031348

Mallin, A. C., Marques, R. Z., Akiyama Kitamura, R. S., Prodocimo, M. M., César de Moraes, L., Figueredo, C. C., et al. (2025). Lemna minor as a phytoremediator of cobalt nanoparticles: Insights into toxicity and remediation. *Water Biol. Secur.*, 100442. doi: 10.1016/j.watbs.2025.100442

McGinnis, M., Sun, C., Dudley, S., and Gan, J. (2019). Effect of low-dose, repeated exposure of contaminants of emerging concern on plant development and hormone homeostasis. *Environ. Pollut.* 252, 706–714. doi: 10.1016/j.envpol.2019.05.159

Ozfidan-Konakci, C., Yildiztugay, E., Arikan, B., Alp-Turgut, F. N., Turan, M., Cavusoglu, H., et al. (2023). Responses of individual and combined polystyrene and polymethyl methacrylate nanoplastics on hormonal content, fluorescence/photochemistry of chlorophylls and ROS scavenging capacity in Lemna minor under arsenic-induced oxidative stress. *Free Radic. Biol. Med.* 196, 93–107. doi: 10.1016/j.freeradbiomed.2023.01.015

Peng, J., Yan, Q., Hassan, M. U., Imran, M., Haider, F. U., Liang, J., et al. (2026). Metarhizium anisopliae Mitigates the Phytotoxicity of Lead and Nanoplastics on Rice by Modifying Physiological, Transcriptomic, Metabolomic Activities, and Soil Microbiome. *Adv. Sci.* 13. doi: 10.1002/advs.202521570

Qiu, G., Han, Z., Wang, Q., Wang, T., Sun, Z., Yu, Y., et al. (2023). Toxicity effects of nanoplastics on soybean (Glycine max L.): Mechanisms and transcriptomic analysis. *Chemosphere* 313, 137571. doi: 10.1016/j.chemosphere.2022.137571

Rao, M. J., Duan, M., Ikram, M., and Zheng, B. (2025). ROS Regulation and Antioxidant Responses in Plants Under Air Pollution: Molecular Signaling, Metabolic Adaptation, and Biotechnological Solutions. *Antioxidants* 14, 907. doi: 10.3390/antiox14080907

Shoaib, M., Zhang, H., Hussain, S., Arshad, M., Brestic, M., Yang, X., et al. (2025). Integrated physiological, metabolomic, and transcriptomic responses of maize (Zea mays) and soybean (Glycine max) to nanoplastic-induced stress. *Ecotoxicol. Environ. Saf.* 302, 118612. doi: 10.1016/j.ecoenv.2025.118612

Sun, H., Lei, C., Xu, J., and Li, R. (2021). Foliar uptake and leaf-to-root translocation of nanoplastics with different coating charge in maize plants. *J. Hazard. Mater.* 416, 125854. doi: 10.1016/j.jhazmat.2021.125854

Tang, X., Hao, F., Yuan, H., Yan, X., Yang, D., and Taylor, A. G. (2020). Uptake and translocation of imidacloprid via seed pathway and root pathway during early seedling growth of corn. *Pest Manag. Sci.* 76, 3792–3799. doi: 10.1002/ps.5930

Tong, M., Zhai, K., Duan, Y., Xia, W., Zhao, B., Zhang, L., et al. (2024). Selenium alleviates the adverse effects of microplastics on kale by regulating photosynthesis, redox homeostasis, secondary metabolism and hormones. *Food Chem.* 450, 139349. doi: 10.1016/j.foodchem.2024.139349

Tripathi, D. K., Kandhol, N., Rai, P., Mishra, V., Pandey, S., Deshmukh, R., et al. (2023). Ethylene Renders Silver Nanoparticles Stress Tolerance in Rice Seedlings by Regulating Endogenous Nitric Oxide Accumulation. *Plant Cell Physiol.* 63, 1954–1967. doi: 10.1093/pcp/pcac159

Voloshina, M., Rajput, V., Minkina, T., Vechkanov, E., Mandzhieva, S., Mazarji, M., et al. (2022). Zinc Oxide Nanoparticles: Physiological and Biochemical Responses in Barley (Hordeum vulgare L.). *Plants* 11, 2759. doi: 10.3390/plants11202759

Wang, T.-T., Ying, G.-G., Shi, W.-J., Zhao, J.-L., Liu, Y.-S., Chen, J., et al. (2020). Uptake and Translocation of Perfluorooctanoic Acid (PFOA) and Perfluorooctanesulfonic Acid (PFOS) by Wetland Plants: Tissue- and Cell-Level Distribution Visualization with Desorption Electrospray Ionization Mass Spectrometry (DESI-MS) and Transmission Ele. *Environ. Sci. Technol.* 54, 6009–6020. doi: 10.1021/acs.est.9b05160

Wang, W., Yuan, S., and Kwon, J.-H. (2022). Insight into the uptake and translocation of per- and polyfluoroalkyl substances in hydroponically grown lettuce. *Environ. Sci. Pollut. Res.* 29, 85454–85464. doi: 10.1007/s11356-022-21886-4

Wang, Y., Bai, J., Zhang, Y., Zhai, Y., Qiu, J., and Wang, X. (2025). Nanoplastics, but Not Microplastics, Enhance Plant Resistance to Herbivores Through Defence Priming. *Plant. Cell Environ.* doi: 10.1111/pce.70325

Xie, Z., Zeng, Y., Zhu, S., Zhu, M., Yu, Y., Cheng, K., et al. (2026). From energy collapse to chemical defense: Microplastics reshape the metabolic landscape of Tetrastigma hemsleyanum (Vitaceae). *Plant Physiol. Biochem.* 231, 111007. doi: 10.1016/j.plaphy.2025.111007

Xiong, Z., Zhang, N., Li, H., Liu, C., Liu, Y., Peng, L., et al. (2025). Integrated metabolomics and transcriptomics reveal the hormesis-like effects of polyethylene microplastics on Pisum sativum L. *Environ. Technol. Innov.* 37, 103972. doi: 10.1016/j.eti.2024.103972

Xu, C. (2024). The Oryza sativa transcriptome responds spatiotemporally to polystyrene nanoplastic stress. *Sci. Total Environ.* 928, 172449. doi: 10.1016/j.scitotenv.2024.172449

Xu, L., Liu, C., Ren, Y., Huang, Y., Liu, Y., Feng, S., et al. (2024). Nanoplastic toxicity induces metabolic shifts in Populus × euramericana cv. “74/76” revealed by multi-omics analysis. *J. Hazard. Mater.* 470, 134148. doi: 10.1016/j.jhazmat.2024.134148

Yildiztugay, E., Ozfidan-Konakci, C., Arikan, B., Alp, F. N., Elbasan, F., Zengin, G., et al. (2022). The hormetic dose-risks of polymethyl methacrylate nanoplastics on chlorophyll a fluorescence transient, lipid composition and antioxidant system in Lactuca sativa. *Environ. Pollut.* 308, 119651. doi: 10.1016/j.envpol.2022.119651

Yu, C., Zeng, H., Wang, Q., Chen, W. W., Chen, W. W., Yu, W., et al. (2022). Multi-omics analysis reveals the molecular responses of Torreya grandis shoots to nanoplastic pollutant. *J. Hazard. Mater.* 436, 129181. doi: 10.1016/j.jhazmat.2022.129181

Zhang, D., Duan, Y., Xia, W., Zhai, K., Zhao, B., Zhang, L., et al. (2025). Indoleacetic acid protection against microplastic induced oxidative stress in Pinellia ternata antioxidant enzyme and secondary metabolite regulation. *Physiol. Plant.* 177. doi: 10.1111/ppl.70253

Zhang, D., Zhang, L., Yuan, C., Zhai, K., Xia, W., Duan, Y., et al. (2024a). Brassinolide as potential rescue agent for Pinellia ternata grown under microplastic condition: Insights into their modulatory role on photosynthesis, redox homeostasis, and AsA-GSH cycling. *J. Hazard. Mater.* 470, 134116. doi: 10.1016/j.jhazmat.2024.134116

Zhang, L., Vaccari, F., Ardenti, F., Fiorini, A., Tabaglio, V., Puglisi, E., et al. (2024b). The dosage- and size-dependent effects of micro- and nanoplastics in lettuce roots and leaves at the growth, photosynthetic, and metabolomics levels. *Plant Physiol. Biochem.* 208, 108531. doi: 10.1016/j.plaphy.2024.108531

Zhang, T., Li, N., Chen, G., Xu, J., Ouyang, G., and Zhu, F. (2021). Stress symptoms and plant hormone-modulated defense response induced by the uptake of carbamazepine and ibuprofen in Malabar spinach (Basella alba L.). *Sci. Total Environ.* 793, 148628. doi: 10.1016/j.scitotenv.2021.148628
